# Therapeutic strategy targeting host lipolysis limits infection by SARS-CoV-2 and influenza A virus

**DOI:** 10.1038/s41392-022-01223-4

**Published:** 2022-10-17

**Authors:** Yeong-Bin Baek, Hyung-Jun Kwon, Muhammad Sharif, Jeongah Lim, In-Chul Lee, Young Bae Ryu, Jae-In Lee, Ji-Sun Kim, Young-Seung Lee, Dong-Hoon Kim, Sang-Ik Park, Don-Kyu Kim, Jeong-Sun Kim, Hyon E. Choy, Sunwoo Lee, Hueng-Sik Choi, Timothy F. Osborne, Tae-Il Jeon, Kyoung-Oh Cho

**Affiliations:** 1 Laboratory of Veterinary Pathology, College of Veterinary Medicine, Chonnam National University, Gwangju, 61186 Republic of Korea; 2 Functional Biomaterial Research Center, Korea Research Institute of Bioscience & Biotechnology, Jeongeup-si, Jeollabuk-do 56212 Republic of Korea; 3 Department of Chemistry, Chonnam National University, Gwangju, 61186 Republic of Korea; 4 Korean Collection for Type Cultures, Korea Research Institute of Bioscience & Biotechnology, Jeongeup-si, Jeollabuk-do 56212 Republic of Korea; 5 Department of Animal Science, Chonnam National University, Gwangju, 61186 Republic of Korea; 6 Department of Pharmacology, Korea University College of Medicine, Seoul, 02841 Republic of Korea; 7 Department of Integrative Food, Bioscience and Biotechnology, Chonnam National University, Gwangju, 61186 Republic of Korea; 8 Department of Microbiology, Chonnam National University Medical School, Gwangju, 61486 Republic of Korea; 9 School of Biological Sciences and Technology, Chonnam National University, Gwangju, 61186 Republic of Korea; 10 Institute for Fundamental Biomedical Research, Department of Medicine and Biological Chemistry, Johns Hopkins University School of Medicine, St. Petersburg, FL 33701 USA

**Keywords:** Industrial microbiology, Infectious diseases

## Abstract

The biosynthesis of host lipids and/or lipid droplets (LDs) has been studied extensively as a putative therapeutic target in diverse viral infections. However, directly targeting the LD lipolytic catabolism in virus-infected cells has not been widely investigated. Here, we show the linkage of the LD-associated lipase activation to the breakdown of LDs for the generation of free fatty acids (FFAs) at the late stage of diverse RNA viral infections, which represents a broad-spectrum antiviral target. Dysfunction of membrane transporter systems due to virus-induced cell injury results in intracellular malnutrition at the late stage of infection, thereby making the virus more dependent on the FFAs generated from LD storage for viral morphogenesis and as a source of energy. The replication of SARS-CoV-2 and influenza A virus (IAV), which is suppressed by the treatment with LD-associated lipases inhibitors, is rescued by supplementation with FFAs. The administration of lipase inhibitors, either individually or in a combination with virus-targeting drugs, protects mice from lethal IAV infection and mitigates severe lung lesions in SARS-CoV-2-infected hamsters. Moreover, the lipase inhibitors significantly reduce proinflammatory cytokine levels in the lungs of SARS-CoV-2- and IAV-challenged animals, a cause of a cytokine storm important for the critical infection or mortality of COVID-19 and IAV patients. In conclusion, the results reveal that lipase-mediated intracellular LD lipolysis is commonly exploited to facilitate RNA virus replication and furthermore suggest that pharmacological inhibitors of LD-associated lipases could be used to curb current COVID-19- and future pandemic outbreaks of potentially troublesome RNA virus infection in humans.

## Introduction

Emerging and reemerging zoonotic viral diseases have occurred periodically in history and are a constant threat to human health globally.^1^ In the last two decades, most of these diseases have been caused by RNA viruses, such as severe acute respiratory syndrome coronavirus 1 (SARS-CoV-1), H1N1 influenza A virus (IAV), Zika virus, Middle East respiratory syndrome coronavirus (MERS-CoV), and, currently, SARS-CoV-2.^1^ RNA viruses present with high genetic diversity even within single infected hosts, enabling rapid adaptation (e.g., following interspecies transmission) and enhanced virulence which eventually leads to the rapid emergence of drug-resistant mutant strains which can significantly limit the effectiveness of vaccines and drugs.^2,3^ Therefore, broad-spectrum antiviral drugs, particularly those targeting host cell machinery critical for viral replication, provide an attractive strategy for targeting pandemic viral diseases that is potentially independent of viral genetic changes.^3^

As obligate intracellular parasites, viruses rely on various host cellular factors and machinery for completing multiple steps of their life cycle, for example, through complex interactions with host cellular lipid metabolism.^4^ Lipid droplets (LDs) have been perceived as mere intracytoplasmic fat inclusion bodies for storage of neutral lipids such as triacylglycerols (TAGs) and cholesterol esters for a long time. However, they have emerged in recent years as highly dynamic, ubiquitous organelles in lipid and energy homeostasis, cell stress and related challenges, immune responses, and antigen cross-presentation.^4^ Many viruses manipulate host lipid metabolism, LD biogenesis, and lipid- and LD-associated immune responses during viral replication and/or assembly which suggests that targeting host lipid metabolism is a potential strategy for host-directed antiviral therapy.^5–7^ Indeed, the pharmacological inhibition of lipogenic transcription factors and enzymes has been widely demonstrated to protect host cells and experimental animals from various viral infections.^8–11^

In the context to LD dynamics during viral infection, there are no detailed studies on how accumulated LDs from the early to the middle stages of viral replication are associated with later stages of infection. These studies may provide, in addition to early lipogenesis and LD formation, a possibility that LDs at a late stage could be re-purposed to serve as lipids that serve as building blocks for the biogenesis of viral replication compartments, viral morphogenesis, or as an energy source for viral replication.^6–11^ However, the regulatory mechanism underlying the lipolytic process which leads to LD breakdown during virus infection has not been studied in detail, possibly enabling new targets for the development of host-directed antiviral therapeutic agents against various viruses. Here, we illuminate the fate of accumulated LDs at the late stage of infections caused by various RNA viruses. Moreover, this study suggests that inhibitors of LD-associated lipases provide a promising new target for broad-spectrum antiviral therapies, including SARS-CoV-2 and its variants and IAV.

## Results

### A common feature of lipase-mediated LD lipolysis in the late stage of replication by diverse RNA viruses

**Fig. 1:**
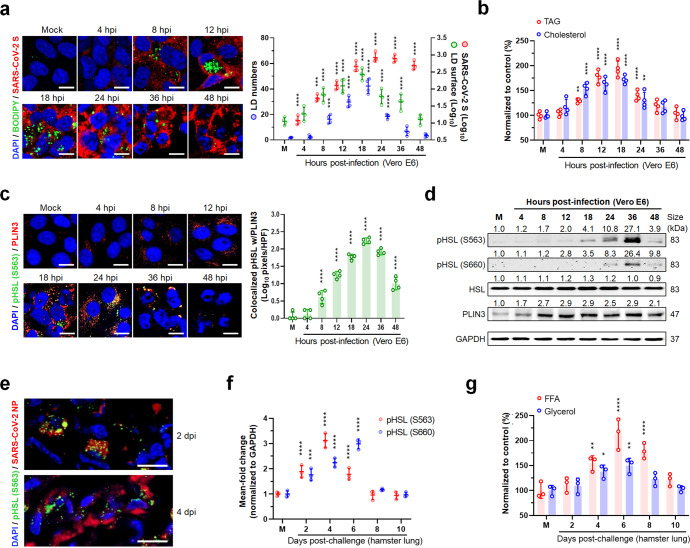
In vitro and in vivo activation of lipid droplet (LD)-associated hormone-sensitive lipase (HSL) during SARS-CoV-2 replication. **a** Representative images (left) and quantification (right) of sequential changes of BODIPY-stained intracellular LDs (green) and SARS-CoV-2 S protein (red) in Vero E6 cells infected with SARS-CoV-2 KCDC03 strain at an MOI of 0.1 FFU. **b** Dynamics of intracellular triacylglycerol (TAG) and cholesterol in the cells infected with SARS-CoV-2 at an MOI of 0.1 FFU. **c** Representative images (left) and quantification (right) of phosphorylated HSL (pHSL, green) colocalized with the LD surface marker perilipin-3 (PLIN3) (red) of cells infected with SARS-CoV-2 at an MOI of 0.1 FFU. **d** Representative western blot. Sequential expression levels of pHSLs (S563 and S660) and PLIN3 in SARS-CoV-2-infected cells at an MOI of 0.1 FFU. **e** Representative images of pHSL (S563, green) and SARS-CoV-2 NP antigen (red) levels in the alveolar epithelial cells of lung tissues sampled at 2 and 4 dpi from Syrian hamsters challenged with 10^5^ TCID_50_ of SARS-CoV-2 KCDC03 strain. **f**, **g** Dynamics of pHSLs (S563 and S660) and intracellular free fatty acids (FFAs) and glycerol in lung tissues obtained sequentially from Syrian hamsters challenged with 10^5^ TCID_50_ of SARS-CoV-2. All data in the graphs are presented as arithmetic means ± SD from four independent experiments with three experimental animals. One-way analysis of variance was carried out with Tukey’s correction for multiple comparisons. *P < 0.05, **P < 0.01, ***P < 0.001, ****P < 0.0001. Scale bars = 50 µm

To evaluate the dynamics of LD formation during viral infection, seven different RNA viruses were individually inoculated into permissive cell lines and evaluated over time. SARS-CoV-2-infected Vero E6 cells showed an increase in LDs and its major components, TAG and cholesterol, up to 18 h post infection (hpi), followed by a decrease in basal levels, as shown by BODIPY staining and lipid content analyses (Fig. 1a, b). We found similar in vitro LD dynamics during the replication of six additional RNA viruses including IAV, bovine coronavirus (BCoV), porcine epidemic diarrhea coronavirus (PEDV), bovine species A rotavirus (RVA), porcine reproductive and respiratory syndrome virus (PRRSV), and porcine sapovirus (PSaV) (Supplementary Fig. 1). Moreover, the alveolar and bronchiolar epithelial cells exhibited a similar pattern of increase and decline in LDs in the lung tissues sampled sequentially from either SARS-CoV-2-challenged Syrian hamsters or IAV-challenged mice (Supplementary Fig. 2). These data indicated that a broad range of RNA viruses, regardless of their genomic structure or the presence/absence of an envelope in the particle, dynamically regulate early LD formation and late breakdown during their life cycle.

LD breakdown is primarily mediated by lipolysis, a process requiring three distinct hydrolases, including adipose triglyceride lipase (ATGL), hormone-sensitive lipase (HSL), and monoacylglycerol lipase.^12^ Interestingly, noticeable increases in phosphorylated HSL (pHSL, S563) colocalized with the LD surface marker perilipin-3 in SARS-CoV-2-infected cells (Fig. 1c). Moreover, the expression levels of pHSLs (phosphorylated at both S563 and S660) determined by a western blot analysis were observed from 8 hpi with a peak at 36 hpi (Fig. 1d). Similar results were obtained for the six other target RNA viruses (Supplementary Fig. 3). Lung tissues obtained sequentially from either SARS-CoV-2-challenged hamsters or IAV-challenged mice also showed significant increases in pHSLs at 4 days post infection (dpi) or six dpi, respectively (Fig. 1e, f and Supplementary Fig. 4a–d). As a consequence of LD lipolysis, intracellular free fatty acid (FFA) and glycerol levels surged at the late stage of viral infections in vivo (Fig. 1g, Supplementary Fig. 4e) and in vitro (Supplementary Fig. 5).

Although LD lipolysis could be governed by multiple signaling events during viral infection, the cyclic AMP (cAMP)/protein kinase A (PKA) signaling pathway is widely known to be the most critical to lipase-mediated LD lipolysis through phosphorylation of HSL.^12^ In response to the infection of SARS-CoV-2 and IAV, the level of cAMP and phosphorylated PKA (pPKA) were increased, particularly at the middle stage of infection (Supplementary Fig. 6a–d) with a concomitant peak of pHSL level (Fig. 1c, d and Supplementary Fig. 3a). Since the cAMP/PKA signaling pathway activates various molecules,^13^ we examined whether the cAMP/PKA signaling pathway participates in the phosphorylation of HSL in SARS-CoV-2- and IAV-infected cells. Of note, simultaneous treatment of virus-infected cells with H89, a PKA inhibitor, prevented viral-mediated HSL phosphorylation (Supplementary Fig. 6e, f). These data suggested that lipase-mediated LD lipolysis in the late stage of viral infection could be triggered by, but not limited to, the cAMP/PKA signaling pathway. Taken together, our data strongly indicated that the disappearance of LDs in the late stage of RNA viral infection is causally correlated with intracellular lipolysis via LD-associated lipases through activation of cAMP/PKA signaling pathway.^12,13^

### In vitro reduction in viral replication and proinflammatory cytokine through lipolysis inhibition

**Fig. 2:**
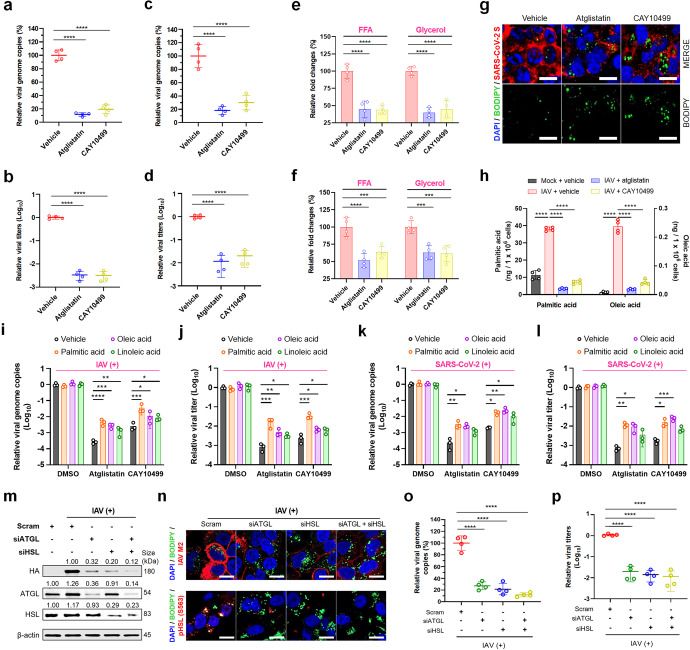
In vitro reduction in viral replication and proinflammatory cytokines through lipolysis inhibition. **a**, **b** Reduction in SARS-CoV-2 genome copy number and infectious progeny production by treatment of SARS-CoV-2-infected Vero E6 cells (MOI = 0.1 FFU) with atglistatin or CAY10499 at 20 µM. **c**, **d** Reduction in IAV genome copy number and infectious progeny production by treatment of IAV-infected A549 cells (MOI = 1 FFU) with 20 µM atglistatin or CAY10499 at 20 µM. **e**, **f** Inhibition of FFA and glycerol release from LDs by the treatment with 20 µM atglistatin or CAY10499 in SARS-CoV-2-infected Vero E6 cells at an MOI of 0.1 (**e**) or IAV-infected A549 cells at an MOI of 1 (**f**). **g** Retention of LDs (green) with corresponding inhibition of SARS-CoV-2 replication (red) by the treatment of virus-infected cells (MOI = 0.1 FFU) with 20 µM atglistatin or CAY10499. **h** Graphical representation of LC-MS data. Reduction in palmitic and oleic acids by treatment of IAV-infected A549 cells (MOI = 1 FFU) with 20 µM atglistatin or CAY10499 at 20 µM. **i**–**l** Increase in viral genome copy numbers and progeny numbers of IAV and SARS-CoV-2 through individual supplementation of exogenous palmitic, oleic, or linoleic acids in the above FFA-deprived condition. **m** Representative western blot. Reduction in IAV HA protein levels in knockdown conditions of *ATGL* and/or *HSL* in A549 cells infected with IAV PR8 strain (MOI = 1 FFU) or mock-infected controls. **n** (upper panels) Retention of LDs (green) with corresponding inhibition of IAV replication (red) by knockdown of *ATGL* and/or *HSL* in IAV-infected A549 cells (MOI = 1 FFU). Lower panels: reduction in pHSL (S563) level by knockdown of *ATGL* and/or *HSL*, resulting in the retention of LDs (green). **o**, **p** Reduction in IAV genome copy numbers and infectious progeny production in IAV-infected A549 cells (MOI = 1 FFU) by knockdown of *ATGL* and/or *HSL*. All data in the graphs are presented as arithmetic means ± SD from four independent experiments. *P < 0.05; **P < 0.01; ***P < 0.001, ****P < 0.0001, one-way analysis of variance with Tukey’s correction for multiple comparisons. Scale bars = 50 µm

We next examined the effects of the non-selective lipase inhibitor CAY10499 and the selective ATGL inhibitor atglistatin on the replication of SARS-CoV-2 and IAV at different treatment times.^14^ In parallel to the increase in lipase activity that occurs in a time-dependent manner (Fig. 1c, d and Supplementary Fig. 3a), the antiviral effect of the CAY10499 and atglistatin at 20 µM concentration in SARS-CoV-2- or IAV-infected cells (MOI of 0.01 FFU) were evident as the treatment time point was closer to median virus incubation time (Supplementary Fig. 7). In addition to inhibiting the replication of both viruses (Fig. 2a–d), treatment of SARS-CoV-2- and IAV-infected cells (MOI of 0.1 FFU) with 20 µM CAY10499 and atglistatin at 18 hpi and 12 hpi, respectively, significantly reduced the levels of intracellular FFAs and glycerol compared to vehicle-treated control (Fig. 2e, f). The inhibition of LD lipolysis in the virus-infected cells resulted in the retention of intracellular LDs (Fig. 2g and Supplementary Fig. 8a).

To examine the role of FFAs in the late stage of virus replication, FFA profiles of mock- and IAV-infected A549 cells were analyzed by a gas chromatography-flame ionization detector (GC-FID). Compared to mock-infected cells, IAV-infected A549 cells at 24 hpi showed a significant increase in the most FFAs, including saturated palmitic acid (PA), monounsaturated oleic acid (OA), and polyunsaturated linolelaidic acid (Supplementary Table 1). Moreover, liquid chromatography-mass spectrometry (LC-MS) analysis showed that PA and OA increased by IAV infection were significantly decreased by treatment of infected cells with both lipase inhibitors (Fig. 2h). Interestingly, supplementation individually with PA, OA, or polyunsaturated linoleic acid (LA) in the FFA-deprived condition in lipase inhibitor-treated cells resulted in a rescue of replication for both IAV (Fig. 2i, j) and SARS-CoV-2 (Fig. 2k, l), an observation consistent with the previous reports.^11,15^ Furthermore, siRNA-mediated gene silencing of *ATGL* and/or *HSL* markedly suppressed the protein expression levels of hemagglutinin (HA) and M2 (active as an ion channel^16^), genome replication, and infectious progeny production through inhibition of LD degradation in IAV-infected A549 cells (Fig. 2m–p).

Activated proinflammatory cytokines including interferon-α (IFN-α), IFN-β, tumor necrosis factor-α (TNF-α), interleukin-6 (IL-6), and monocyte chemoattractant protein-1 (MCP-1) in response to IAV infection were significantly reduced by treatment with atglistatin and CAY10499 (Supplementary Fig. 8b–m). Treatment of SARS-CoV-2-infected Vero E6 cells with atglistatin and CAY10499 in the above conditions resulted in markedly reduced levels of TNF-α, IL-6, and MCP-1 but not IFN-α and IFN-β which was likely due to both the inability of Vero cells to synthesize IFNs and the antagonist role of SARS-CoV-2 proteins against types I and III IFNs (Supplementary Fig. 8b–m).^17,18^ Taken together, these data suggested that both lipase inhibitors could reduce viral replication and proinflammatory cytokine response through blockade of FFA release from LDs.

### Inevitable LD lipolysis for FFA generation during the late stage of viral replication

**Fig. 3:**
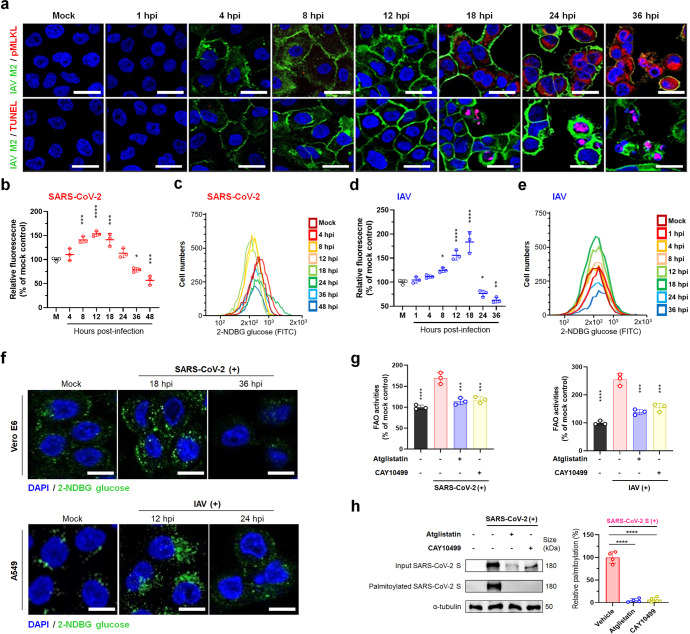
Dysfunction of cellular glucose transporter systems by virus-induced cell injury, leading to release of FFAs from accumulated lipid droplets for viral palmitoylation and fatty acid oxidation (FAO). **a** Sequential changes of necroptotic-marker (pMLKL) positive (upper panels) and apoptotic-marker (TUNEL) positive (lower panels) A549 cells infected with IAV PR8 strain at an MOI of 1 FFU determined by immunofluorescence assay (using an antibody against necroptotic marker pMLKL) or TUNEL assay. **b**, **c** Sequential glucose uptake into Vero E6 cells infected with SARS-CoV-2 KCDC03 strain at an MOI of 0.1 FFU by measurement of fluorescent glucose using a fluorometer and flow cytometry. **d**, **e** Sequential glucose uptake into A549 cells infected with IAV PR8 strain at an MOI of 1 FFU by measurement of fluorescent glucose by a fluorometer and flow cytometry. **f** Uptake of green fluorescent glucose into Vero E6 cells infected with SARS-CoV-2 at an MOI of 0.1 FFU (upper panels) and A549 cells infected with IAV at an MOI of 1 FFU (lower panels) observed by confocal microscopy. **g** Graphical representation of inhibitory effects of atglistatin and CAY10499 on intracellular FAO activities in the SARS-CoV-2- and IAV-infected cells at 36 hpi and 24 hpi, respectively. **h** Representative western blot (left) and quantification (right) of inhibitory effects of atglistatin and CAY10499 on palmitoylation of SARS-CoV-2 S protein in virus-infected Vero E6 cells (MOI = 0.1 FFU) at 36 hpi. All data in the graphs are presented as arithmetic means ± SD from three independent experiments. For statistical analysis, a one-way analysis of variance was carried out with Tukey’s correction for multiple comparisons. *P < 0.05, **P < 0.01, ***P < 0.001, ****P < 0.0001. Scale bars = 30 µm

Most viral infections damage infected cells as a result of viral replication and immunopathological effects and, in severe cases, cause cell death mainly by apoptosis and necrosis.^19,20^ In these compromised settings, transport of nutrients such as glucose through plasma membrane transporters is likely restricted, thereby causing a deficiency of intracellular energy generation. To understand more about the role of LD lipolysis during the late stage of infection with RNA viruses, we first determined cell death dynamics induced by IAV infection. Dual positive cells for the IAV M2 protein and the necroptosis marker, phosphorylated mixed lineage kinase domain-like protein (pMLKL), were visible from 18 hpi and their numbers increased thereafter (Fig. 3a, upper panels). Notably, pMLKL—the major effector—whose accumulation ultimately leads to membrane lysis, was translocated to the inner plasma membrane of IAV-infected cells (Fig. 3a, upper panels).^19^ Moreover, characteristic necroptotic cells showing both plasma membrane rupture and peripheral translocation of pMLKL increased (Fig. 3a, upper panels). Likely, IAV-positive apoptotic cells were detected from 18 hpi and its number increased (Fig. 3a, lower panels).

We next quantified sequential changes of a number of cells expressing either necroptotic (pMLKL) or apoptotic (TUNEL) markers using flow cytometry. Quantification of each cell portion showed that both necroptotic and apoptotic-marker-positive cells started to increase simultaneously, but the percentage gradually increased in necroptotic-marker-positive cells, reaching a proportion of 70%:17% (necroptotic vs apoptotic-marker-positive ratio) at 48 hpi in SARS-CoV-2-infected Vero E6 cells and a proportion of 56%:29% (necroptotic vs apoptotic-marker-positive) at 36 hpi in IAV-infected A549 cells (Supplementary Fig. 9a, b). Moreover, the above results were confirmed by western blotting results that showed the expression level of pMLKL significantly increased from 18 hpi in SARS-CoV-2-infected cells and from 12 hpi in IAV-infected cells, but cleaved caspase 3 accumulated from 24 hpi in SARS-CoV-2-infected cells and from 18 hpi in IAV-infected cells, respectively (Supplementary Fig. 9c, d). We next checked whether both lipase inhibitors reduce virus-induced cell death. Inhibition of LD lipolysis by treatment with both lipase inhibitors significantly decreased necroptotic (pMLKL) and apoptotic (TUNEL) marker-positive cells (Supplementary Fig. 10a, b) along with the marked reduction in both SARS-CoV-2 and IAV replication (Fig. 2). In contrast, supplementation of FFAs in the condition of FFA deficiency by the treatment with lipase inhibitors showed an increase in necroptotic and apoptotic-marker-positive cells (Supplementary Fig. 10c–f) with increased SARS-CoV-2 and IAV replication (Fig. 2i–l).

The above data suggested that both SARS-CoV-2 and IAV may induce dysfunction of cellular machinery, including membrane transporter systems, particularly at the late stage of infection. Therefore, we examined glucose uptake levels in the SARS-CoV-2- or IAV-infected cells at different time points. Interestingly, the increased levels of glucose uptake levels during SARS-CoV-2 and IAV replications decreased substantially at the late stage of viral infection (Fig. 3b–f), suggesting that virus-induced dysfunction of membrane transporter systems, such as the glucose transporter at the late stage of infection, could make the virus more dependent on the FFAs generated from stored LDs by lipolysis. Indeed, infection with both SARS-CoV-2 and IAV significantly increased fatty acid oxidation (FAO) activities at the late stage of infection, which was reduced by lipase inhibitors (Fig. 3g). Moreover, infection with IAV increased basal and maximal mitochondrial respiratory capacity in a substrate restricted condition, whereas lipase inhibitors suppressed IAV-induced the rate of FAO (Supplementary Fig. 11a), confirming the role of FFAs as an important energy source, particularly at the late stage of virus replication. It is also possible that FFAs contribute to SARS-CoV-2 and IAV replication via the lipidation of viral proteins, since S-palmitoylation or S-fatty-acylation, post-translational protein modifications, are critical for viral membrane fusion, assembly, budding, and virulence.^21,22^ Notably, palmitoylation of the SARS-CoV-2 spike (S) protein and of IAV HA and M2 proteins was significantly inhibited by the treatment of infected cells with atglistatin or CAY10499 (Fig. 3h and Supplementary Fig. 11b). Taken together, these results demonstrated that the pharmacological inhibition of lipases blocks LD lipolysis, thereby inhibiting the use of FFAs as the source of viral protein palmitoylation and energy production via oxidative phosphorylation and possibly inhibiting viral replication compartment formation.^4–7^

### Broad-spectrum safety and antiviral activity in vitro

**Fig. 4:**
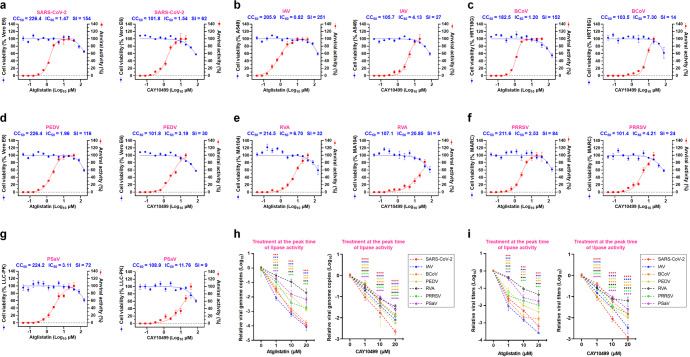
In vitro broad-spectrum safety and antiviral effect of lipase inhibitors. **a**–**g** Dose–response curves for half-maximal inhibitory concentration (IC_50_) and half-maximal cytotoxicity concentration (CC_50_) and selective index (SI) of atglistatin and CAY10499 against seven target RNA viruses. The viral yield in the cell supernatant was quantified by cell-culture immunofluorescence assay. Cytotoxicity of atglistatin and CAY10499 to each cell line was measured by MTT assay. **h**, **i** Effect of atglistatin and CAY10499 on reduction in viral genome copy numbers and viral infectivity titers for seven different RNA viruses in infected cells when treated just prior to the peak of lipase activity. Treatment times are shown in Supplementary Table 4. All data in the graphs are presented as arithmetic means ± SD from four independent experiments. One-way analysis of variance with Tukey’s correction for multiple comparisons

We next carried out to measure in vitro broad-spectrum safety and antiviral effect of lipase inhibitors by measuring half-maximal inhibitory concentration (IC_50_), half-maximal cytotoxic concentration (CC_50_), and selectivity index (SI). Notably, atglistatin and CAY10499 suppressed replication of seven target RNA viruses at even low-micromolar concentrations, showing IC_50_ from 0.82 ± 0.08 µM of atglistatin for IAV to 6.70 ± 0.49 µM of atglistatin for RVA, and 1.54 ± 0.18 µM of CAY10499 for SARS-CoV-2 to 20.85 ± 5.06 µM of CAY10499 for RVA (Fig. 4a–g and Supplementary Table 2). Moreover, these inhibitors showed relatively very low cytotoxicity to seven cell lines originating from humans, monkeys, dogs, and pigs and were used for measuring IC_50_ of seven target RNA viruses, resulting in a very high SI, for example, 251 and 154 SI of atglistatin against IAV and SARS-CoV-2, respectively (Fig. 4a–g and Supplementary Table 2). We next examined whether treatment with non-cytotoxic concentrations (1–20 µM) of atglistatin and CAY10499 (much lower than the CC_50_; Supplementary Table 3) at different treatment times (Supplementary Table 4) exerts broad-spectrum antiviral effects on the seven target RNA viruses in vitro. Treatment of target virus-infected cells with atglistatin or CAY10499 just prior to the peak of lipase activity resulted in a greater reduction in viral genome replication and infectious progeny production of all target RNA viruses in a dose-dependent manner (Fig. 4h, i) relative to virus-infected cells treated with atglistatin or CAY10499 immediately after virus absorption (Supplementary Fig. 12). These data suggested that targeting LD lipolysis shows a therapeutic potential as a broad-spectrum antiviral drug candidate.

### In vivo antiviral effects

To evaluate the in vivo toxicity of atglistatin and CAY10499,^14^ we treated mice and hamsters with intraperitoneal injections at the maximally explored dose of atglistatin (10 mg kg^−1^ d^−1^ for mice and 80 mg kg^−1^ d^−1^ for hamsters) and CAY10499 (10 mg kg^−1^ d^−1^ for mice). As reported previously,^23^ we found that atglistatin and CAY10499 moved through the blood and then accumulated in the lungs of mice and hamsters (Supplementary Fig. 13) and did not induce any overt signs of toxicity, including loss of body weight (Supplementary Fig. 14). In addition, there were no gross and histopathological changes were observed in the major organs (liver, lung, kidney, heart, and spleen) in atglistatin- or CAY10499-treated mice (0.1, 1, or 10 mg kg^−1^ d^−1^ twice daily (Bid) with 12 h interval for 4 consecutive days) (Supplementary Fig. 15) and atglistatin-treated hamsters (20, 40, or 80 mg kg^−1^ d^−1^ Bid with 12 h interval for 4.5 consecutive days) (Supplementary Fig. 16) compared to vehicle-treated control groups.

**Fig. 5:**
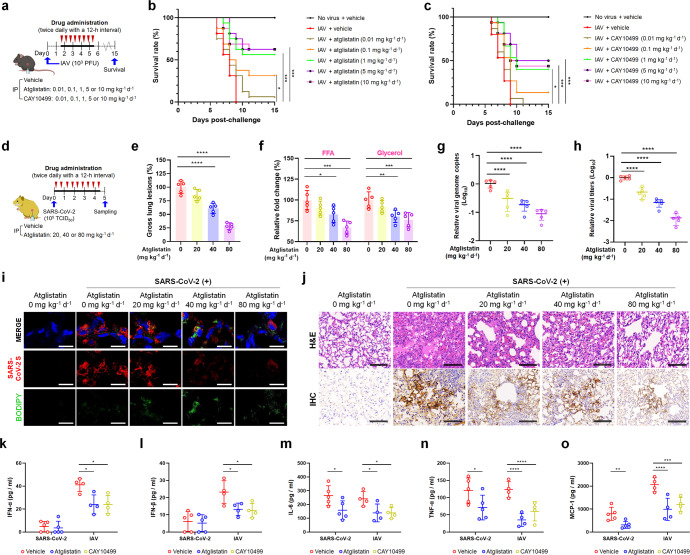
In vivo antiviral and anti-proinflammatory cytokine effects of lipase inhibitors against influenza A virus (IAV) and SARS-CoV-2. **a** Scheme of chemical administration twice daily for 4 consecutive days after challenge with 10^3^ PFU of mouse-adapted IAV PR8 strain to mice (*n* = 16). **b**, **c** Survival rates (expressed as percentages) of IAV-challenged mice by treatment with either atglistatin (**b**) or CAY10499 (**c**). **d** Scheme of chemical administration twice daily for 4.5 consecutive days after challenge with 10^5^ TCID_50_ of SARS-CoV-2 KCDC03 strain to Syrian hamsters (*n* = 5). **e** Reduction in SARS-CoV-2-induced gross lung lesions in hamsters by treatment with atglistatin. **f** In vivo inhibition of FFA and glycerol release from LDs in lungs sampled from SARS-CoV-2-challenged hamsters (*n* = 5) by treatment with atglistatin. **g**, **h** Reduction in SARS-CoV-2 genome copy number and infectious progeny production in lungs sampled from SARS-CoV-2-challenged hamsters (*n* = 5) by treatment with atglistatin. **i** Representative images of LDs and virus replication. Retention of LDs (green, lower panels) and inhibition of SARS-CoV-2 replication (red, middle panels) in alveolar epithelial cells of lung tissues sampled from SARS-CoV-2-challenged hamsters by treatment with atglistatin. **j** Representative images of histological lung lesions. Amelioration of histological lung lesions (upper panels) and inhibition of SARS-CoV-2 replication (lower panels) by treatment with atglistatin. **k**–**o** The graphical representation of the IFN-α, IFN-β, IL-6, TNF-α, and MCP-1 levels in bronchoalveolar lavage fluids (BALFs) sampled from SARS-CoV-2-challenged hamsters or IAV-challenged mice, which were vehicle-treated or treated with atglistatin or CAY10499. Each cytokine in BALFs was determined by ELISA assay as described in Supplementary Information. Results are presented as arithmetic means ± SD. *P < 0.05; **P < 0.01; ***P < 0.001, ****P < 0.0001, one-way analysis of variance with Tukey’s correction for multiple comparisons. Scale bars = 30 µm for panel i and 200 µm for panel **j**

We next evaluated the in vivo antiviral effects of the atglistatin and CAY10499. All the IAV-challenged, vehicle-treated mice in the control group died within 9 dpi, whereas the administration of inhibitors to IAV-challenged mice significantly improved survival rates in a dose-dependent manner, with a 63% survival rate after treatment with atglistatin at 5 and 10 mg kg^−1^ d^−1^ (Fig. 5a–c). Moreover, the administration of atglistatin at different concentrations to SARS-CoV-2-infected Syrian hamsters improved gross lung lesions in a dose-dependent manner, showing a 72% reduction in lung lesions at 80 mg kg^−1^ d^−1^ compared to lesions in the virus-challenged, vehicle-treated control group (Fig. 5d, e). In parallel, the treatment of IAV-challenged mice and SARS-CoV-2-challenged hamsters with these inhibitors resulted in a significant recovery of body weight and fewer clinical manifestations of disease compared to those of the virus-inoculated, vehicle-treated control groups (Supplementary Fig. 17). Consistent with in vitro data, FFA and glycerol levels were significantly reduced in the lung tissues of virus-challenged and drug-treated animals (Fig. 5f and Supplementary Fig. 18a, b), leading to the suppression of viral genome replication and infectious progeny production (Fig. 5g, h and Supplementary Fig. 18c, d). The interruption of virus-induced LD lipolysis resulted in the cytoplasmic retention of LDs (Fig. 5i and Supplementary Fig. 18e) and a remarkable reduction of virus-associated histopathological lung lesions and viral replication (Fig. 5j and Supplementary Fig. 18f). In IAV-challenged mice, moreover, treatment with both lipase inhibitors also reduced blood viral load compared to the mock-treated control groups, consistent with an inhibitory effect on the spread of IAV through viremia (Supplementary Fig. 18g). Interestingly, inhibition of LD lipolysis by treatment of IAV-challenged mice with both lipase inhibitors significantly reduced the levels of activated proinflammatory cytokines including the IFN-α, IFN-β, TNF-α, IL-6, and MCP-1 in the lungs, compared to the vehicle-treated control (Fig. 5k–o and Supplementary Fig. 19). Treatment of SARS-CoV-2-challenged hamsters with atglistatin reduced levels of TNF-α, IL-6, and MCP-1 but had no effect on IFN-α and IFN-β production due to the role of SARS-CoV-2 proteins as antagonists to types I and III interferons (Fig. 5k–o and Supplementary Fig. 19).^18,24^ Taken together, our data suggested that lipase inhibitors have in vivo antiviral and anti-inflammatory cytokine effects on the lung lesions of SARS-CoV-2- and IAV-challenged animals via the blockade of virus-induced LD lipolysis.

### Antiviral synergy with oseltamivir or remdesivir

**Fig. 6:**
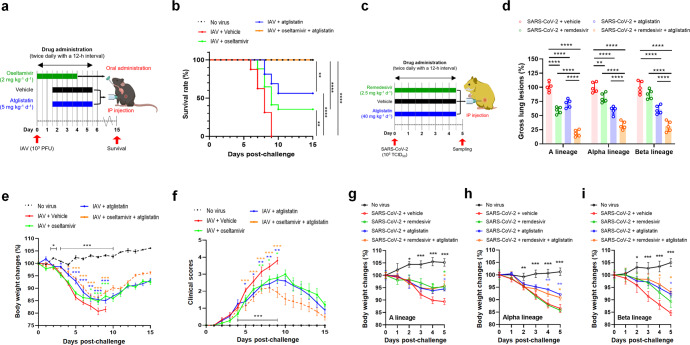
Synergistic in vivo antiviral effects by combination therapy. **a** Scheme of chemical administration of atglistatin and oseltamivir, either individually or in combination, twice daily for 4 consecutive days after challenge with 10^3^ PFU of mouse-adapted IAV PR8 strain to mice (*n* = 16). **b** Survival rates (expressed as percentages) of IAV-challenged mice after treatment. **c** Scheme of chemical administration of atglistatin and remdesivir, either individually or in combination, twice daily for 4.5 consecutive days after challenge with 10^5^ TCID_50_ of SARS-CoV-2 KCDC03 (closely related to early Chines strains), KDCA51463 (Alpha lineage with British variants), and KDCA55905 (Beta lineage with South African variants) to Syrian hamsters (*n* = 5). **d** Reduction in gross lung lesions in Syrian hamsters challenged with each strain by combination therapy with atglistatin and remdesivir. **e** Effect of treatments on the recovery of body weight in each group. **f** Effect of treatments on the reduction in clinical signs in each group. **g**–**i** Effect of combination therapy of atglistatin and remdesivir on the recovery of body weight in Syrian hamsters (*n* = 5) challenged with SARS-CoV-2 KCDC03 (**g**), KDCA51463 (**h**), and KDCA55905 (**i**). Results are presented as arithmetic means ± SD. *P < 0.05; **P < 0.01; ***P < 0.001, ****P < 0.0001, one-way analysis of variance with Tukey’s correction for multiple comparisons

We further evaluated the potential synergistic antiviral effects of ATGL-targeting atglistatin with either IAV neuraminidase-targeting oseltamivir or SARS-CoV-2 RNA-dependent RNA polymerase (RdRp)-targeting remdesivir. The treatment of mice with 10 mg kg^−1^ d^−1^ atglistatin and 2 mg kg^−1^ d^−1^ oseltamivir and the treatment of hamsters with 80 mg kg^−1^ d^−1^ atglistatin and 2.5 mg kg^−1^ d^−1^ remdesivir, either singly or in combination exhibited no toxicity, including no detectable body weight loss (Supplementary Fig. 20). Notably, combination therapy of 5 mg kg^−1^ d^−1^ atglistatin and 2 mg kg^−1^ d^−1^ oseltamivir conferred 100% survival in IAV-infected mice (Fig. 6a, b). Furthermore, combination therapy of 40 mg kg^−1^ d^−1^ atglistatin and 2.5 mg kg^−1^ d^−1^ remdesivir significantly reduced gross lung lesions in hamsters challenged with different strains of SARS-CoV-2. Specifically, KCDC03 (A lineage closely related to early original Chinese strains^25^), KDCA51463 (Alpha lineage in British variants^25^) and KDCA55905 (Beta lineage in South African variants^25^) strain-exposed hamsters treated with a combination therapy showed lung lesions reduced by 82%, 67%, and 69%, respectively (Fig. 6c, d). In parallel, experimental animals administered the combination therapy exhibited better recovery of body weight (both IAV and SARS-CoV-2) and resulted in fewer clinical manifestations (IAV) relative to those of the virus-challenged animals administered the same concentration of either atglistatin, oseltamivir, or remdesivir alone (Fig. 6e–i). Our results demonstrated that combination of atglistatin with virus-targeting drugs, oseltamivir or remdesivir, had better in vivo antiviral effects than those of the individual treatments in all cases.

## Discussion

Attractive targets for host cell-controlling broad-spectrum antivirals are either a common host factor or components of the machinery essential for completing the life cycle of various viruses.^3^ Viruses exploit host lipidomic reprogramming to facilitate viral entry, replication, assembly, or secretion.^4–7^ Previous studies have shown that several RNA viruses enhance LD formation during the early response to viral infection and that blocking lipid biosynthesis inhibits viral replication.^8–11^ Conversely, here we demonstrated that LD catabolism via ATGL and HSL from the mid-stage of infection onwards is a key common feature in the replication of seven high-mutation rate RNA viruses, including SARS-CoV-2 and IAV. Moreover, we demonstrated that inhibitors of cellular lipases have broad-spectrum antiviral potential in vitro and in vivo.

IAV and SARS-CoV-2 damage infected cells and ultimately kill host cells by apoptosis and necroptosis.^16,19,20,26,27^ In these bad milieus, transport of nutrients through dysregulated plasma and/or organelle membranes is restricted or ceased. Thus, FFAs stored in cytoplasmic LDs could be used as viral morphogenesis and energy sources at the late stage of IAV and SARS-CoV-2 infection. In fact, our results showed that both apoptotic and necroptotic cells due to either IAV or SARS-CoV-2 infection were high in the late stage of both viral infections when the breakdown of LDs occurred actively in the virus-infected cells. In parallel, significant reductions in cellular glucose uptake were also found at the late stage of SARS-CoV-2 and IAV infections, indicating dysfunction of cellular machinery including membrane transporter systems, particularly at the late stage of infection. The release of FFAs from intracellular stored LDs is also likely critical to provide substrates for energy production via FAO in mitochondria and for the palmitoylation of the key SARS-CoV-2 S protein and IAV HA and M2 proteins, which are necessary for the late stages of virus replication and maturation.^6–11,21,22^ Indeed, we demonstrated that blocking LD lipolysis by the treatment with lipase inhibitors significantly reduced FAO and viral protein palmitoylation. Thus, our data suggested that LDs induced and accumulated at the early stage of RNA virus infection are used as the source of energy, viral palmitoylation, and possibly viral replication compartment at the late stage of both viral infections.

Our LC-MS data showed that treatment of IAV-infected cells with lipase inhibitors (atglistatin and CAY10499) significantly reduced the levels of two FFAs, PA and OA, compared to their abundance in untreated control groups. Supplementation of saturated PA and unsaturated OA and LA in the condition of FFA deficiency has been reported to restore the replication of not only RNA viruses including not only SARS-CoV-2, IAV, MERS-CoV, and enterovirus but also DNA viruses such as adenovirus.^11,15^ In agreement with previous studies,^11,15^ supplementation of FFAs during FFA deficiency by lipase inhibitors was found to rescue the replication of SARS-CoV-2 and IAV. The addition of unsaturated FFAs such as LA and OA at the time of virus inoculation is known to inhibit pathogenic human β-coronaviruses through their binding to FFA binding pocket in the spike protein, which blocks virus entry.^28–31^ This is contrast with our results where supplementation of FFAs in the lipase inhibitor-induced FFA deficiency condition during the active virus replication stage resulted in increased virus replication through providing an energy factory and increasing viral morphogenesis.^4–7^ In addition, supplementation of FFAs in the above FFA deficiency condition could promote the replication of SARS-CoV-2 and IAV through its feedback inhibition of cytosolic phospholipase A2.^31–33^

Proinflammatory cytokines are essential in the host response that inhibits virus replication, but local and systemic hyper-induction, also known as a cytokine storm, is a fetal driving force for critical infection or increased mortality in patients infected with SARS-CoV, MERS-CoV, SARS-CoV-2, and IAV. In fact, this might be more prominent than the direct inhibitory effects on the virus life cycle.^34–37^ In addition to in vitro effects, interestingly, SARS-CoV-2- and IAV-induced activation of proinflammatory cytokines in the lung lesions in our study was significantly reduced by the treatment of virus-challenged animals with lipase inhibitors, which coincided with a significant reduction in the level of FFAs. Since cytokine storm could be induced by an FFA-derived eicosanoid storm after induction of an ER stress response directly via the virus itself or indirectly via debris from virus-induced cell death,^7,38^ our data suggested that blockade of FFA release from virus-induced LDs and/or decrease in the amount of debris from virus-induced cell death after treatment with lipase inhibitors could reduce the eicosanoid storm/cytokine storm pathway. Of note, an inhibitor-induced FFA deprivation was found to decrease viral genome copy number and protein expression of SARS-CoV-2- and IAV in vitro and in vivo through interruption of energy production, viral morphogenesis, and possibly viral replication compartments. Thus, a reduction in the interaction of viral components as a pathogen-associated molecular pattern (PAMP) with pattern-recognition receptors (PRRs) could decrease cytokine production.^34–38^ Taken together, we hypothesize that reduction in FFA release from LD by lipase inhibitors could mitigate the SARS-CoV-2- and IAV-induced cytokine storm through blocking a FFA-driving eicosanoid storm and virus replication-associated interaction of PRRs and PAMP.^34–38^ In addition, lipase inhibitor-induced reduction in the cytokine levels could be beneficial in reducing critical infection or mortality due to cytokine storming in the virus-challenged experimental animals.^34–36^

Interestingly, greater efficacy of atglistatin and CAY10499 was observed when applied to the virus-infected cells just prior to the peak time of lipase activity. Since liquid-liquid phase separation of viroplasms interacted with LDs has been reported to alter the function of various host proteins, it may interfere with the effectiveness of atglistatin and CAY10499 to lipases and their associated proteins on the LDs.^39^ Virus-induced LDs are known to enhance the early antiviral innate immune response and inhibit viral replication,^7,40^ suggesting stabilization of LDs by treatment with a lipolysis inhibitor and then blockade of the antiviral response. The potential reasons why atglistatin and CAY10499 are less effective at suppressing various RNA viruses when treated in the early stage of infections may be the result of instability, inactivation or an inability to effectively access the LDs at the critical time remain unclear; however, this is an active area of investigation.

Nevertheless, in SARS-CoV-2-infected hamsters and IAV-infected mice, early administration of lipolysis inhibitors from 1 day post-inoculation resulted in a significant reduction in mortality and alleviation of lung pathology and virus replication. In virus-infected animals, different steps of the virus life cycle should occur in each individual cell infected at different times, rendering that all steps of the viral life cycle simultaneously occur in infected animals. Therefore, the administration of lipolysis inhibitors even from the early infection could block specific steps of the virus life cycle requiring FFAs generated from LDs by lipolysis. It could be supported by notable in vivo findings in this study; significant low levels of FFAs and glycerol and retention of LDs were observed in virus-infected animals after treatment with lipolysis inhibitors compared to vehicle-treated controls. These in vivo data also supported that blocking FFA production from LDs through lipase inhibition limits virus replication and maturation via interrupting both viral protein palmitoylation and energy production and possibly biogenesis of viral replication compartments for viral replication.^6–11^

Toxicity is a limiting factor in the therapeutic application of many drugs with known antiviral activity.^41–43^ In this study, the non-selective lipase inhibitors, atglistatin and CAY10499, potently blocked SARS-CoV-2 infection at low-micromolar concentrations and showed a relatively high selectivity index in comparison to FDA-approved chemicals,^43^ suggesting its safety and effectiveness as a therapeutic agent. Moreover, the combination of the lipase inhibitor atglistatin with IAV NA-targeting oseltamivir or SARS-CoV-2 RdRp-targeting remdesivir, even at lower concentrations, effectively protected mice from lethal IAV infection and much improved the therapeutic effects on SARS-CoV-2-induced lung lesions in a hamster model compared to those for the individual drugs.^41^ Future clinical studies of a single or combined antiviral therapy with LD-associated lipase inhibitors and drugs targeting viral proteins are warranted for the treatment of patients with severe acute viral infections, such as COVID-19.^42^

## Materials and methods

### Cells, viruses, and culture conditions

A549, Caco-2, MARC-145, and HRT-18G cells in DMEM, Vero E6 and LLC-PK cells in EMEM, and MA104 cells in *α*-MEM, which were all supplemented with 10% fetal bovine serum, 100 U/mL penicillin, and 100 μg/mL streptomycin, were cultured at 37 °C in a humidified atmosphere of 5% CO_2_. The IAV A/Puerto Rico/8/1934 (H1N1) (PR8) strain, three strains of SARS-CoV-2 (KCDC03, KDCA51463, and KDCA55905), BCoV KWD20 strain, PEDV QIAP1401 strain, PRRSV LYM strain, RVA NCDV strain, and PSaV Cowden strain were used in this study. Detailed procedures for cell and virus culture and virus titration are described in Supplementary Information.

### Reagents, antibodies, siRNAs, and kits

The reagents, antibodies, siRNAs, and kits used in this study are described in Supplementary Information.

### Determination of 50% cytotoxicity concentration (CC_50_)

The cytotoxic effects of the chemicals and their solvents were tested by the MTT assay,^44^ and its procedure is described in Supplementary Information.

### Determination of the cAMP level

The difference in the level of cAMP between the untreated and the treated cells after infection with IAV or SARS-CoV-2 was determined as previously reported,^45^ and its procedure is described in Supplementary Information.

### Treatment of cells with inhibitory chemicals and FFAs

Cells were grown in 6- or 12-well plates or 8-well chamber slides to the desired confluency and then washed twice with phosphate-buffered saline (PBS, pH 7.4). They were then mock-inoculated with medium only or virus-inoculated with various multiplicities of infection (MOI): MOI of 0.01 or 1 FFU/cell for IAV, and MOI of 0.01 or 0.1 FFU/cell for SARS-CoV-2, BCoV, PEDV, PRRSV, and PSaV. Thereafter, each inoculum was absorbed for 1 h, and the cells were washed twice with PBS (pH 7.4). As shown in Supplementary Table 4, further treatments and incubations varied dependent on the virus species used. Cells treated with PKA inhibitor H89 (5 μM or 50 μM concentration) or vehicle-treated immediately after absorption of IAV at an MOI of 1 FFU or SARS-CoV-2 at an MOI of 0.1 FFU were incubated for 24 and 36 h, respectively, and each cell lysate was used for the detection of HSL and pHSL by western blot analysis as described below. Cells treated with lipase inhibitors at the median incubation time of IAV or SARS-CoV-2 as described in the above condition were supplemented with LA, OA, or PA at 100 µM concentration and left incubation for further median incubation time. The cells were used to assess levels of intracellular TAGs, cholesterol, FFAs, glycerol, FAO, proinflammatory cytokines as well as viral genome copy numbers and virus titers as described below and in Supplementary Information.

### Determination of FFAs

The difference in saturated and unsaturated FFAs between the untreated and the treated cells after infection with IAV or SARS-CoV-2 was determined by GC-FID and LC-MS as previously reported,^11^ and detailed procedures are described in Supplementary Information.

### Transfection of siRNA

A549 cells grown in 12-well culture plates or 8-well chamber slides with 70 and 80% confluency were transfected with siRNAs against ATGL (1 and 10 nmol) and/or HSL (1 and 10 nmol) using Lipofectamine 3000 (Thermo Scientific) as described elsewhere.^46^ To optimize the efficient knockdown of target proteins, a second transfection into cells was performed 24 h after the first transfection, and the cells were incubated for a further 24 h. As a negative control, scrambled siRNA (10 nmol) was transfected in cells as described above.

### Glucose uptake assay

Glucose uptake into IAV-infected A549 or SARS-CoV-2-infected Vero E6 cells was evaluated by measurement of the fluorescent glucose analog 2-[N-(7-nitrobenz-2-oxa-1,3-diazol-4-yl) amino]-2-deoxy-D-glucose (2-NBDG) from Abcam according to the manufacturer’s method as described in Supplementary Information.

### Flow cytometry

To determine the proportion of apoptosis marker (TUNEL)-positive and necroptosis marker (pMLKL)-positive cells and to quantify FITC-conjugated glucose in IAV-infected A549 and SARS-CoV-2-infected Vero E6 cells, flow cytometry assay was performed as described elsewhere,^11^ and its procedure is described in Supplementary Information.

### FAO assays

The difference in the level of fatty acid oxidation and oxidation consumption rates between the untreated and the treated cells after infection with IAV or SARS-CoV-2 was determined as previously reported,^47^ and detailed procedures are described in Supplementary Information.

### Ethics statement

In the animal experiments, all procedures were performed in accordance with the institutional animal care and use committees’ requirements at Chonnam National University (CNU IACUC‐YB‐2018‐41) and Korea Research Institute of Bioscience & Biotechnology (KRIBB-AEC-21203, KRIBB-IBC-20210206). The care and handling of animals complied with all of the current international laws and policies (NIH Guide for the Care and Use of Laboratory Animals, NIH Publication No. 85‐23, 1985, revised 1996). All experiments were conducted in a manner that minimized the number of animals used, and the suffering caused.

### Experimental animals

Information on the species and breeding of experimental animals used in this study is described in Supplementary Information.

### Determination of median lethal dose (LD_50_) of mouse-adapted PR8 strain

The procedure for determining the LD_50_ of the mouse-adapted PR8 strain in 8-week-old mice is described in Supplementary Information.

### Lung and blood distribution of atglistatin and CAY10499

The distribution of atglistatin and CAY10499 in the lung and blood samples of experimental animals was determined by LC-MS as described previously with slight modification,^23^ and detailed protocols are described in Supplementary Information.

### Measurement of SARS-CoV-2-induced gross lung lesion

In the Syrian hamster model, SARS-CoV-2 infection causes severe acute edema, congestion, hemorrhage, and infiltration of mononuclear cells in the lung tissues.^48^ After taking photographs of the dorsal and ventral lung surfaces, the size of lung lesions was measured using NIH ImageJ software. The therapeutic effect on the size of the gross lung lesions by treatment with atglistatin and remdesivir either individually or in combination in the hamster model was calculated using the following formula, [(surface lung lesion size in virus-challenged, chemical treated group)/(surface lung lesion size in the virus-challenged, mock-treated group)] × 100%.

### In vivo antiviral activity, lipid metabolism, and pathogenicity

The procedure to determine the antiviral effects of atglistatin and CAY10499 either singly or in combination with oseltamivir or remdesivir against IAV infection in the mouse model and the hamster model is described in Supplementary Information.

### In vivo organ-specific toxicity

The procedure for determining organ-specific toxicity of atglistatin and CAY10499 in experimental animals is described in Supplementary Information.

### Plaque assay

Plaque assay for determining the viral titer of IAV is described in Supplementary Information.

### Median tissue culture infectious dose (TCID_50_) assay

TCID_50_ assay for determining the viral titer of SARS-CoV-2 is described in Supplementary Information.^46,49^

### Immunofluorescence assay (IFA)

The dynamics of LD formation, apoptotic and necroptotic cells, and virus infectivity in cultured cells or lung tissues were determined by IFAs.^46^ The IFA was also used to identify cells transfected with siRNA or scrambled siRNA. The procedure is described in Supplementary Information.

### Western blot analysis

Western blot analysis was used to determine the expression levels of target cellular and viral proteins in the culture cells or lung tissues as described elsewhere.^46^ The procedure is described in Supplementary Information.

### Triacylglyceride (TAG) colorimetric assay

Intracellular TAGs were measured using a triglyceride colorimetric kit (Cayman Chemicals) according to the manufacturer’s recommendations as described in Supplementary Information.

### Cholesterol colorimetric assay

The level of intracellular cholesterols was measured using a cholesterol colorimetric assay kit (Abcam) according to the manufacturer’s recommendations as described in Supplementary Information.

### Free fatty acid quantification

The amount of intracellular free fatty acid was determined using a quantification kit from BioVision according to the manufacturer’s recommendations as described in Supplementary Information.

### Free glycerol quantification

The levels of intracellular free glycerol in the different cultured cells and lung samples under different conditions were determined using a quantification kit from BioVision according to the manufacturer’s recommendations as described in Supplementary Information.

### Quantitative real-time PCR

The target viral RNAs and host mRNAs in both cultured cells and lungs and blood samples from experimental mice or hamsters were quantified using real-time PCR as described previously with slight modifications as described in Supplementary Information.

### Palmitoylation assay

To determine the influence of the lipase inhibitors CAY10499 on palmitoylation of SARS-CoV-2 S protein or IAV HA and M2 proteins in virus-infected cells, the palmitoylation assay was performed by CAPTUREomeTM S-palmitoylated protein kit (Badrilla) as described in Supplementary Information.

### Histopathology and immunohistochemistry (IHC)

Histopathological changes and expression levels of IAV or SARS-CoV-2 proteins in lungs from the experimental mice or hamsters in each experimental group were determined as described elsewhere.^48,50^ The procedures are described in Supplementary Information.

### Statistical analyses and software

Statistical analyses were performed on triplicate experiments by One-Way ANOVA using GraphPad Prism software version 8.4.2 (GraphPad Software Inc., La Jolla, CA, USA). *P* values of less than 0.05 were considered statistically significant. Figures were generated using Adobe Photoshop CS6 and Prism 8 version 4.2. NIH ImageJ software (version 1.48, Java 1.8.0) was used for image processing for pathological analysis.

### Illustrations

Illustrations of animals in Figs. 5 and 6 were created with BioRender software (https://biorender.com/).

## Supplementary information

Supplementary Materials

Therapeutic strategy targeting host lipolysis limits infection by SARS-CoV-2 and influenza A virus

## Data Availability

All data are provided in the manuscript and supplement.
