# Supplementary material for: Therapeutic strategy targeting host lipolysis limits infection by SARS-CoV-2 and influenza A virus
Source: Signal Transduct Target Ther. 2022 Oct 17;7:367. doi: 10.1038/s41392-022-01223-4 (PMC9575645; doi:10.1038/s41392-022-01223-4)
Supplement: Supplementary file 1 — Supplementary Materials [file 41392_2022_1223_MOESM1_ESM.docx]

**Supplementary Materials for**

**Therapeutic strategy targeting host lipolysis limits infection by SARS-CoV-2 and influenza A virus**

Yeong-Bin Baek^1^*, Hyung-Jun Kwon^2^*, Muhammad Sharif^1^, Jeongah Lim^3^, In-Chul Lee^2^, Young-Bae Ryu^2^, Jae-In Lee^2^, Ji-Sun Kim^4^, Young-Seung Lee^5^, Dong-Hoon Kim^6^, Sang-Ik Park^1^, Don-Kyu Kim^7^, Jeong-Sun Kim^3^, Hyon E Choy^8^, Sunwoo Lee^3^, Hueng-Sik Choi^9^, Timothy F. Osborne^10^, Tae-Il Jeon^5†^, Kyoung-Oh Cho^1†^

Correspondence to: Tae-Il Jeon (tjeon@jnu.ac.kr); Kyoung-Oh Cho (choko@jun.ac.kr).

**This PDF file includes:**

Supplementary Materials and Methods

Tables S1 to S22

Figures. S1 to S20

References

Original Films of Western Blots

**Supplementary Materials and Methods**

**Cells, viruses, and infection**

African green monkey kidney epithelial Vero E6, human lung carcinoma A549, canine kidney epithelial MDCK, human colorectal adenocarcinoma Caco-2, African green monkey kidney epithelial MA104, African green monkey kidney epithelial MARC-145, porcine kidney epithelial LLC-PK, and human colorectal adenocarcinoma HRT-18G cells were purchased from the American Type Culture Collection (ATCC, Manassas, VA, USA). A549, MDCK, Caco-2, MARC-145, and HRT-18G cells were cultured in Dulbecco’s modified Eagle’s medium (DMEM), Vero E6 and LLC-PK cells in Eagle's Minimum Essential Medium (EMEM), and MA104 cells in alpha minimal essential medium (*α*-MEM), which were all supplemented with 10% fetal bovine serum (FBS), 100 U/mL penicillin, and 100 μg/mL streptomycin.

The IAV A/Puerto Rico/8/1934 (H1N1) (PR8) strain was purchased from the ATCC and propagated in MDCK cells supplemented with 1 μg/mL N-tosyl-L-phenylalanine chloromethyl ketone (TPCK)-treated trypsin (Sigma Aldrich, St. Louis, MO, USA) as described previously.^1^ Three strains of SARS-CoV-2, KCDC03 (isolated from Korean COVID-19 patient in 2020 and belonging to the A lineage of early Chinese strains), KDCA51463 (isolated from Korean COVID-19 patient in 2021 and belonging to the Alpha lineage of British variants), and KDCA55905 (isolated from Korean COVID-19 patient in 2021 and belonging to the Beta lineage of South African variants),^2^ were kindly provided by the Korea Disease Control and Prevention Agency (Cheongju, South Korea) and propagated in Vero E6 cells.^3^ Bovine coronavirus (BCoV) KWD20 strain was isolated from a diarrhea fecal sample of an adult cow and propagated in human rectal tumor HRT-18G cells.^4^ In brief, monolayers of HRT-18G cells grown in 6-well plates were washed with EMEM and then inoculated with 0.2 μm filtered fluid obtained from fecal samples, shown as positive for BCoV by RT-PCR. After 1 h adsorption with occasional rocking, EMAM containing porcine pancreatin (5 μg/ml) was added. Isolated BCoV was cloned by liquid-limiting dilution, and the highest dilution of the virus that caused any CPE was passaged an additional three times in the HRT-18G cells. The isolated KWD20 strain was confirmed by IFA and RT-PCR. Porcine epidemic diarrhea virus (PEDV) strain QIAP1401 and porcine reproductive and respiratory syndrome virus (PRRSV) North American type strain LMY were kindly provided by the Animal and Plant Quarantine Agency (Gimcheon, South Korea) and propagated in Vero E6 and MARC-145 cells, respectively.^5,6^ The bovine species A rotavirus (RVA) NCDV (G6P6[1]) strain from the ATCC was preactivated with 10 μg/mL porcine trypsin (Cat. No. 27250–018, Gibco, Fort Worth, TX, USA), and propagated in MA104 cells as described previously.^7^ The porcine sapovirus (PSaV) Cowden strain was generated from the full-length infectious clone pCV4A and propagated in LLC-PK cells in the presence of 200 μM glycochenodeoxycholic acid (GCDCA).^8^ The cells and viruses used in this study are listed in Tables S5 to S7.

IAV titer was determined by plaque assay or cell culture immunofluorescence (CCIF) assay and expressed as plaque forming units per milliliter (PFU/mL) or fluorescence focus units per milliliter (FFU/mL) as described below. Titers of SARS-CoV-2 were measured by 50% tissue infectious dose (TCID_50_) or CCIF assay using a monoclonal antibody (Mab) specific for SARS-CoV-2 nucleocapsid (N) protein and expressed as TCID_50_/ml and FFU/mL as described below. BCoV, PEDV, RVA, PRRSV, and PSaV were determined by CCIF assay using Mabs specific for BCoV spike (S) protein, PEDV nucleoprotein (N), RVA VP6 protein, PRRSV matrix (M) protein, or PSaV VPg protein, and expressed as FFU/mL.

**Reagents, antibodies, siRNAs, and kits**

Atglistatin purchased from MedChemExpress (Monmouth Junction, New Jersey), CAY10499 from Cayman Chemicals (Ann Arbor, Michigan), and H89 dihydrochloride from Sigma Aldrich were dissolved in DMSO to prepare stock solutions for *in vitro* experiments. TPCK-treated trypsin, oseltamivir, porcine pancreatic trypsin, GCDCA, 3-(4,5-dimethylthiazol-2-yl)-2,5-diphenyl tetrazolium bromide (MTT), triton X-100, and Bodipy (493/503) purchased from Sigma Aldrich, pancreatin and crystalized trypsin from Gibco (Fort Worth, TX), Accutase cell detachment solution from BD biosciences (Franklin Lakes, NJ), and palmitic, oleic, and linoleic acids from Sigma Aldrich were dissolved in PBS (pH 7.4) to prepare stock solutions.

For animal experiments, pharmacological chemicals were dissolved as follows; atglistatin and CAY10499 were dissolved in 10% polyethylene glycol 400 (PEG400) in water (v/v) for administration to IAV-infected mice and 50% PEG400 in water (v/v) for administration to SARS-CoV-2-infected hamsters. Oseltamivir was dissolved in water, whereas remdesivir was dissolved in a mixture of 10% DMSO and 90% corn oil (v/v). Kits used in this study included glucose uptake assay and cholesterol assay kits from Abcam, triglyceride colorimetric assay kit from Cayman Chemicals (Ann Arbor, MI), free fatty acid quantification and colorimetric/fluorometric kits from BioVision (Milpitase, CA), CAPTUREomeTM S-palmitoylated protein kit from Badrilla (Leeds, UK), RNeasy mini kit from Qiagen (Hilden, Germany), and TOPscript cDNA synthesis kit from Enzynomics (Daejeon, South Korea). Bicinchoninic acid (BCA) protein assay kit and Lipofectamine3000 were purchased from Thermo Scientific (Waltham, MA). Mouse monoclonal antibodies (Mabs) against IAV nucleoprotein (NP) and IAV matrix protein 2 (M2) and rabbit polyclonal antibodies (Pabs) against hormone sensitive lipase (HSL) were purchased from Abcam. Rabbit Mabs against phosphorylated protein kinase A (PKA) C for Thr197, rabbit Pabs against PKA C-α, phosphorylated HSL (pHSL) for S563 and S660 were acquired from Cell Signaling (Beverly, Massachusetts, USA), and rabbit Pabs against pHSL for S563 and S660 were acquired from Cell Signaling (Beverly, Massachusetts, USA), and rabbit Pab against SARS-CoV-2 S protein and mouse Mab against SARS-CoV-2 N were from Prosci (San Diego, CA, USA). Mouse Mab against IAV hemagglutinin (H1N1), and mouse Mabs against perilipin 3 (PLIN3) and GAPDH were obtained from Santa Cruz (Dallas, Texas, USA), and β-actin was from Thermo Scientific (Waltham, MA, USA). Goat antiserum against IAV whole virion was purchased from ViroStat (Portland, ME, USA). Mouse Mab against RVA VP6 protein was obtained from Median Diagnostic (Chuncheon, South Korea). Rabbit Pab against PSaV VPg was generated by immunization of a New Zealand White rabbit with purified VPg.^9^ Rabbit Pab against PRRSV M protein and mouse Mab against PEDV N protein (clone; SD6-29) were purchased from Bioss Antibodies (Woburn, MA, USA) and Medgene Labs (Brookings, SD, USA), respectively. Mouse Mab against BCoV S protein (5A4) was purchased from Native Antigen Company (Killington, UK). Horseradish peroxidase (HRP)-conjugated secondary antibodies, including goat anti-rabbit IgG and donkey anti-goat IgG from Cell Signaling, and goat anti-mouse IgG from Santa Cruz were used for western blot and immunohistochemistry (IHC). Alexa Fluor (AF) 594-conjugated donkey anti-rabbit IgG, AF488-conjugated donkey anti-rabbit IgG, AF594-conjugated goat anti-mouse IgG, and AF488-conjugated goat anti-mouse IgG purchased from Thermo Scientific, and DyLight594-conjugated donkey anti-Goat IgG from Bethyl Laboratories (Montgomery, TX, USA) were utilized for immunofluorescence assay. Bodipy 493/503 purchased from Sigma Aldrich was applied to stain neutral lipids in lipid droplets in the cells. Slow-Fade Gold antifade reagent with 4', 6-diamidino-2-phenylindole (DAPI) was obtained from Molecular Probes for nuclear staining. Protein A-agarose and protein G plus-agarose were purchased from Santa Cruz. All of the siRNAs were purchased from GE Healthcare Dharmacon (Lafayette, CO, USA). For plaque assay, ultrapure agarose was purchased from Thermo Scientific. Reagents, antibodies, and kits used in this study are listed in Tables S7 and S8.

**Determination of 50% cytotoxicity concentration (CC_50_)**

The cytotoxic effects of the chemicals and their solvents used were tested by the MTT assay as described previously.^10^ In brief, the chemicals were dissolved in DMSO to a stock concentration of 10 mM, and then freshly serially diluted in serum-free medium before being added to cell culture monolayers. The absorbance was measured using an enzyme-linked immunosorbent assay (ELISA) reader at an optical density (OD) of 570 nm. The percent cell viability was calculated using the following formula, [(OD_sample_˗OD_blank_)/(OD_control_˗OD_blank_)] × 100%. According to the cell cytotoxicity data, dose-response curves were generated for the calculation of CC_50_ using GraphPad Prism software version 8.4.2.

**Quantification of cyclic adenosine monophosphate (cAMP) levels**

To determine the cAMP level in the cells treated with H89 or vehicle after infection with SARS-CoV-2 or IAV, a PKA-specific inhibitor cAMP Direct Immunoassay kit (BioVision) was used as previously reported.^11^ In brief, confluent Vero E6 and A549 cells grown in a 12-well plate were infected with SARS-CoV-2 KCDC03 strain at an MOI of 0.1 and IAV PR8 strain at an MOI of 1, respectively, and incubated for the indicated time points. Collected cells were lysed 0.1 M hydrochloric acid (HCl), and the supernatants were incubated with a cAMP antibody. The amount of cAMP was measured by reading activity of cAMP-HRP conjugate at OD at 450 nm, and subject to normalization after generation of the standard curve with offered Standard cAMP in the kit.

**The preparation of fatty acid methyl esters (FAMEs) from the cells**

Before determining the composition of FFAs in the mock- and IAV-infected A549 cells, the fatty acids were saponified, methylated, and extracted according to the protocol of the MIDI/Hewlett Packard Microbial Identification System.^12^ The freeze-dried cells were dissolved in saponification reagent (45 g sodium hydroxide, 150 ml methanol, and 150 ml distilled water) and boiled for 30 min at 100 °C. The samples were added with methylation reagent (325 ml of 6 N hydrochloric acid and 275 ml methyl alcohol) and heated for 10 min at 80°C. The extraction reagent (hexane:methyl tert-butyl ether with 1:1 ratio) was added to samples, and the lower phase of samples was discarded. Subsequently, the base wash reagent (10.8 g sodium hydroxide dissolved in 900 ml distilled water) was added to the organic phase of the samples. After then, the upper phase of the samples was transferred to a new GC vial.

**Gas chromatography-flame ionization detector (GC-FID) analysis of FAMEs**

To determine the composition of FFAs in the mock- and IAV-infected A549 cells, GC-FID analysis of FMEs was performed on a Shimadzu GC-2010 Plus gas chromatograph equipped with an FID. Supelco 37 Component FAME Mix was used as the reference standard of FAMEs. The 1 *μ*L of the extracts was injected into a DB-23 column (30 m × 250 μm i.d., 0.25 μm film thickness; Agilent Technologies, CA, USA) using an AOC-20i injector in the split mode. The initial GC oven temperature was 50°C. After injection, the GC oven temperature was increased with 25°C/min to 175°C, 4°C/min to 230°C, and held for 5 min at 230°C. Nitrogen was used as a carrier gas. Detection was achieved using FID at 280 °C.

**LC-MS for quantifying palmitic acid and oleic acid**

The amount of palmitic and oleic acids in the untreated and treated cells after infection with IAV was determined by LC-MS. In brief, A549 cells infected with IAV PR8 strain at an MOI of 0.1 were vehicle-treated, or treated with 20 μM atglistatin or CAY10499 at 12 hpi, or treated with 20 μM atglistatin or CAY10499 and then supplemented individually with 100 μM of PA or OA at 12 hpi, and incubated further for 12 h. Afterward, cells were collected for FFA extraction. 700 μL Chloroform : Methanol (2:1) was added to the freeze cell pellets and vortexed for 1 min. The mixture was sonicated for 5 min and re-vortexed for 1 min. The mixture was centrifuged at 13,000 rpm for 8 min, and the lower organic layer was collected and transferred into a micro-tube. Using vacuum concentrator, the extracts were dried. Finally, the extracts were re-dissolved by methanol and centrifuged at 13,000 rpm for 5 min. The supernatants were transferred into LC-MS vials. LC-MS was performed with an Acquity UPLC I-Class Plus system coupled to a Waters XEVO TQ-S (triple quadrupole) MS system (Waters, Milford, MA), equipped with an electrospray source operated in the negative-ion mode. An aliquot of the extracted sample (10 μL) was injected into an Acquity UPLC BEH-C18 reversed-phase column (2.1 X 50 mm column size, 1.7 μm particle size) with two solvents: (A) distilled water (DW) + 0.1% formic acid and (B) acetonitrile. A gradient programmer was used according the following profile: 0-10 min, 30-90% (B); 10-12 min hold on, 90% (B); 12-15 min, 90-30% (B). The flow rate during the experiment was 0.3 mL/min. The ESI-MS data were acquired in negative ion mode and conditions of MS analysis were as follows: capillary voltage, 2.50 kV; cone voltage, 60 V; desolvation gas flow, 550 L/h; cone gas flow, 150 L/h; desolvation temperature, 220°C. The standard compounds as well as extracted samples were analyzed in selective ion monitoring (SIM) mode using the ESI source by monitoring the H^-^ adducts ions. For standard compounds and extracted samples, selected ions scans for each compound were restricted to specific retention time windows (9.95-10.1 min, m/z 255.17, Palmitic acid; 10.05-10.2 min, m/z 281.29, Oleic acid).^13^ Calibration curves were linear with correlation coefficients (R^2^) above 0.9954 (palmitic acid) and 0.9991 (oleic acid) for all analytes.

**Glucose uptake assay**

Glucose uptake into A549 and Vero E6 cells infected with IAV or SARS-CoV-2, respectively, was evaluated by measurement of the fluorescent glucose analog 2-[N-(7-nitrobenz-2-oxa-1,3-diazol-4-yl) amino]-2-deoxy-D-glucose (2-NBDG) from Abcam according to the methods described previously.^14^ Briefly, confluent A549 and Vero E6 cells grown on a Corning® white clear-bottomed 96-well microplate (Sigma-Aldrich) were infected with the IAV PR8 strain at an MOI of 1 FFU/cell or the SARS-CoV-2 KCDC03 strain at an MOI of 0.1 FFU/cell for the indicated times. Afterward, the medium was replaced with Krebs-Ringer phosphate HEPES (KRPH) buffer (pH 7.4) containing 80 μM fluorescent glucose analog 2-NBDG in each well and then incubated for 20 min at 37°C. The medium was replaced with 1x cell-based assay buffer, and then 2-NBDG uptake was measured by the increase in fluorescence intensity at an excitation wavelength of 485 nm and emission wavelength of 535 nm using a fluorometer (FluroMax 2, Horiba, Kyoto, Japan). The level of 2-NBDG uptake was expressed as a fold of relative fluorescence intensity of the mock-inoculated, mock-treated control cells. To visualize intracellular 2-NBDG uptake, cells were cultured in 8-well chamber slides and underwent the same procedure as described above with a modification of treatment volume proportionally based on the seeded cell numbers. After treatment with 2-NBDG, the cells were fixed with 4% buffered paraformaldehyde (PFA) for 10 min, permeabilized by 0.2% Triton-X for 4 min at 20°C and mounted with SlowFade Gold antifade containing 1 × DAPI solution for nuclear staining. Images were taken by confocal microscopy.

**Flow cytometry**

To quantify FITC-conjugated glucose uptake in IAV-infected A549 and SARS-CoV-2-infected Vero E6 cells, a flow cytometry assay was performed as described elsewhere.^13,15,16^ Briefly, mock-infected or virus-infected cells were sequentially harvested at different time points by incubation at 37°C for 5 min with Accutase^TM^ Cell Detachment Solution (BD Biosciences, Franklin Lakes, NJ, USA), washed with PBS and pelleted. Ten thousand single events from resuspended A549 or Vero E6 cells in 500 μL of cold PBS were firstly examined by relative size (Forward Scatter-FSC), granularity, or internal complexity (Side Scatter-SSC). Subsequently, only cells with the appropriate cellular properties were analyzed for fluorescence activity at 488/530 nm (Excitation/Emission). All procedures were performed with the AttuneTM NxT flow cytometer (Thermo Scientifics) and the data from each sample in a linked result were digitized under the same flow cytometric measurement condition using the AttuneTM NxT software v3.1.2.

For evaluating the portion of necroptosis marker (pMLKL)-positive and apoptosis marker (TUNEL)-positive either IAV-infected A549 or SARS-CoV-2-infected Vero E6 cells in a time-dependent manner, cells were collected and prepared as above. In addition, we determined the effects of lipase inhibitors on the change of pMLKL- and TUNEL-positive cell portions, particularly in the supplementation of each individual saturated and unsaturated FFA. Mock- or IAV-infected cells were incubated with 1:200 diluted mouse Mab against IAV M2 protein together with either necroptotic or apoptotic markers at 37°C for 1 h, the antibody against pMLKL or terminal deoxynucleotidyl transferase-mediated uridine 5’-triphosphate-biotin nick end-labeling (TUNEL) reaction mixture (In situ Cell death detection kit, Basel, Switzerland), respectively. After washing twice with PBS, goat AF647-conjugated anti-mouse IgG antibody at a 1:200 dilution as a second antibody for binding to an antibody against IAV M2 protein and goat AF488-conjugated goat anti-rabbit antibody at a 1:200 dilution corresponding to an antibody against pMLKL were applied and incubated at 37°C for 1h. Mock- or SARS-CoV-2-infected cells were incubated with 1:200 diluted mouse Mab against SARS-CoV-2 S protein together with either necroptotic or apoptotic markers at 37°C for 1 h. After washing twice with PBS, goat AF647-conjugated anti-mouse IgG antibody at a 1:200 dilution as a second antibody for binding to an antibody against SARS-CoV-2 S protein and goat AF488-conjugated goat anti-rabbit antibody at a 1:200 dilution corresponding to an antibody against pMLKL were applied and incubated at 37°C for 1h.

**Determination of fatty acid oxidation (FAO) and oxygen consumption rate (OCR)**

To measure FAO activity in the untreated and the treated cells after infection with IAV or SARS-CoV-2, 1 x 10^4^ cells grown in a 96-well plate were used for a colorimetric FAO assay kit (AssayGenie) as previously reported.^17^ In brief, Vero E6 and A549 cells infected with SARS-CoV-2 KCDC03 strain at an MOI of 0.1 and IAV PR8 strain at an MOI of 1, respectively, were vehicle-treated or treated with 20 μM atglistatin or CAY10499 at 18 h for SARS-CoV-2 and 12 hpi for IAV, and incubated further for 18 h and 12 h, respectively (Table S3). Collected cells were lysed and the oxidation of octanoyl-CoA of the supernatant was measured by colorimetric reaction read at 492 nm. FAO activity was normalized to cell number as well as to protein content as determined by the bicinchoninic acid (BCA) assay (Thermo Scientific).

The OCR in live cells was measured using the Seahorse XFe96 analyzer from Agilent Technologies, as described previously.^18^ For quantifying endogenous FAO, A549 cells were plated at a density of 3 x 10^3^ cells in the XFe96 cell culture microplates overnight. Cells infected with IAV PR8 strain at an MOI of 1 were treated with 20 μM atglistatin or CAY10499 at 12 hpi and incubated further for 12 h. Cells were then starved in substrate-limited medium (DMEM containing 0.5 mM glucose, 1% FBS, 1 mM Glutamax, and 0.5 mM L-carnitine) for 12 h before it was replaced with FAO assay media (XF basal medium supplemented with 2 mM glucose, 0.5 mM L-carnitine). The assay was run with sequential injections of oligomycin, FCCP, and rotenone/antimycin A. Data were normalized to protein concentration.

**Measurement of proinflammatory cytokines in the cell culture supernatant and bronchoalveolar lavage fluid (BALF)**

The levels of proinflammatory cytokines (IFN-α, IFN-β, TNF-α, IL-6, and MCP-1) were detected from the supernatant of vehicle- or inhibitor-treated SARS-CoV-2-infected Vero E6 cells or IAV-infected A549 cells or BALF from vehicle- or inhibitor-treated SARS-CoV-2-challenged hamsters or IAV-challenged mice as described previously.^19^ In brief, A549 and Vero E6 cells infected with IAV PR8 strain and SARS-CoV-2 KCDC03 strain at an MOI of 0.1, respectively, were vehicle-treated or treated with 20 μM atglistatin or CAY10499 at 12 hpi for IAV and 18 h for SARS-CoV-2, and incubated further for 12 h and 18 h (Table S3), respectively. To detect proinflammatory cytokines in the BALF, the trachea of experimental hamsters and mice was cannulated using an 18-gauge and 20-gauge catheter. Bronchoalveolar lavage (BAL) was performed twice by instilling ice-cold sterile PBS (pH7.4) in an amount of 2 mL for hamsters and 0.4 mL for mice. Acquired BAL fluid was spun at 1,000 x g for 5 min at 4°C, and the supernatant was divided and stored at -70°C. Afterward, supernatant from each group was harvested for the detection of IFN-α, IFN-β, TNF-α, IL-6, and MCP-1 using a sandwich enzyme-linked immunosorbent assay (ELISA) kit from Thermo Scientific.

**Experimental animals**

To determine the survival rate or gross lung lesion, virus replication, changes of the host genes, and pathological changes in response to IAV or SARS-CoV-2 infections and chemical treatments, seven weeks old, female wild-type (WT) C57BL/6J mice and eleven weeks old, female WT Golden Syrian hamsters were purchased from Samtako (Osan, South Korea) and Janvier Labs (Saint-Berthevin, France), respectively. The mice were kept in standard cages and exposed to a 12:12 hour light/dark cycle at 25°C with food and water *ad libitum* in a specific-pathogen-free facility. The hamsters were kept in standard cages and exposed to a 12:12 hour light/dark cycle at 22-24°C and 40-55% humidity with food and water *ad libitum* in an animal biosafety level three facility. After 1 week of acclimatization, mice and hamsters were used for each experiment as described below.

**Lung and blood distribution of atglistatin and CAY10499**

The distribution of atglistatin and CAY10499 in the lung and blood samples from experimental animals was determined by LC/MS as described previously with slight modification.^20^ Two groups of 4 anesthetized mice were treated intraperitoneally with either atglistatin or CAY10499 in a 50 μL vehicle [10% PEG400 in water (vol/vol)] containing 10 mg kg^-1^ d^-1^ Bid with a 6 h interval for one day (Table S9). One group of 4 anesthetized hamsters was treated intraperitoneally with atglistatin in a 100 μL vehicle [50% PEG400 in water (vol/vol)] containing 80 mg kg^-1^ d^-1^ Bid with a 6 h interval for one day (Table S9). Blood samples from the abdominal vena cava and lung samples of the experimental animals were collected 6 h after the last chemical treatment. Aliquots of 200~500 μL plasma were mixed with 700 μL of cold acetonitrile and vortexed for 1 min to precipitate the proteins. The mixture was sonicated for 3 min and re-vortexed for 30 sec. The precipitated proteins were removed by centrifugation at 13,000 rpm for 10 min, and aliquots (350~400 μL) of the clear supernatants placed into a micro-tube. For lung tissues, 250 ~ 800 mg of tissues were placed at bead tube and mixed with 1 mL of cold acetonitrile. The mixture was homogenized at 4260 rpm for 30 sec, 3cycle in 4°C. The extracts were centrifuged at 10,000 x g for 10 min, and aliquots of the clear supernatants placed into a micro-tube. Using vacuum concentrator, the supernatants were dried. Finally, the dried extracts were re-dissolved by acetonitrile (atglistatin) and ethanol (CAY10499) and centrifuged at 13,000 rpm for 5 min. The supernatants were transferred into LC-MS vials. LC-MS was performed with an Acquity UPLC I-Class Plus system coupled to a Waters XEVO TQ-S (triple quodrupole) MS system (Waters, Milford, MA), equipped with an electrospray source operated in the positive-ion mode. An aliquot of the extracted sample (10 μL) was injected into an Acquity UPLC BEH-C18 reversed-phase column (2.1 X 50 mm column size, 1.7 μm particle size) with two solvents: (A) DW + 0.1% formic acid and (B) acetonitrile + 0.1% formic acid. A gradient programmer was used according the following profile: 0-4 min, 10-90% (B); 4-5 min hold on, 90% (B); 5-6 min, 90-30% (B). The flow rate during the experiment was 0.3 mL/min. The ESI-MS data were acquired in positive ion mode and conditions of MS analysis were as follows: capillary and sampling cone voltages were respectively set at 3.25 kV and 80 (atglistatin) or 60 V (CAY10499). The desolvation temperatures were 250 (atglistatin) and 200°C (CAY10499), and the cone and desolvation gas flow rates were set at 150 and 550 L/h. The standard compounds as well as extracted samples were analyzed in selective ion monitoring (SIM) mode using the ESI source by monitoring the H^+^ adducts ions. A series of standard solutions with seven (Atglistatin) and ten (CAY10499) different concentrations were analyzed by an established method in quintuple. Calibration curves were linear with correlation coefficients (R^2^) above 0.9699 (Atglistatin) and 0.9993 (CAY10499) for all analytes

***In vivo* organ-specific toxicity**

Six groups of 3 anesthetized mice were treated intraperitoneally with either atglistatin or CAY10499 in a 50 μL vehicle [10% PEG400 in water (vol/vol)] containing 0.1, 1, or 10 mg kg^-1^ d^-1^ Bid with a 12 h interval for four consecutive days, respectively (Table S10). One group of 3 control anesthetized mice was administered intraperitoneally with 100 μL vehicle Bid with a 12 h interval for four consecutive days (Table S10). Three groups of 3 hamsters were treated intraperitoneally with 20, 40, and 80 mg kg^-1^ d^-1^ of atglistatin prepared in a 100 μL vehicle [50% PEG400 in water (vol/vol)] Bid with a 12 h interval for four and a half consecutive days, respectively (Table S10). One group of 3 control anesthetized hamsters was administered intraperitoneally with 100 μL vehicle Bid with a 12 h interval for four and a half consecutive days (Table S10). Gross and histopathological changes in lungs, liver, kidney, heart, and spleen from the experimental mice or hamsters in each experimental group were determined as described below.

**Determination of median lethal dose (LD_50_) of mouse-adapted PR8 strain**

Adaptation of IAV PR8 (H1N1) strain in mice was performed as described elsewhere.^21,22^ In brief, female 6-week-old BALB/c mice were inoculated intranasally under appropriate anesthesia (Zoeltil/Xylazine) with 50 μl of cell-passaged supernatant of IAV PR8 strain. Lungs were harvested at four days post-infection (dpi) and homogenized, and 50 μl of the centrifuged homogenate followed by syringe filtration (0.2 μm) was used as the inoculum for the next passage. After a total of nine passages, IAV present in the lung homogenate was cloned once by plaque purification in MDCK cells. The cloned virus was passaged once in the allantoic cavities of 12-day-old chicken eggs for 48 h at 37^o^C to prepare the virus stock.

The median lethal dose (LD_50_) of the mouse-adapted PR8 strain was determined by infecting 8-week-old mice, as described previously.^23-25^ Briefly, C57BL/6J WT mice (n = 60) were randomly divided into five groups. Four groups of twelve mice were anesthetized by intraperitoneal injection with a combination of Zoletil and Xylazine (5 mg/kg and 10 mg/kg, respectively) and then infected once by intranasal inoculation of 10^1^ to 10^4^ PFU of PR8 strain in 50 μL PBS, respectively. Twelve control anesthetized mice were mock-infected by intranasal inoculation with 50 μL PBS. Animal survival and body weight on the basis of daily evaluation for 15 days were recorded, and the clinical illness of each mouse was evaluated using the following scoring system; 0 = no visible signs of disease; 1 = slight ruffling of fur; 2 = ruffled fur, reduced mobility; 3 = ruffled fur, reduced mobility, rapid breathing; 4 = ruffled fur, minimal mobility, huddled appearance, rapid and/or labored breathing indicative of pneumonia.^23-25^ The LD_50_ was calculated as described previously and expressed as the fold change in LD_50_.^23-25^

***In vivo* antiviral activity, lipid metabolism, and pathogenicity**

To examine the antiviral effects of atglistatin and CAY10499 against IAV infection in the mouse model, ten groups of 16 mice were mock-challenged or challenged once by intranasal inoculation of 10^3^ PFU of mouse-adapted PR8 strain in 50 μL PBS, respectively and administered intraperitoneally with either atglistatin or CAY10499 in 50 μL vehicle [10% PEG400 in water (vol/vol)] containing 0.01, 0.1, 1, 5, or 10 mg kg^-1^ d^-1^ twice daily (Bid) with a 12 h interval for four consecutive days, respectively (Tables S11 and S12). Four groups of 16 control anesthetized mice were mock-challenged or challenged by intranasal inoculation with 50 μL PBS and then treated intraperitoneally with 50 μL vehicle Bid with a 12 h interval for four consecutive days (Tables S11 and S12). The animals in each group were monitored daily by checking body weight, clinical signs, and survival for 15 days. As shown in Table S13, antiviral effects of atglistatin against SARS-CoV-2 infection in the Syrian hamster model were investigated. Three groups of 5 anesthetized hamsters were challenged once by intratracheal inoculation of 10^5^ TCID_50_ of SARS-CoV-2 KCDC03 strain in 100 μL PBS, and administered intraperitoneally with 20, 40, and 80 mg kg^-1^ d^-1^ of atglistatin prepared in 100 μL vehicle [50% PEG400 in water (vol/vol)] Bid with a 12 h interval for four and a half consecutive days, respectively. Two groups of 5 control anesthetized hamsters were mock-challenged or challenged by intratracheal inoculation with 100 μL PBS and then administered intraperitoneally with 100 μL vehicle Bid with a 12 h interval for four and a half consecutive days (Table S13). The animals in each group were monitored daily by checking body weight and survival for five days. Whole lung tissues were sampled from surviving and dead hamsters in each group at five dpi and the gross lung lesion surface was measured using NIH ImageJ software.

To examine the synergistic anti-IAV effects of either atglistatin or CAY10499 with oseltamivir, two groups of 16 anesthetized mice were challenged once by intranasal inoculation of 10^3^ PFU of mouse-adapted PR8 strain in 50 μL PBS and administered with either 5 mg kg^-1^ d^-1^ atglistatin or 5 mg kg^-1^ d^-1^ CAY10499 in 50 μL vehicle by intraperitoneal injection and subsequently administered oral gavage (19 gage) with 2 mg kg^-1^ d^-1^ of oseltamivir Bid with a 12 h interval for four consecutive days, respectively (Tables S14 and S15). Four groups of 16 anesthetized mice were challenged once by intranasal inoculation of 10^3^ PFU of PR8 strain in 50 μL PBS and treated with either 5 mg kg^-1^ d^-1^ atglistatin or 5 mg kg^-1^ d^-1^ CAY10499 in 50 μL vehicle by intraperitoneal injection or administered by oral gavage (19 gage) with 2 mg kg^-1^ d^-1^ of oseltamivir Bid with a 12 h interval for four consecutive days, respectively (Tables S14 and S15). Four groups of 16 control anesthetized mice were mock-challenged or challenged by intranasal inoculation with 50 μL PBS and then administered intraperitoneally with 50 μL vehicle Bid with a 12 h interval for four consecutive days. The animals were monitored daily to check body weight, clinical signs, and survival for 15 days post-inoculation.

We examined the effects of combination therapy of atglistatin and remdesivir on infection of three SARS-CoV-2 strains, KCDC03, KDCA51463, and KDCA55905, in the hamster model (Tables S16-S18). In each trial, one group of 5 anesthetized hamsters was challenged once by intratracheal inoculation of 10^5^ TCID_50_ with one of the SARS-CoV-2 strains, either KCDC03, KDCA51463, or KDCA55905 in 100 μL PBS, respectively and administered intraperitoneally with atglistatin (40 mg kg^-1^ d^-1^) and remdesivir (2.5 mg kg^-1^ d^-1^) each in 100 μL vehicle Bid with a 12 h interval for four and a half consecutive days, respectively (Tables S16-S18). Two groups of 5 challenged hamsters in each trial were administered intraperitoneally with atglistatin (40 mg kg^-1^ d^-1^) in 100 μL vehicle [50% PEG400 in water (vol/vol)] or remdesivir (2 mg kg^-1^ d^-1^) in 100 μL vehicle [10% DMSO in corn oil (vol/vol)] Bid with a 12 h interval for four and a half consecutive days, respectively. Another two groups in each trial were mock-challenged or challenged by intratracheal inoculation of 10^5^ TCID_50_ of each SARS-CoV-2 strain in 100 μL PBS and then administered intraperitoneally with 100 μL vehicle Bid with a 12 h interval for four and a half consecutive days (Tables S16-S18). The animals were monitored daily by checking body weight and survival for five days. Whole lung tissues were sampled from surviving and dead hamsters in each group at five dpi and the gross lung lesion surface was measured as described above.

To determine virus replication levels, changes of host gene mRNA levels, lipid metabolism, and histopathological changes in lung tissues from mock- or IAV-infected mice with or without therapeutic treatments, 3 groups of four 8-week-old female WT C57BL/6J anesthetized mice were infected with intranasal inoculation of 10^3^ PFU of mouse-adapted IAV PR8 strain in 50 μL PBS, and one group of four 8-week-old anesthetized mice were inoculated with 50 μL PBS as a negative control (Table S19). Among the virus-challenged groups, two groups were administered with 100 μL of PBS containing 5 mg kg^-1^ d^-1^ of atglistatin or CAY10499 Bid for four consecutive days via intraperitoneal injection (Table S19). Mice in either mock-challenged, mock-administered or IAV-challenged, mock-administered groups were treated with 100 μL of vehicle Bid for four consecutive days via intraperitoneal injection (Table S19).

To determine the inhibitory effect of atglistatin and CAY10499 on the spread of IAV through viremia, 3 groups of three 8-week-old female WT C57BL/6J anesthetized mice were infected with intranasal inoculation of 10^3^ PFU of mouse-adapted IAV PR8 strain in 50 μL PBS, and one group of three 8-week-old anesthetized mice were inoculated with 50 μL PBS as a negative control (Table S20). Among the virus-challenged groups, two groups were administered with 100 μL of PBS containing 5 mg kg^-1^ d^-1^ of atglistatin or CAY10499 Bid for four consecutive days via intraperitoneal injection (Table S20). Mice in either mock-challenged, mock-administered or IAV-challenged, mock-administered groups were treated with 100 μL of vehicle Bid for four consecutive days via intraperitoneal injection (Table S20). Blood samples were collected from the abdominal vena cava of three experimental mice from each group at 4 dpi as described above method. For SARS-CoV-2 experiments, 4 groups of five 12-week-old female WT anesthetized hamsters were challenged with intratracheal inoculation of 10^5^ TCID_50_ of SARS-CoV-2 KCDC03 strain in 100 μL PBS, and one group of five 12-week-old anesthetized hamsters were inoculated with 100 μL vehicle as a negative control (Table S21). Among the virus-inoculated groups, three groups were administered intraperitoneally with 100 μL of vehicle containing 20, 40, or 80 mg kg^-1^ d^-1^ of atglistatin Bid for four and a half consecutive days via intraperitoneal injection, respectively (Table S21). Hamsters in either mock-challenged, mock-administered or SARS-CoV-2-challenged, mock-administered groups were treated with 100 μL of vehicle Bid for four and a half consecutive days via intraperitoneal injection (Table S21). After sacrificing the mice and hamsters, entire lungs were excised from the thorax and subjected to further analysis under aseptic conditions. The left lobe (largest) was cut in half and fixed for histopathological evaluation and cryosection in 10% neutral formalin and 4% PFA, respectively, and the remaining right cranial, middle, caudal, and accessory lobes were combined and thoroughly minced with scissors. The finely cut lung homogenates were divided into several tubes of the same weight for application in the following experiments; 1) analysis of lipid metabolites through quantification of triacylglyceride (TAG), cholesterol, free fatty acid, and free glycerol, 2) determination of infectious virus titer, 3) viral and cellular target protein expression levels, 4) viral genome and cellular mRNA expression levels.

**Plaque assay**

To evaluate the infectivity titer of IAV, a plaque assay was performed as described elsewhere.^13,23^ Briefly, confluent MDCK cells grown in 24-well plates were mock-infected or infected with IAV at an MOI of 0.01 or 1, treated with or without different concentrations of inhibitors at 12 hpi, or transfected with siRNAs against target genes or scrambled siRNA as described above. After three cycles of freezing and thawing cycle, serial 10-fold dilutions of each cell culture supernatant were prepared in DMEM supplemented with 100 U/mL penicillin and 100 μg/mL streptomycin. To determine the viral infectivity titer in lung tissues from the mice challenged with or without IAV, or administered with or without inhibitors as described above, aseptically sampled lungs from four mice per group at day six after virus infection were harvested, and the weights of tissues recorded. Lung tissue samples were mechanically homogenized in virus growth medium (10% wt/vol) by using a Precellys 23 tissue homogenizer (Bertin Technologies, France), and the homogenates were then centrifuged at 1,200 × g for 10 min at 4°C; the cleared supernatants were serially tenfold diluted with virus growth medium. Two hundred microliters of each tenfold serial dilutions of cell culture supernatants and lung samples were transferred in quadruplicate to MDCK cells grown in 6-well plates. After 1 h adsorption at 37°C with horizontal rotation at 15 min intervals, each well was washed three times with DMEM and then replaced with 4 mL of agar overlay medium containing 1 x DMEM, 1 μg/mL of TPCK-treated trypsin and 0.5% ultrapure agarose (Thermo Scientific). After incubation for three days at 37°C in a CO_2_ incubator, the agar overlay medium was removed, and the cells were fixed with 4% buffered PFA for 10 min, rinsed with PBS and stained with 0.1 mg/mL crystal violet for 30 min at 20°C. The number of CPE plaques at the endpoint dilution were counted. The 50% inhibitory concentration (IC_50_) was calculated using GraphPad Prism software version 8.4.2. The selectivity index (SI) of each chemical was determined by calculating following ratio; 50% cell cytotoxicity concentration (CC_50_) / 50% inhibitory concentration (IC_50_). CC_50_ data from the MTT assay and IC_50_ from the plaque assay were both calculated using GraphPad Prism software version 8.4.2.

**Median Tissue culture infectious dose (TCID_50_) assay**

To determine the titer of SARS-CoV-2 strains, a TCID_50_ assay was performed with the Vero E6 cells as described elsewhere.^9^ Ten-fold serial dilutions of stock viruses were prepared in DMEM, from which 100 µl each was transferred onto monolayers of Vero E6 cells grown on 96-well plates in DMEM supplemented with 1 μg/ml TPCK-treated trypsin and incubated at 37°C in a 5% CO2 incubator. Virus titers were calculated at four dpi and expressed as TCID_50_/mL values by the method of Reed and Muench.^26^

**Immunofluorescence assay (IFA)**

To characterize the dynamics of LD formation, apoptotic and necroptotic cells, and virus infectivity, an IFA was performed with the A594, MDCK, Caco-2, Vero E6, MA104, MARC-145, LLC-PK, and HRT-18G cells, infected with or without each virus, and treated with vehicles or chemicals at the different time points (Table S3), or transfected with siRNA or scrambled siRNA as described above. ^16,27,28^ Briefly, cells were prepared in 8-well chamber slides infected with IAV, SARS-CoV-2, BCoV, PEDV, RVA, PRRSV, or PSaV at an MOI of 1 (IAV), or MOI of 0.1 (SARS-CoV-2, BCoV, PEDV, RVA, PRRSV, or PSaV) and treated with or without chemicals. The cells were fixed with 4% buffered PFA for 10 min at 20°C, washed twice with DPBS, and permeabilized with 0.2% Triton X-100 for permeabilization for 10 min at 20°C. Antibodies against the target cellular or viral proteins were applied overnight at 4°C, and washed thrice with PBS (pH 7.4) containing 0.1% new born calf serum (PBS-NCS). AF488 or AF647 conjugated secondary antibodies were incubated for 1 h at 20°C. For determining LD formation, selected slides were further incubated with 10 μM Bodipy 493/503 for 10 min at 20°C. Subsequently, cells were washed thrice with PBS (pH 7.4) and mounted with SlowFade Gold antifade reagent containing 1 × DAPI solution (Molecular Probes) for nucleus staining, observed with a LSM 800 confocal microscope, and analysed using Zen Blue Software 2.6 (Carl Zeiss; Jena, Germany).

To determine the antiviral effects of chemicals, serial 10-fold dilutions of each cell culture supernatant after three cycles of freezing and thawing cycles were prepared in appropriate medium supplemented with 100 U/mL penicillin and 100 μg/mL streptomycin as described above. To determine the viral infectivity titer in lung tissues from the hamsters challenged with or without SARS-CoV-2, or administered with or without inhibitors as described above, aseptically sampled lungs from five hamsters per group at day five post-virus infection were harvested. Cleared supernatants from lung tissue as produced from IAV-infected mice above were serially tenfold-diluted with virus growth medium. One hundred microliters of the tenfold serially diluted cell culture supernatants and lung samples were transferred in quadruplicate to cells grown in 96-well plates and incubated for the indicated time points at 37°C in a CO_2_ incubator; 12 h incubation for RVA, 18 h incubation for SARS-CoV-2 and the other viruses. The cells were washed twice with PBS (pH 7.4) and primary antibodies against IAV M2, SARS-CoV-2 N, BCoV S, PEDV N, RVA VP6, PRRSV M, and PSaV VPg proteins were applied to each of the corresponding wells. After washing twice with PBS (pH 7.4), FITC-conjugated secondary antibodies specific for each primary antibody were incubated for 1 h at 20°C. The cells were washed twice with PBS (pH 8.0), mounted with 60% glycerol, and observed byr inverted fluorescence microscopy. The number of cells showing a viral antigen-positive signal at the endpoint dilution were counted. IC_50_ was calculated using GraphPad Prism software version 8.4.2. SI of the chemicals was determined by calculating the following ratio; 50% cell cytotoxicity concentration (CC_50_) / 50% inhibitory concentration (IC_50_). CC_50_ data from the MTT assay and IC_50_ from the IF assay were both calculated using GraphPad Prism software version 8.4.2.

An IFA was also carried out with the lung sections from mice and hamsters as described elsewhere with slight modification.^16,27,28^ Briefly, 3 μM cryosections of 4% buffered PFA-fixed lungs from experimental mice or hamsters were allowed to completely air-dry and were then washed twice with PBS-NCS. Each section was incubated with primary antibodies against the target cellular or viral proteins overnight at 4°C, and washed thrice with PBS-NCS. AF647 conjugated secondary antibodies were incubated for 1 h at 20°C. For determining LD formation, selected slides were further incubated with 10 μM Bodipy 493/503 for 10 min at 20°C. Subsequently, lung tissues washed thrice with PBS (pH 8.0) were mounted with SlowFade Gold antifade reagent containing 1 × DAPI solution (Molecular Probes) for nucleus staining, observed with an LSM 800 confocal microscope, and analyzed using LSM software (Carl Zeiss; Jena, Germany).

The number and size of intracellular LDs were measured as described previously.^28^ Briefly, four images were taken of each sample and converted into a pixel unit, and the data were processed and quantified using Adobe Photoshop CS6. Calculations were based on the size and number of pixels with positive colorization that was present in approximately 40 cells. The results were expressed as relative values, where the average number and size of pixels of positive color in virus-infected groups were normalized to those of the mock-treated, mock-infected control.

**Western blot analysis**

To evaluate the expression levels of target cellular and viral proteins in the culture cells or lung tissues, western blot analysis was performed as described elsewhere.^9,16^ Briefly, cells or homogenized lung tissues were lysed with RIPA buffer containing 10 mM Tris/HCl (pH 7.4), 100 mM NaCl, 1 mM ethylenediaminetetraacetic acid (EDTA), 1 mM ethylene glycol-bis(2-aminoethylether)-N,N,N’,N’-tetraacetic acid (EGTA), 1 mM NaF, 20 mM Na_2_P_2_O_7_, 2 mM Na_3_VO_4_, 1% Triton X-100, 10% glycerol, 0.1% sodium dodecyl sulfate (SDS), and 0.5% deoxycholate (Invitrogen) for 10 min on ice. The supernatant from the resulting lysate was acquired after centrifugation at 12,000 × g for 10 min at 4°C, and total protein content was determined by a BCA protein assay kit (Thermo Scientific). Normalized protein samples were electrophoresed via sodium dodecyl sulphate-polyacrylamide gel electrophoresis (SDS-PAGE) and transferred onto nitrocellulose membranes (GE Healthcare Life Sciences). The membranes were blocked for 1 h at 20°C with Tris-buffered saline containing 5% skimmed milk and 0.1% Tween-20, followed by incubation overnight at 4°C with each primary antibody on a shaker. After incubation with a horseradish peroxidase (HRP)-conjugated secondary antibody for 1 h at 20°C, bound antibodies to the target proteins were developed by enhanced chemiluminescence (ECL) (Dogen, Seoul, South Korea). Detected bands were photographed with a Davinch-K Imaging System (Youngwha Scientific Co., Ltd, Seoul, South Korea). The intensity of each target protein band was normalized to the β-actin as an internal control, and the relative values were presented as a mean-fold changes.

**Triglyceride colorimetric assay**

The quantity of intracellular TAGs was measured using a triglyceride colorimetric kit (Cayman Chemicals) according to the manufacturer’s recommendations.^29^ Briefly, A549, MDCK, Vero E6, and Caco-2 cells infected with or without IAV, Vero E6 cells infected with or without SARS-CoV-2, or treated with or without different concentrations of chemicals, and lung samples from experimental mice or hamsters were prepared as described above. The number of cells for the assay were counted by hemocytometer after trypsinization and staining with methylene blue. Afterward, 5x10^6^ cells were mixed with 500 μL of 1 × Standard Diluent and subjected to sonication (VCX130, SONICS, Newtown, CT, USA) under optimally timed operational conditions on the ice; that is, one second burst and five seconds of break for 20 cycles. After centrifugation, the acquired supernatant was diluted 1:2 with 1 × Standard Diluent. For determining TAG levels in the lung tissues from each *in vivo* experiment, 500 μL 1 × NP40 Substitute Assay Reagent was applied to 100 mg of lung homogenates, and sonication was carried out with following interval; 2/10 seconds (burst/break) for 20 cycles. After centrifugation, the supernatant was diluted 1:5 with 1x NP40 Substitute Assay Reagent. Each supernatant from cultured cells or lung tissue was transferred into 96-well plate in a volume of 10 μL and analyzed in conjunction with the supplied TG standard solution prepared by serial dilution method. After incubation with 150 μL 1x Triglyceride Enzyme Mixture for 15 min at 20°C, the colorimetric changes were determined by measuring the optical densities (OD) at 570 nm using a Multiskan Sky 1530 ELISA reader (Thermo Scientific) and calculations were performed, and a standard curve generated according to the manufacturer's instructions. The results were expressed as relative values, where the quantity of TAGs in the virus-infected groups were normalized against the one in mock-treated, mock-infected control.

**Cholesterol colorimetric assay**

To determine intracellular cholesterol levels, A549, MDCK, Vero E6, and Caco-2 cells infected with or without IAV, Vero E6 cells infected with or without SARS-CoV-2, or treated with or without different concentrations of chemicals, and lung samples from experimental mice or hamsters were prepared as described above. The quantity of intracellular cholesterols was measured using a cholesterol colorimetric assay kit (Abcam) according to the manufacturer’s recommendations.^30^ The number of cells applied to this assay was counted as described for the TAG colorimetric assay. A pellet in the number of 5x10^6^ cells was incubated with 500 μL 1x Cholesterol Assay Buffer with vortexing every minute for 20 min on ice. After centrifugation, the supernatant was collected. For determining cholesterol levels in the lung tissues from each *in vivo* experiment, 500 μL 1x Cholesterol Assay Buffer was applied to 100 mg of lung homogenates and incubated for 20 min on ice with vortexing every minute. After centrifugation, 50 μL of supernatant from cultured cells or lung tissue was transferred into a 96-well plate in parallel with the supplied cholesterol standard solution prepared by seral dilution. After incubation with 50 μL 1x Cholesterol Reaction Mix containing Enzyme Mix and Cholesterol Esterase at 37°C for 60 min, the colorimetric change was determined by measuring the optical density (OD) at 570 nm using an ELISA reader, and calculation was performed, followed by generation of a standard curve according to the manufacturer's instructions. The results were expressed as relative values, where the quantities of cholesterol in virus-infected groups were normalized against the mock-treated, mock-infected control.

**Free fatty acid quantification**

To determine intracellular levels of free fatty acids, the different cultured cells infected with or without the seven target RNA viruses, or treated with or without different concentrations of chemicals, and the lung samples from experimental mice and hamsters were prepared as described above. The amount of intracellular free fatty acid was determined using a quantification kit from BioVision according to the manufacturer’s recommendations.^31^ Briefly, 5x10^6^ cells or 100 mg of lung tissue were mixed with 500 μL of chloroform-Triton X-100 (1% Triton X-100 in pure chloroform) with vortexing at five-minute intervals for 20 min at 20°C. After collecting organic phase (lower phase), chloroform was thoroughly air dried for 20 min at 60°C. The dried lipids were then dissolved in 400 μL of Fatty Acid Assay Buffer by vortexing extensively for 5 min at 20°C, and 50 μL of supernatant was transferred on to a 96-well plate in conjunction with the serially diluted standard samples. Two microliters of ACS Reagent were added to each well and incubated at 37°C for 30 min. Afterward, all samples and standards received 50 μL of 1x Reaction Mixture, which comprised of Enzyme Mix and Enhancer, and was incubated at 37°C for 30 min. Measurement was performed by determining the OD at 570 nm using an ELISA reader and calculations were made, and followed by the generation of a standard curve, according to the manufacturer's instructions.

**Free glycerol quantification**

To determine the intracellular glycerol levels, the different cell cultures infected with or without the seven target RNA viruses, or treated with or without different concentrations of chemicals, and the lung samples from experimental mice or hamsters were prepared as described above. The amount of intracellular free glycerol was determined using a quantification kit from BioVision according to the manufacturer’s recommendations.^32^ Briefly, 5x10^6^ cells or 100 mg of lung tissue were mixed with 1 mL of 1x Glycerol Assay Buffer with vortexing at five-minute intervals for 20 min at 20°C. After centrifugation, 50 μL of supernatant was transferred to a 96-well plate in parallel with the serially diluted glycerol standard samples. Subsequently, 50 μL of 1x Reaction Mixture, including the Enzyme Mix and Glycerol Probe, was added and incubated at 20°C for 30 min. Measurement was performed by determining the OD at 570 nm using an ELISA reader, calculations were made and a standard curve generated according to the manufacturer's instructions.

**Quantitative real-time PCR**

The target viral RNAs and host mRNAs in both cultured cells and lungs and sera from experimental mice or hamsters were quantified using real-time PCR as described previously with slight modifications.^13,33^ Briefly, total RNA from the cultured cells and homogenized lung tissues was extracted using an RNeasy kit (Qiagen) according to the manufacturer’s protocol. The corresponding cDNA was synthesized using the TOPscript™ cDNA Synthesis kit (Enzynomics). Each reaction included a total volume mixture of 20 μL, which contained 5 μL of RNA template (1 μg), 2 μL TOPscript™ RT buffer, 1 μL of TOPscript™ reverse transcriptase, 2 μL dNTP mixture (2 mM), 0.5 μL of Oligo (dT), 0.5 μL of the random hexamer, 0.5 μL of RNase inhibitor, and 8.5 μL of RNase free water according to manufacturer’s instructions.

First-strand cDNA samples were subjected to quantitative real-time PCR using a TOPreal™ qPCR 2X PreMix (Enzynomics), and the LineGene 9600 Plus Real-time PCR detection system (Bioer, Hangzhou, China) was applied with the following conditions using specific primer pairs for viral and cellular target genes (Table S22): for viral genome copy numbers, activation of the DNA polymerase at 95°C for 10 min and 40 cycles of three steps of 95°C for 15 s, 60°C for 20 s, and 72°C for 20 s, and for cellular target gene mRNA, activation of the DNA polymerase at 95°C for 10 min and 40 cycles of three steps of 95°C for 15 s, 55°C for 20 s, and 72°C for 20 s. The threshold was automatically defined in the initial exponential phase, reflecting cycle threshold (CT) values at the highest amplification rate. Relative amounts of RNAs were calculated by using the comparative CT method.^34^ The mRNA levels of genes involved in fatty acid and cholesterol biosynthesis of virus-inoculated cells or tissues were expressed as a fold change of mRNA levels compared to the mock-inoculated, mock-treated control. The reduction in viral load was expressed as relative values, where genome copy numbers in virus-infected, chemical-treated groups were normalized against the virus-infected, mock-treated control.

**Palmitoylation assay**

To check the influence of the lipase inhibitor CAY10499 on palmitoylation of SARS-CoV-2 S protein or IAV HA and M2 proteins in virus-infected cells, post-translationally modified proteins in the cell lysates at cysteine residue with a palmitic acid through thioester formation (S-palmitoylation) were analyzed by the CAPTUREomeTM S-palmitoylated protein kit (Badrilla) according to manufacturer’s instructions.^13^ Briefly, Vero E6 cells grown in 6-well plates were infected with or without SARS-CoV-2 at an MOI of 0.1 for 18 h, treated with or without 20 μM of CAY10499, and incubated for a further 18 h, while A549 cells infected with or without IAV at an MOI of 1 for 12 h were treated with or without 20 μM of CAY10499 and incubated for a further 12 h at 37°C in the CO_2_ incubator. A total of 2x10^6^ cells were washed with PBS (pH 7.4) and collected at 36 h after SARS-CoV-2 infection and 24 h after IAV infection. The cells were incubated in 400 μL of Buffer A including Thiol Blocking Reagent at 40°C for 4 h in a shaking heat block. Dissolved proteins were precipitated by the addition of 1.2 mL of 70% acetone. After centrifugation, the supernatant was removed. These steps were repeated five times to ensure thorough washing of the sample. The pellet was dissolved with 300 μL 1x Binding Buffer and incubated at 40°C for 30 min in a shaking heat block. After centrifugation, the acquired supernatant was divided into 2 parts, 50 μL for input control as a total protein lysate and 250 μL for further treatment for the capture of palmitoylated proteins. The input control was mixed with 50 μL of 2x Laemmli Sample Buffer at 60°C for 10 min and stored at -20°C. For purification of S-palmitoylated protein, 50 μL CAPTUREome^TM^ Capture Resin and 19 μL Thio Cleavage Reagent were add to the supernatant, leading to thioester bonds cleaved from palmitic acid and liberated thiols bound to resin–assisted acyl-Rac. This reaction was performed on a rotary wheel at 20°C for 3 h. After centrifugation, the supernatant was removed, and resin was washed with 1 mL of 1x Binding Buffer a total of five times. After the final wash, captured proteins were eluted with 100 μL of 2x Laemmli Sample Buffer at 60°C for 10 min, and the supernatant was collected. To measure the palmitoylation level in proportion to the total amount of IAV HA and M2 proteins, and SARS-CoV-2 S protein, the purified S-palmitoylated protein samples, and the input controls were loaded in parallel on an SDS-PAGE gel. Immunoblotting with antibodies targeting IAV HA and M2 proteins and SARS-CoV-2 S protein was carried out as described in the western blot analysis section. The percent reduction rate of protein palmitoylation in the virus-inoculated, chemical-treated group was calculated using the following formula, [(palmitoylated protein/input palmitoylated protein) in the virus-infected, chemical-treated group/(palmitoylated protein/input palmitoylated protein) in the virus-infected, mock-treated group)] × 100%.

**Histopathology**

Histopathological changes in lungs, liver, kidney, heart, and spleen from the experimental mice or hamsters in each experimental group were determined as described elsewhere.^35^ Briefly, each sample from the mice or hamsters in each experimental group was fixed in sufficient 10% neutral formalin at 20°C for 3-4 days, washed in tap water, then trimmed, and thereafter processed in the automatic embedding machine for the dehydration and paraffin embedment. Tissue sections, prepared on slides at a thickness of 3 μm by a microtome, were used for hematoxylin and eosin (H&E) staining or IHC as described below. H&E stained sections were observed under light microscopy. The morphological changes to lung, liver, kidney, heart, and spleen tissues were reviewed in a blinded manner as previous described.^35^

**Immunohistochemistry (IHC)**

To determine the expression levels of IAV or SARS-CoV-2 proteins in the lung tissue, IHC was performed with 3-μm-thick selected sections as described elsewhere with slight modification.^33^ In brief, paraffin-embedded tissue sections were deparaffinized, hydrated, and autoclaved in 10 mM citrate solution (pH 8.8) for antigen retrieval. Afterwards, slides were rinsed with PBS (pH 7.4) and treated with 3% H_2_O_2_ in double distilled water for 30 min to quench endogenous peroxidase activity, and then followed by blocking with 5% bovine serum albumin (BSA) in PBS (pH 7.4) for 1 h at 20°C. Each section was incubated overnight in a humid chamber at 4°C with an antibody against IAV virion or SARS-CoV-2 S protein, or PBS as a negative control. Incubation with HRP-conjugated secondary antibody and development by 3, 3'-diaminobenzidine (DAB) were carried out by using the Dako REAL™ EnVision™ Detection System (DakoCytomation, Denmark), according to the manufacturer’s instructions. After slight counterstaining with hematoxylin, the slides were dehydrated, cleared in xylene, and mounted.

**Illustrations**

Illustrations of animals in Supplementary Fig. S18 and S19 were created with BioRender software (https://biorender.com/).

**Supplementary Tables**

**Table S1. Comparison of fatty acid compositions between mock- and IAV-infected A549 cells**

| **FAME name** | **R.T. (min)** | **Height (intensity, µV)** | | **IAV / Mock**  **(F.C.)** |
| --- | --- | --- | --- | --- |
|  |  | **MOCK** | **IAV** |  |
| Myristic acid (C14:0) | 8.961 | 2357 | 9148 | 3.88 |
| Pentadecylic acid (C15:0) | 9.753 | N.D. | 1111 | - |
| Cis-10-Pentadecenoic Acid (C15:1) | 10.175 | 4012 | 9847 | 2.45 |
| Palmitic acid (C16:0) | 10.663 | 31078 | 77452 | 2.49 |
| Palmitoleic acid (C16:1) | 11.005 | 4197 | 14376 | 3.43 |
| Margaric acid (C17:0) | 11.677 | 610 | 852 | 1.40 |
| Stearic acid (C18:0) | 12.818 | 15404 | 21073 | 1.37 |
| Oleic acid (C18:1 cis n9) | 13.171 | 47544 | 72640 | 1.53 |
| Linolelaidic acid (C18:2 trans n6) | 13.812 | 786 | 3380 | 4.30 |
| cis-gondoic acid (C20:1n9) | 15.743 | N.D. | 2342 | - |
| Dihomolinoleic acid (C20:2) | 16.527 | 9650 | 7032 | 0.73 |
| Heneicosylic acid (C21:0) | 16.797 | 1173 | 877 | 0.75 |
| Dihomo-γ-linolenic acid (C20:3n6) | 16.935 | N.D. | 1336 | - |
| Arachidonic acid (C20:4n6) | 17.220 | 5261 | 13253 | 2.52 |
| Tricosylic acid (C23:0) | 19.451 | 1092 | 1483 | 1.36 |
| **Subtotal of saturated fatty acids (SFAs) and unsaturated fatty acids (UFAs)** | | | | |
| SFAs |  | 51714 | 111996 | 2.17 |
| UFAs |  | 71450 | 124206 | 1.74 |
| UFAs/SFAs |  | 1.38 | 1.11 | 0.80 |

FAME, fatty acid methyl ester; R.T., retention time; F.C., fold change

**Table S2. The half maximal (50%) inhibitory concentration (IC_50_) and selectivity index (SI) of atglistatin and CAY10499 for seven RNA viruses**

| **Virus family** | **Virus (strain)** | **IC_50_ (μM)** | |  | **SI (CC_50_/IC_50_)^a^** | |
| --- | --- | --- | --- | --- | --- | --- |
|  |  | **Atglistatin** | **CAY10499** |  | **Atglistatin** | **CAY10499** |
| *Orthomyxoviridae* | Influenza A virus (H1N1) (PR8) | 0.82 ± 0.08 | 4.13 ± 0.46 |  | 251 | 27 |
| *Coronaviridae* | SARS-CoV-2 (KCDC03) | 1.47 ± 0.12 | 1.54 ± 0.18 |  | 154 | 62 |
| *Coronaviridae* | Bovine coronavirus (KWD20) | 1.20 ± 0.04 | 7.30 ± 0.52 |  | 152 | 14 |
| *Coronaviridae* | Porcine epidemic diarrhea  coronavirus (QIAP1401) | 1.96 ± 0.14 | 3.19 ± 0.42 |  | 116 | 30 |
| *Reoviridae* | Species A Rotavirus (NCDV) | 6.70 ± 0.49 | 20.85 ± 5.06 |  | 32 | 5 |
| *Arteriviridae* | Porcine respiratory reproductive syndrome virus (LMY) | 2.53 ± 0.18 | 4.21 ± 0.55 |  | 84 | 24 |
| *Caliciviridae* | Porcine sapovirus (Cowden) | 3.11 ± 0.39 | 11.76 ± 2.6 |  | 72 | 9 |

^a^CC_50_, median cytotoxic concentration (See Table S2).

**Table S3. The median cytotoxic concentration (CC_50_) of atglistatin and CAY10499 in different cell lines determined by measuring the cellular NAD(P)H-dependent cellular oxidoreductase enzymes (MTT assay)**

| **Cell line** | **CC_50_** | |
| --- | --- | --- |
|  | **Atglistatin** | **CAY10499** |
| A549 | 205.9 ± 3.0 | 105.7 ± 8.9 |
| Vero E6 | 226.4 ± 2.4 | 101.8 ± 8.3 |
| MDCK | 268.6 ± 3.2 | 110.0 ± 4.1 |
| HRT18G | 182.5 ± 2.6 | 103.5 ± 9.1 |
| MA104 | 214.5 ± 2.0 | 107.1 ± 5.6 |
| MARC | 211.6 ± 3.5 | 101.4 ± 4.0 |
| LLC-PK | 224.2 ± 2.3 | 108.9 ± 8.1 |

**Table S4. Experimental design for determining *in vitro* broad-spectrum antiviral effect of atglistatin and CAY10499 at different treatment times**

| **Viruses^a^** | **MOI** | **Drug concentration** | **Treatment at^b^** | **Incubation time** | **Treatment at^c^** | **Further incubation time^d^** | **Determination of virus replication** |
| --- | --- | --- | --- | --- | --- | --- | --- |
| IAV | 0.01 FFU | - | - | 24 hpi | - | 24 hpi | RT-qPCR, CCIF |
|  | 0.01 FFU | 1 μM | AVA | 24 hpi | 12 hpi | 12 hpi | RT-qPCR, CCIF |
|  | 0.01 FFU | 10 μM | AVA | 24 hpi | 12 hpi | 12 hpi | RT-qPCR, CCIF |
|  | 0.01 FFU | 20 μM | AVA | 24 hpi | 12 hpi | 12 hpi | RT-qPCR, CCIF |
| SARS-CoV-2 | 0.01 FFU | - | - | 36 hpi | - | 36 hpi | RT-qPCR, CCIF |
|  | 0.01 FFU | 1 μM | AVA | 36 hpi | 18 hpi | 18 hpi | RT-qPCR, CCIF |
|  | 0.01 FFU | 10 μM | AVA | 36 hpi | 18 hpi | 18 hpi | RT-qPCR, CCIF |
|  | 0.01 FFU | 20 μM | AVA | 36 hpi | 18 hpi | 18 hpi | RT-qPCR, CCIF |
| BCoV | 0.01 FFU | - | - | 36 hpi | - | 36 hpi | RT-qPCR, CCIF |
|  | 0.01 FFU | 1 μM | AVA | 36 hpi | 18 hpi | 36 hpi | RT-qPCR, CCIF |
|  | 0.01 FFU | 10 μM | AVA | 36 hpi | 18 hpi | 18 hpi | RT-qPCR, CCIF |
|  | 0.01 FFU | 20 μM | AVA | 36 hpi | 18 hpi | 18 hpi | RT-qPCR, CCIF |
| PEDV | 0.01 FFU | - | - | 36 hpi | - | 36 hpi | RT-qPCR, CCIF |
|  | 0.01 FFU | 1 μM | AVA | 36 hpi | 18 hpi | 36 hpi | RT-qPCR, CCIF |
|  | 0.01 FFU | 10 μM | AVA | 36 hpi | 18 hpi | 18 hpi | RT-qPCR, CCIF |
|  | 0.01 FFU | 20 μM | AVA | 36 hpi | 18 hpi | 18 hpi | RT-qPCR, CCIF |
| RVA | 0.01 FFU | - | - | 24 hpi | - | 24 hpi | RT-qPCR, CCIF |
|  | 0.01 FFU | 1 μM | AVA | 24 hpi | 12 hpi | 12 hpi | RT-qPCR, CCIF |
|  | 0.01 FFU | 10 μM | AVA | 24 hpi | 12 hpi | 12 hpi | RT-qPCR, CCIF |
|  | 0.01 FFU | 20 μM | AVA | 24 hpi | 12 hpi | 12 hpi | RT-qPCR, CCIF |
| PRRSV | 0.01 FFU | - | - | 48 hpi | 24 hpi | 24 hpi | RT-qPCR, CCIF |
|  | 0.01 FFU | 1 μM | AVA | 48 hpi | 24 hpi | 24 hpi | RT-qPCR, CCIF |
|  | 0.01 FFU | 10 μM | AVA | 48 hpi | 24 hpi | 24 hpi | RT-qPCR, CCIF |
|  | 0.01 FFU | 20 μM | AVA | 48 hpi | 24 hpi | 24 hpi | RT-qPCR, CCIF |
| PSaV | 0.01 FFU | - | - | 36 hpi | - | 36 hpi | RT-qPCR, CCIF |
|  | 0.01 FFU | 1 μM | AVA | 36 hpi | 18 hpi | 18 hpi | RT-qPCR, CCIF |
|  | 0.01 FFU | 10 μM | AVA | 36 hpi | 18 hpi | 18 hpi | RT-qPCR, CCIF |
|  | 0.01 FFU | 20 μM | AVA | 36 hpi | 18 hpi | 18 hpi | RT-qPCR, CCIF |

^a^Abbreviations: IAV, influenza A virus; SARS-CoV-2, severe acute respiratory syndrome coronavirus 2; BCoV, bovine coronavirus; PEDV, porcine epidemic diarrhea coronavirus; RVA, species A rotavirus; PRRSV, porcine reproductive and respiratory syndrome virus; and PSaV, porcine sapovirus; MOI, multiplicity of infection; FFU, fluorescence focus unit; RT-qPCR, reverse transcription quantitative polymerase chain reaction; CCIF, cell culture immunofluorescence assay.

^b^After virus absorption (AVA).

^c^Hour post-infection.

^d^Incubation time after treatment with drug.

**Table S5. Information of cell lines used in this study**

| **Cell line** |  | **Origin** |  | **Source** |  | **Medium** |  | **Supplementation** |
| --- | --- | --- | --- | --- | --- | --- | --- | --- |
| A549 |  | Human lung carcinoma |  | ATCC |  | DMEM |  | 10% FBS, 1% P/S |
| Vero E6 |  | African green monkey kidney epithelium |  | ATCC |  | DMEM |  | 10% FBS, 1% P/S |
| Caco-2 |  | Human colorectal adenocarcinoma |  | ATCC |  | DMEM |  | 10% FBS, 1% P/S |
| HRT-18G |  | Human colorectal adenocarcinoma |  | ATCC |  | DMEM |  | 10% FBS, 1% P/S |
| MA104 |  | Rhesus monkey kidney epithelium |  | ATCC |  | α-MEM |  | 10% FBS, 1% P/S |
| MARC-145 |  | African green monkey kidney epithelium |  | ATCC |  | DMEM |  | 10% FBS, 1% P/S |
| LLC-PK |  | Porcine kidney epithelium |  | ATCC |  | EMEM |  | 10% FBS, 1% P/S |

Abbreviations: ATCC, American Type Culture Collection; FBS, fetal bovine serum; DMEM, Dulbecco’s modified Eagle’s medium; EMEM, Eagle’s Minimum Essential Medium; α-MEM, Minimal Essential Medium alpha modification; 1% P/S, 100 U/mL penicillin and 100 μg/mL streptomycin.

**Table S6. Information of virus strains used in this study**

| **Viruses** |  | **Strain name (genotypes)** |  | **Source** |
| --- | --- | --- | --- | --- |
| Influenza A virus (IAV) |  | Puerto Rico/8 (PR8) (H1N1) |  | American Type Culture Collection |
| Severe acute respiratory syndrome coronavirus 2 (SARS-CoV-2) |  | KCDC03 (Lineage A) |  | Korea Disease Control and Prevention Agency |
|  |  | KDCA51463 (Alpha lineage, British variant) |  | Korea Disease Control and Prevention Agency |
|  |  | KDCA55905 (Beta lineage, South African variant) |  | Korea Disease Control and Prevention Agency |
| Bovine coronavirus (BCoV) |  | KWD20 |  | Isolated from fecal samples and propagated in HRT-18G cells |
| Porcine epidemic diarrhea coronavirus (PEDV) |  | QIAP1401 (G2b) |  | Animal and Plant Quarantine Agency, Korea |
| Bovine species A rotavirus (bovine RVA) |  | NCDV (G6P6[1]) |  | American Type Culture Collection |
| Porcine reproductive and respiratory syndrome virus (PRRSV) |  | LMY (North American type) |  | Animal and Plant Quarantine Agency, Korea |
| Porcine sapovirus (PSaV) |  | Cowden (GIII.1) |  | A kind gift from Dr. K.O. Chang, Kansas State University |

**Table S7. Information for the culture of influenza A virus (IAV), severe acute respiratory syndrome coronavirus 2 (SARS-CoV-2), bovine coronavirus (BCoV), porcine epidemic diarrhea coronavirus (PEDV), bovine species A rotavirus (RVA), porcine reproductive and respiratory syndrome virus (PRRSV), and porcine sapovirus (PSaV)**

| **Viruses** | **Cell lines** | **Activation of viruses** | **Medium** | **Supplementation*** |
| --- | --- | --- | --- | --- |
| IAV | A549 | N/A | DMEM | 1 μg/mL TPCK-treated trypsin, 1% P/S |
| SARS-CoV-2 | Vero E6 | N/A | DMEM | 1 μg/mL TPCK-treated trypsin, 1% P/S |
| BCoV | HRT-18G | N/A | DMEM | 5 μg/mL porcine pancreatin, 1% P/S |
| PEDV | Vero E6 | N/A | EMEM | 3 μg/mL porcine pancreatic trypsin, 1% P/S |
| Bovine RVA | MA104 | Preactivation with 10 μg/mL porcine trypsin | α-MEM | 1 μg/mL crystalized trypsin, 1% P/S |
| PRRSV | MARC-145 | N/A | DMEM | 1% P/S |
| PSaV | LLC-PK | N/A | EMEM | 2.5% FBS, 200 μM GCDCA, 1% P/S |

Abbreviations: FBS, fetal bovine serum; DMEM, Dulbecco’s modified Eagle’s medium; EMEM, Eagle’s Minimum Essential Medium; α-MEM, alpha Minimal Essential Medium; 1% P/S: 100 U/mL penicillin, 100 μg/mL streptomycin; TPCK-treated trypsin, N-tosyl-L-phenylalanine chloromethyl ketone (TPCK)-treated trypsin; GCDCA, glycochenodeoxycholic acid.

**Table S7. Chemicals, kits and siRNAs used in this study**

| **Regents** | **Company** | **City/State** | **Country** | **Solvent** |
| --- | --- | --- | --- | --- |
| **Chemicals** |  |  |  |  |
| TPCK-treated trypsin | Sigma Aldrich | St. Louis, MO | USA | PBS |
| Pancreatin | Gibco | Fort Worth, TX | USA | PBS |
| Porcine pancreatic trypsin | Sigma Aldrich | St. Louis, MO | USA | PBS |
| Crystalized trypsin | Gibco | Fort Worth, TX | USA | PBS |
| GCDCA | Sigma Aldrich | St. Louis, MO | USA | PBS |
| MTT | Sigma Aldrich | St. Louis, MO | USA | PBS |
| Triton X-100 | Sigma Aldrich | St. Louis, MO | USA | PBS |
| Bodipy 493/503 | Sigma Aldrich | St. Louis, MO | USA | PBS |
| Slow-Fade Gold antifade reagent | Molecular Probes | Eugene, OR | USA | - |
| Ultrapure agarose | Thermo Scientific | Waltham, MA | USA | DDW |
| Paraformaldehyde | Sigma Aldrich | St. Louis, MO | USA | - |
| Protein A/G PLUS-Agarose | Santa Cruz | Dallas, TX | USA | - |
| Atglistatin | MedChemExpress | Monmouth Junction, NJ | USA | DMSO |
| CAY10499 | Cayman Chemicals | Ann Arbor, MI | USA | DMSO |
| Remdesivir | Sigma Aldrich | St. Louis, MO | USA | DMSO |
| Corn oil | Sigma Aldrich | St. Louis, MO | USA | DMSO |
| Oseltamivir | Sigma Aldrich | St. Louis, MO | USA | PBS |
| Accutase^TM^ Cell Detachment Solution | BD biosciences | Franklin Lakes, NJ | USA | PBS |
| H89 | Sigma Aldrich | St. Louis, MO | USA | DMSO |
| Palmitic acid | Sigma Aldrich | St. Louis, MO | USA | PBS |
| Oleic acid | Sigma Aldrich | St. Louis, MO | USA | PBS |
| Linoleic acid and | Sigma Aldrich | St. Louis, MO | USA | PBS |
| **Kits** |  |  |  |  |
| Glucose uptake assay kit | Abcam | Cambridge, MA | USA | - |
| Triglyceride colorimetric assay kit | Cayman Chemicals | Ann Arbor, MI | USA | - |
| Cholesterol colorimetric assay kit | Abcam | Cambridge, MA | USA | - |
| Free fatty acid quantification kit | BioVision | Milpitas, CA | USA | - |
| Free glycerol quantification kit | BioVision | Milpitas, CA | USA |  |
| CAPTUREomeTM S-palmitoylated protein kit | Badrilla | Leeds | UK | - |
| RNeasy mini kit | Qiagen | Hilden | Germany | - |
| TOPscript™ cDNA synthesis kit | Enzynomics | Daejeon | South Korea |  |
| In situ cell death detection kit | Roche | Basel | Switzerland | - |
| cAMP detection ELISA kit | BioVision | Milpitas, CA | USA | - |
| IFN alpha ELISA kit | Thermo Scientific | Waltham, MA | USA | - |
| IFN beta ELISA kit | Thermo Scientific | Waltham, MA | USA | - |
| IL-6 ELISA kit | Thermo Scientific | Waltham, MA | USA | - |
| TNF alpha ELISA kit | Thermo Scientific | Waltham, MA | USA | - |
| MCP-1 EIISA kit | Thermo Scientific | Waltham, MA | USA | - |
| Fatty Acid Oxidation Assay kit | Assay Genie | Dublin | Ireland | - |
| Seahorse XF Palmitate Oxidation Stress Test Kit | Agilent Technologies | Santa Clara, CA | USA | - |
| **siRNAs** |  |  |  |  |
| ON-TARGETplus siRNAs against PLIN3, ATGL, HSL (set of 4) | GE healthcare Dharmacon | Lafayette, Colorado | USA | RNAse free water |
| Lipofectamine3000 | Thermo Scientific | Waltham, MA | USA | - |

**Table S8. Antibodies used in this study**

| **Antibodies*** | **Host** | **Type*** | **Conjugation** | **Company** | **City/State** | **Country** | **Solvent*** |
| --- | --- | --- | --- | --- | --- | --- | --- |
| **Primary antibodies** |  |  |  |  |  |  |  |
| HSL | Rabbit | Pab | - | Abcam | Cambridge | UK | TBST (5% BSA) |
| pHSL(S563) | Rabbit | Pab | - | Cell Signaling | Beverly, MA | USA | TBST (5% BSA) |
| pHSL(S660) | Rabbit | Pab | - | Cell Signaling | Beverly, MA | USA | TBST (5% BSA) |
| ATGL | Rabbit | Pab | - | Cell Signaling | Beverly, MA | USA | TBST (5% BSA) |
| PLIN3 | Mouse | Mab |  | Santa Cruz | Dallas, TX, | USA | TBST (5% BSA) |
| MLKL | Rabbit | Pab |  | Abcam | Cambridge | UK | TBST (5% BSA) |
| pMLKL | Rabbit | Pab | - | Cell Signaling | Beverly, MA | USA | TBST (5% BSA) |
| Cas-3 | Rabbit | Pab | - | Cell Signaling | Beverly, MA | USA | TBST (5% BSA) |
| SARS-CoV-2 S | Rabbit | Pab | - | Prosci | San Diego, CA | USA | TBST (5% BSA) |
| SARS-CoV-2 N | Mouse | Mab | - | Prosci | San Diego, CA | USA | TBST (5% BSA) |
| IAV M2 | Mouse | Mab | - | Abcam | Cambridge | UK | TBST (5% BSA) |
| IAV NP | Mouse | Mab | - | Abcam | Cambridge | UK | TBST (5% BSA) |
| IAV HA | Mouse | Mab | - | Santa Cruz | Dallas, TX, | USA | TBST (5% BSA) |
| IAV virion | Goat | Pab | - | Viasat | Carlsbad, CA | USA | TBST (5% BSA) |
| GAPDH | Mouse | Mab |  | Santa Cruz | Dallas, TX, | USA | TBST (5% BSA) |
| β-actin | Mouse | Mab | - | Thermo Scientific | Waltham, MA | USA | TBST (5% BSA) |
| BCoV S | Mouse | Mab | - | Native Antigen Company | Killington | UK | TBST (5% BSA) |
| PEDV N | Mouse | Mab | - | Medgene Labs | Brookings, SD | USA | TBST (5% BSA) |
| RVA VP6 | Mouse | Mab | - | Median Diagnostic | Chuncheon | South Korea | TBST (5% BSA) |
| PRRSV M | Rabbit | Pab | - | Bioss Antibodies | Woburn, MA | USA | TBST (5% BSA) |
| PSaV VPg | Rabbit | Antiserum | - | Manufactured in the lab^a^ |  |  | TBST (5% BSA) |
| PKA C-α | Rabbit | Pab | - | Cell Signaling | Beverly, MA | USA | TBST (5% BSA) |
| pPKA C (Thr 197) | Rabbit | Mab | - | Cell Signaling | Beverly, MA | USA | TBST (5% BSA) |
| **Secondary antibodies** | | | | | | | |
| Rabbit IgG(H+L) | Goat | Pab | HRP | Cell Signaling | Beverly, MA | USA | 1xTBST |
| Goat IgG(H+L) | Rabbit | Pab | HRP | Cell Signaling | Beverly, MA | USA | 1xTBST |
| Mouse IgGκ | Goat | Mab | HRP | Santa Cruz | Dallas, TX | USA | 1xTBST |
| AF647 rabbit IgG | Donkey | Pab | AF647 | Thermo Scientific | Waltham, MA | USA | 1xPBS |
| AF594 rabbit IgG | Donkey | Pab | AF594 | Thermo Scientific | Waltham, MA | USA | 1xPBS |
| AF488 rabbit IgG | Donkey | Pab | AF488 | Thermo Scientific | Waltham, MA | USA | 1xPBS |
| AF647 mouse IgG | Goat | Pab | AF647 | Thermo Scientific | Waltham, MA | USA | 1xPBS |
| AF594 mouse IgG | Goat | Pab | AF594 | Thermo Scientific | Waltham, MA | USA | 1xPBS |
| AF488 mouse IgG | Goat | Pab | AF488 | Thermo Scientific | Waltham, MA | USA | 1xPBS |
| DyLight594-Goat IgG | Donkey | Pab | DyLight594 | Bethyl Laboratories | Montgomery, TX | USA | 1xPBS |

*Abbreviation: HSL, hormone-sensitive lipase; ATGL, Adipose triglyceride lipase; PLIN 3, perilipin 3; MLKL, mixed lineage kinase domain-like protein; Cas-3, caspase 3; SARS-CoV-2 S, SARS-CoV-2 spike; SARS-CoV-2 N, SARS-CoV-2 nucleocapsid; M2, membrane; NP, nucleoprotein; HA, hemagglutinin; BCoV S, BCoV spike protein; PEDV N, PEDV nucleoprotein; RVA VP6, viral structural protein 6; PRRSV M, PRRSV matrix protein; PSaV VPg, PSaV viral genome-linked protein; PKA C-α, protein kinase A C-subunit α isoform; pPKA C, phosphorylated PKA C-subunit; Mab or Pab: monoclonal or polyclonal antibodies; HRP: horseradish peroxidase; 1xTBST; 1x Tris-Buffered Saline containing 0.1% Tween® 20 Detergent; TBST (5% BSA); 1x TBST containing 5% BSA (Bovine Serum Albumin)

^a^Generated by immunization of a New Zealand White rabbit with purified PSaV VPg.^9^

**Table S9. Experimental design for determining the distribution of atglistatin and CAY10499 in the lung and blood samples in mice and hamsters**

| Group | Animal | | |  | Treatment | | | | Ex. Period  (hour) | Samples |
| --- | --- | --- | --- | --- | --- | --- | --- | --- | --- | --- |
|  | Breed | Age | No. |  | Chemical^a^ | Dosage | Route | Administration |  |  |
| 1 | C57BL/6J | 8 wk | 4 |  | Atglistatin | 10 mg/kg/day | IP^b^ | Bid^c^ for 1 day | 12 | Blood^d^/lung |
| 2 | C57BL/6J | 8 wk | 4 |  | CAY10499 | 10 mg/kg/day | IP | Bid for 1 day | 12 | Blood/lung |
| 3 | Syrian | 12 wk | 4 |  | Atglistatin | 80 mg/kg/day | IP | Bid for 1 day | 12 | Blood/lung |

^a^Vehicles for mice and hamsters, 10% and 50% PEG400 in water (v/v), respectively.

^b^IP, intraperitoneal administration.

^c^Bid, twice daily with a 6-h interval.

^d^Blood sample was collected from abdominal caudal vena cava.

**Table S10. Experimental design for determining organ-specific toxicity of atglistatin and CAY10499 in the mice and hamsters**

| Group | Animal | | |  | Treatment | | | | Ex. Period (day) |
| --- | --- | --- | --- | --- | --- | --- | --- | --- | --- |
|  | Breed | Age | No. |  | Chemical | Dosage | Route | Administration |  |
| 1 | C57BL/6J | 8 wk | 3 |  | Vehicle^a^ | None | IP^b^ | Bid^c^ for 4 days | 5 |
| 2 | C57BL/6J | 8 wk | 3 |  | Atglistatin | 0.1 mg/kg/day | IP | Bid for 4 days | 5 |
| 3 | C57BL/6J | 8 wk | 3 |  | Atglistatin | 1 mg/kg/day | IP | Bid for 4 days | 5 |
| 4 | C57BL/6J | 8 wk | 3 |  | Atglistatin | 10 mg/kg/day | IP | Bid for 4 days | 5 |
| 5 | C57BL/6J | 8 wk | 3 |  | CAY10499 | 0.1 mg/kg/day | IP | Bid for 4 days | 5 |
| 6 | C57BL/6J | 8 wk | 3 |  | CAY10499 | 1 mg/kg/day | IP | Bid for 4 days | 5 |
| 7 | C57BL/6J | 8 wk | 3 |  | CAY10499 | 10 mg/kg/day | IP | Bid for 4 days | 5 |
| 8 | Syrian | 12 wk | 3 |  | Vehicle^a^ | None | IP^b^ | Bid^c^ for 4.5 days | 5 |
| 9 | Syrian | 12 wk | 3 |  | Atglistatin | 20 mg/kg/day | IP | Bid for 4.5 days | 5 |
| 10 | Syrian | 12 wk | 3 |  | Atglistatin | 40 mg/kg/day | IP | Bid for 4.5 days | 5 |
| 11 | Syrian | 12 wk | 3 |  | Atglistatin | 80 mg/kg/day | IP | Bid for 4.5 days | 5 |

^a^Vehicles for mice and hamsters, 10% and 50% PEG400 in water (v/v), respectively.

^b^IP, intraperitoneal administration.

^c^Bid, twice daily with a 12-h interval.

**Table S11. Experimental design for determining antiviral effects of atglistatin on influenza A virus infection in the mouse model**

| Group | Animal | | |  | Inoculum | | |  | Treatment | | | | Ex. Period (day) |
| --- | --- | --- | --- | --- | --- | --- | --- | --- | --- | --- | --- | --- | --- |
|  | Breed | Age | No. |  | Strain | Titer | Route |  | Chemical | Dosage | Route | Administration |  |
| 1 | C57BL/6J | 8 wk | 16 |  | PBS | None | IN^a^ |  | Vehicle^b^ | None | IP^c^ | Bid^d^ for 4 days | 15 |
| 2 | C57BL/6J | 8 wk | 16 |  | PR8 | 10^3^ PFU | IN |  | Vehicle | None | IP | Bid for 4 days | 15 |
| 3 | C57BL/6J | 8 wk | 16 |  | PR8 | 10^3^ PFU | IN |  | Atglistatin | 0.01 mg/kg/day | IP | Bid for 4 days | 15 |
| 4 | C57BL/6J | 8 wk | 16 |  | PR8 | 10^3^ PFU | IN |  | Atglistatin | 0.1 mg/kg/day | IP | Bid for 4 days | 15 |
| 5 | C57BL/6J | 8 wk | 16 |  | PR8 | 10^3^ PFU | IN |  | Atglistatin | 1 mg/kg/day | IP | Bid for 4 days | 15 |
| 6 | C57BL/6J | 8 wk | 16 |  | PR8 | 10^3^ PFU | IN |  | Atglistatin | 5 mg/kg/day | IP | Bid for 4 days | 15 |
| 7 | C57BL/6J | 8 wk | 16 |  | PR8 | 10^3^ PFU | IN |  | Atglistatin | 10 mg/kg/day | IP | Bid for 4 days | 15 |

^a^IN, intranasal inoculation.

^b^Vehicle, 10% PEG400 in water (v/v).

^c^IP, intraperitoneal administration.

^d^Bid, twice daily with a 12-h interval.

**Table S12. Experimental design for determining antiviral effects of CAY10499 on influenza A virus infection in the mouse model**

| Group | Animal | | |  | Inoculum | | |  | Treatment | | | | Ex. Period (day) |
| --- | --- | --- | --- | --- | --- | --- | --- | --- | --- | --- | --- | --- | --- |
|  | Breed | Age | No. |  | Strain | Titer | Route |  | Chemical | Dosage | Route | Administration |  |
| 1 | C57BL/6J | 8 wk | 16 |  | PBS | None | IN^a^ |  | Vehicle^b^ | None | IP^c^ | Bid^d^ for 4 days | 15 |
| 2 | C57BL/6J | 8 wk | 16 |  | PR8 | 10^3^ PFU | IN |  | Vehicle | None | IP | Bid for 4 days | 15 |
| 3 | C57BL/6J | 8 wk | 16 |  | PR8 | 10^3^ PFU | IN |  | CAY10499 | 0.01 mg/kg/day | IP | Bid for 4 days | 15 |
| 4 | C57BL/6J | 8 wk | 16 |  | PR8 | 10^3^ PFU | IN |  | CAY10499 | 0.1 mg/kg/day | IP | Bid for 4 days | 15 |
| 5 | C57BL/6J | 8 wk | 16 |  | PR8 | 10^3^ PFU | IN |  | CAY10499 | 1 mg/kg/day | IP | Bid for 4 days | 15 |
| 6 | C57BL/6J | 8 wk | 16 |  | PR8 | 10^3^ PFU | IN |  | CAY10499 | 5 mg/kg/day | IP | Bid for 4 days | 15 |
| 7 | C57BL/6J | 8 wk | 16 |  | PR8 | 10^3^ PFU | IN |  | CAY10499 | 10 mg/kg/day | IP | Bid for 4 days | 15 |

^a^IN, intranasal inoculation.

^b^Vehicle, 10% PEG400 in water (v/v).

^c^IP, intraperitoneal administration.

^d^Bid, twice daily with a 12-h interval.

**Table S13. Experimental design for determining antiviral effects of atglistatin on infection of SARS-CoV-2 KCDC03 strain in the Syrian hamster model**

| Group | Animal | | |  | Inoculum | | |  | Treatment | | | | Ex. Period (day) |
| --- | --- | --- | --- | --- | --- | --- | --- | --- | --- | --- | --- | --- | --- |
|  | Breed | Age | No. |  | Strain | Titer | Route |  | Chemical | Dosage | Route | Administration |  |
| 1 | Syrian | 12 wk | 5 |  | PBS | None | IT^a^ |  | Vehicle^b^ | None | IP^c^ | Bid^d^ for 4.5 days | 5 |
| 2 | Syrian | 12 wk | 5 |  | KCDC03 | 10^5^ TCID_50_ | IT |  | Vehicle | None | IP | Bid for 4.5 days | 5 |
| 3 | Syrian | 12 wk | 5 |  | KCDC03 | 10^5^ TCID_50_ | IT |  | Atglistatin | 20 mg/kg/day | IP | Bid for 4.5 days | 5 |
| 4 | Syrian | 12 wk | 5 |  | KCDC03 | 10^5^ TCID_50_ | IT |  | Atglistatin | 40 mg/kg/day | IP | Bid for 4.5 days | 5 |
| 5 | Syrian | 12 wk | 5 |  | KCDC03 | 10^5^ TCID_50_ | IT |  | Atglistatin | 80 mg/kg/day | IP | Bid for 4.5 days | 5 |

^a^IT, intratracheal inoculation.

^b^Vehicle, 50% PEG400 in water (v/v).

^c^IP, intraperitoneal administration.

^d^Bid, twice daily with a 12-h interval.

**Table S14. Experimental design for determining combination therapy of atglistatin and oseltamivir on influenza A virus infection in the mouse model**

| Group | Animal | | |  | Inoculum | | |  | Treatment | | | | Ex. Period (day) |
| --- | --- | --- | --- | --- | --- | --- | --- | --- | --- | --- | --- | --- | --- |
|  | Breed | Age | No. |  | Strain | Titer | Route |  | Chemical | Dosage | Route | Administration |  |
| 1 | C57BL/6J | 8 wk | 16 |  | PBS | None | IN^a^ |  | Vehicle^b^ | None | IP^c^ | Bid^d^ for 4 days | 15 |
| 2 | C57BL/6J | 8 wk | 16 |  | PR8 | 10^3^ PFU | IN |  | Vehicle | None | IP | Bid for 4 days | 15 |
| 3 | C57BL/6J | 8 wk | 16 |  | PR8 | 10^3^ PFU | IN |  | Oseltamivir | 2 mg/kg/day | Oral | Bid for 4 days | 15 |
| 4 | C57BL/6J | 8 wk | 16 |  | PR8 | 10^3^ PFU | IN |  | Atglistatin | 5 mg/kg/day | IP | Bid for 4 days | 15 |
| 5 | C57BL/6J | 8 wk | 16 |  | PR8 | 10^3^ PFU | IN |  | Atglistatin | 5 mg/kg/day | IP | Bid for 4 days | 15 |
|  |  |  |  |  |  |  |  |  | Oseltamivir | 2 mg/kg/day | Oral |  |  |

^a^IN, intranasal inoculation.

^b^Vehicle, 10% PEG400 in water (v/v).

^c^IP, intraperitoneal administration.

^d^Bid, twice daily with a 12-h interval.

**Table S15. Experimental design for determining combination therapy of combination therapy of CAY10499 and oseltamivir on influenza A virus infection in the mouse model**

| Group | Animal | | |  | Inoculum | | |  | Treatment | | | | Ex. Period (day) |
| --- | --- | --- | --- | --- | --- | --- | --- | --- | --- | --- | --- | --- | --- |
|  | Breed | Age | No. |  | Strain | Titer | Route |  | Chemical | Dosage | Route | Administration |  |
| 1 | C57BL/6J | 8 wk | 16 |  | PBS | None | IN^a^ |  | Vehicle^b^ | None | IP^c^ | Bid^d^ for 4 days | 15 |
| 2 | C57BL/6J | 8 wk | 16 |  | PR8 | 10^3^ PFU | IN |  | Vehicle | None | IP | Bid for 4 days | 15 |
| 3 | C57BL/6J | 8 wk | 16 |  | PR8 | 10^3^ PFU | IN |  | Oseltamivir | 2 mg/kg/day | Oral | Bid for 4 days | 15 |
| 4 | C57BL/6J | 8 wk | 16 |  | PR8 | 10^3^ PFU | IN |  | CAY10499 | 5 mg/kg/day | IP | Bid for 4 days | 15 |
| 5 | C57BL/6J | 8 wk | 16 |  | PR8 | 10^3^ PFU | IN |  | CAY10499 | 5 mg/kg/day | IP | Bid for 4 days | 15 |
|  |  |  |  |  |  |  |  |  | Oseltamivir | 2 mg/kg/day | Oral |  |  |

^a^IN, intranasal inoculation.

^b^Vehicle, 10% PEG400 in water (v/v).

^c^IP, intraperitoneal administration.

^d^Bid: twice daily with a 12-h interval.

**Table S16. Experimental design for determining combination therapy of atglistatin and remdesivir on infection of SARS-CoV-2 KCDC03 strain (lineage A) in the Syrian hamster model**

| Group | Animal | | |  | Inoculum | | |  | Treatment | | | | Ex. Period (day) |
| --- | --- | --- | --- | --- | --- | --- | --- | --- | --- | --- | --- | --- | --- |
|  | Breed | Age | No. |  | Strain | Titer | Route |  | Chemical | Dosage | Route | Administration |  |
| 1 | Syrian | 12 wk | 5 |  | PBS | None | IT^a^ |  | Vehicle^b^ | None | IP^c^ | Bid^d^ for 4.5 days | 5 |
| 2 | Syrian | 12 wk | 5 |  | KCDC03 | 10^5^ TCID_50_ | IT |  | Vehicle | None | IP | Bid for 4.5 days | 5 |
| 3 | Syrian | 12 wk | 5 |  | KCDC03 | 10^5^ TCID_50_ | IT |  | Remdesivir | 2.5 mg/Kg/day | IP | Bid for 4.5 days | 5 |
| 4 | Syrian | 12 wk | 5 |  | KCDC03 | 10^5^ TCID_50_ | IT |  | Atglistatin | 40 mg/Kg/day | IP | Bid for 4.5 days | 5 |
| 5 | Syrian | 12 wk | 5 |  | KCDC03 | 10^5^ TCID_50_ | IT |  | Atglistatin | 40 mg/Kg/day | IP | Bid for 4.5 days | 5 |
|  |  |  |  |  |  |  |  |  | Remdesivir | 2.5 mg/Kg/day |  |  |  |

^a^IT, intratracheal inoculation.

^b^Vehicle, 50% PEG400 in water (v/v).

^c^IP, intraperitoneal administration.

^d^Bid, twice daily with a 12-h interval.

**Table S17. Experimental design for determining combination therapy of atglistatin and remdesivir on infection of SARS-CoV-2** **KDCA51463 strain (Alpha lineage, British variant) in the Syrian hamster model**

| Group | Animal | | |  | Inoculum | | |  | Treatment | | | | Ex. Period (day) |
| --- | --- | --- | --- | --- | --- | --- | --- | --- | --- | --- | --- | --- | --- |
|  | Breed | Age | No. |  | Strain | Titer | Route |  | Chemical | Dosage | Route | Administration |  |
| 1 | Syrian | 12 wk | 5 |  | PBS | None | IT^a^ |  | Vehicle^b^ | None | IP^c^ | Bid^d^ for 4.5 days | 5 |
| 2 | Syrian | 12 wk | 5 |  | KDCA51463 | 10^5^ TCID_50_ | IT |  | Vehicle | None | IP | Bid for 4.5 days | 5 |
| 3 | Syrian | 12 wk | 5 |  | KDCA51463 | 10^5^ TCID_50_ | IT |  | Remdesivir | 2.5 mg/Kg/day | IP | Bid for 4.5 days | 5 |
| 4 | Syrian | 12 wk | 5 |  | KDCA51463 | 10^5^ TCID_50_ | IT |  | Atglistatin | 40 mg/Kg/day | IP | Bid for 4.5 days | 5 |
| 5 | Syrian | 12 wk | 5 |  | KDCA51463 | 10^5^ TCID_50_ | IT |  | Atglistatin | 40 mg/Kg/day | IP | Bid for 4.5 days | 5 |
|  |  |  |  |  |  |  |  |  | Remdesivir | 2.5 mg/Kg/day |  |  |  |

^a^IT, intratracheal inoculation.

^b^Vehicle, 50% PEG400 in water (v/v).

^c^IP, intraperitoneal administration.

^d^Bid, twice daily with a 12-h interval.

**Table S18. Experimental design for determining combination therapy of atglistatin and remdesivir on infection of SARS-CoV-2 KDCA55905 strain (Beta lineage, South African variant) in the Syrian hamster model**

| Group | Animal | | |  | Inoculum | | |  | Treatment | | | | Ex. Period (day) |
| --- | --- | --- | --- | --- | --- | --- | --- | --- | --- | --- | --- | --- | --- |
|  | Breed | Age | No. |  | Strain | Titer | Route |  | Chemical | Dosage | Route | Administration |  |
| 1 | Syrian | 12 wk | 5 |  | PBS | None | IT^a^ |  | Vehicle^b^ | None | IP^c^ | Bid^d^ for 4.5 days | 5 |
| 2 | Syrian | 12 wk | 5 |  | KDCA55905 | 10^5^ TCID_50_ | IT |  | Vehicle | None | IP | Bid for 4.5 days | 5 |
| 3 | Syrian | 12 wk | 5 |  | KDCA55905 | 10^5^ TCID_50_ | IT |  | Remdesivir | 2.5 mg/Kg/day | IP | Bid for 4.5 days | 5 |
| 4 | Syrian | 12 wk | 5 |  | KDCA55905 | 10^5^ TCID_50_ | IT |  | Atglistatin | 40 mg/Kg/day | IP | Bid for 4.5 days | 5 |
| 5 | Syrian | 12 wk | 5 |  | KDCA55905 | 10^5^ TCID_50_ | IT |  | Atglistatin | 40 mg/Kg/day | IP | Bid for 4.5 days | 5 |
|  |  |  |  |  |  |  |  |  | Remdesivir | 2.5 mg/Kg/day |  |  |  |

^a^IT, intratracheal inoculation.

^b^Vehicle, 50% PEG400 in water (v/v).

^c^IP, intraperitoneal administration.

^d^Bid: twice daily with a 12-h interval.

**Table S19. Experimental design for determining inhibitory effects of atglistatin and CAY10499 on generation of fatty acids and glycerol, virus replication, and histopathological lesions in the lungs from IAV-infected mice**

| Group | Animal | | |  | Inoculum | | |  | Treatment | | | | Ex. Period (day) |
| --- | --- | --- | --- | --- | --- | --- | --- | --- | --- | --- | --- | --- | --- |
|  | Breed | Age | No. |  | Strain | Titer | Route |  | Chemical | Dosage | Route | Administration |  |
| 1 | C57BL/6J | 8 wk | 4 |  | PBS | None | IN^a^ |  | Vehicle^b^ | None | IP^c^ | Bid^d^ for 4 days | 6 |
| 2 | C57BL/6J | 8 wk | 4 |  | PR8 | 10^3^ PFU | IN |  | Vehicle | None | IP | Bid for 4 days | 6 |
| 3 | C57BL/6J | 8 wk | 4 |  | PR8 | 10^3^ PFU | IN |  | Atglistatin | 5 mg/Kg/day | IP | Bid for 4 days | 6 |
| 4 | C57BL/6J | 8 wk | 4 |  | PR8 | 10^3^ PFU | IN |  | CAY10499 | 5 mg/Kg/day | IP | Bid for 4 days | 6 |

^a^IN: intranasal inoculation.

^b^Vehicle, 10% PEG400 in water (v/v).

^c^IP, intraperitoneal administration.

^d^Bid, twice daily with a 12-h interval.

**Table S20. Experimental design for determining inhibitory effects of atglistatin and CAY10499 on spread of IAV through viremia in IAV-infected mice**

| Group | Animal | | |  | Inoculum | | |  | Treatment | | | | Ex. Period (day) |
| --- | --- | --- | --- | --- | --- | --- | --- | --- | --- | --- | --- | --- | --- |
|  | Breed | Age | No. |  | Strain | Titer | Route |  | Chemical | Dosage | Route | Administration |  |
| 1 | C57BL/6J | 8 wk | 3 |  | PBS | None | IN^a^ |  | Vehicle^b^ | None | IP^c^ | Bid^d^ for 4 days | 4 |
| 2 | C57BL/6J | 8 wk | 3 |  | PR8 | 10^3^ PFU | IN |  | Vehicle | None | IP | Bid for 4 days | 4 |
| 3 | C57BL/6J | 8 wk | 3 |  | PR8 | 10^3^ PFU | IN |  | Atglistatin | 5 mg/Kg/day | IP | Bid for 4 days | 4 |
| 4 | C57BL/6J | 8 wk | 3 |  | PR8 | 10^3^ PFU | IN |  | CAY10499 | 5 mg/Kg/day | IP | Bid for 4 days | 4 |

^a^IN: intranasal inoculation.

^b^Vehicle, 10% PEG400 in water (v/v).

^c^IP, intraperitoneal administration.

^d^Bid, twice daily with a 12-h interval.

**Table S21. Experimental design for determining inhibitory effects of atglistatin on generation of fatty acids and glycerol, virus replication, and histopathological lesions in the lungs from SARS-CoV-2-infected hamsters**

| Group | Animal | | |  | Inoculum | | |  | Treatment | | | | Ex. Period (day) |
| --- | --- | --- | --- | --- | --- | --- | --- | --- | --- | --- | --- | --- | --- |
|  | Breed | Age | No. |  | Strain | Titer | Route |  | Chemical | Dosage | Route | Administration |  |
| 1 | Syrian | 12 wk | 5 |  | PBS | None | IT^a^ |  | Vehicle^b^ | None | IP^c^ | Bid^d^ for 4.5 days | 5 |
| 2 | Syrian | 12 wk | 5 |  | KCDC03 | 10^5^ TCID_50_ | IT |  | Vehicle | None | IP | Bid for 4.5 days | 5 |
| 3 | Syrian | 12 wk | 5 |  | KCDC03 | 10^5^ TCID_50_ | IT |  | Atglistatin | 20 mg/kg/day | IP | Bid for 4.5 days | 5 |
| 4 | Syrian | 12 wk | 5 |  | KCDC03 | 10^5^ TCID_50_ | IT |  | Atglistatin | 40 mg/kg/day | IP | Bid for 4.5 days | 5 |
| 5 | Syrian | 12 wk | 5 |  | KCDC03 | 10^5^ TCID_50_ | IT |  | Atglistatin | 80 mg/kg/day | IP | Bid for 4.5 days | 5 |

^a^IT, intratracheal inoculation.

^b^Vehicle, 50% PEG400 in water (v/v).

^c^IP, intraperitoneal administration.

^d^Bid, twice daily with a 12-h interval.

**Table S22. Primers used for the detection of viral and host genes**

| **Name**^a^ | **Sequence** | **Size of amplicon** | **Reference number** |
| --- | --- | --- | --- |
| IAV PB1 | F: 5’-CTGCCAGAAGACAATGAACC-3’ | 66 | 36 |
|  | R: 5’-GGCCATTGCTTCCAATACAC-3’ |  |  |
| SARS-CoV-2 N | F: 5’-TAATCAGACAAGGAACTGATTA-3’ | 109 | 3 |
|  | R: 5’-CGAAGGTGTGACTTCCATG’ -3’ |  |  |
| RVA VP6 | F: 5’-TAG ACC AAA TAA CGT TGA AGT TGA-3’ | 236 | 37 |
|  | R: 5’-GAT TCA CAA ACT GCA GAT TCA A-3’ |  |  |
| PEDV N | F: 5’-GCTATGCTCAGATCGCCAGT-3’ | 92 | 38 |
|  | R: 5’-TCTCGTAAGAGTCCGCTAGCTC-3’ |  |  |
| PRRSV M | F: 5’-CACCTCCAGATGCCGTTTG-3’ | 113 | 39 |
|  | R: 5’-ATGCGTGGTTATCATTTGCC-3’ |  |  |
| PSaV VPg | F: 5’-CG AAA GGG AAA AAC AAA CGC-3’ | 239 | 9 |
|  | R: 5’-TCACTCACTGTC ATA GGTGTCACC-3’ |  |  |
| BCoV N | F: 5’-TGG ATC AAG ATT AGA GTT GGC-3’ | 236 | 40 |
|  | R: 5’-CCT TGT CCA TTC TTC TGA CC-3’ |  |  |
| L32 | F: 5’-TCTGGTGAAGCCCAAGATCG-3’ | 101 | 41 |
|  | R: 5’-CTCTGGGTTTCCGCCAGT-3’ |  |  |
| Human GAPDH | F: 5’-ATTCCACCCATGGCAAATTC-3’ | 90 | 42 |
|  | R: 5’-CGCTCCTGGAAGATGGTGAT-3’ |  |  |
| Mouse GAPDH | F: 5’-AAGGTCATCCCAGAGCTGAA-3’ | 137 | 43 |
|  | R: 5’-CTGCTTCACCACCTTCTTGA-3’ |  |  |
| Porcine GAPDH | F: 5’-ACCTCCACTACATGGTCTACA-3’ | 90 | 44 |
|  | R: 5’-ATGACAAGCTTCCCGTTCTC-3’ |  |  |
| Mouse IFN-α | F: 5’-TAC TCA GCA GAC CTT GAA CCT-3’ | 307 | 45 |
|  | R: 5’-CAG TCT TGG CAG CAA GTT GAC-3’ |  |  |
| Mouse IFN-β | F: 5’-CAG CTC CAA GAA AGG ACG AAC-3’ | 138 | 45 |
|  | R: 5’-GGC AGT GTA ACT CTT CTG CAT-3’ |  |  |
| Mouse IL-6 | F: 5’-GCT ACC AAA CTG GAT ATA ATC AGG A-3’ | 78 | 46 |
|  | R: 5’-CCA GGT AGC TAT GGT ACT CCA GAA-3’ |  |  |
| Mouse TNF-α | F: 5’-GCCTCTTCTCATTCCTGCTTG-3’ | 115 | 47 |
|  | R: 5’-CTGATGAGAGGGAGGCCATT-3’ |  |  |
| Mouse MCP-1 | F: 5’-GCT ACA AGA GGA TCA CCA GCA G -3’ | 106 | 48 |
|  | R: 5’-GTC TGG ACC CAT TCC TTC TTG G-3’ |  |  |
| Hamster IFN-α | F: 5’-GCC TCT ACC AGC AGC TCA GT-3’ | 199 | 49 |
|  | R: 5’-GCA GAC AGG GTT CTC CAG AC-3’ |  |  |
| Hamster IFN-β | F: 5’-TTG TGC TTC TCC ACT ACA GC-3’ | 91 | 50 |
|  | R: 5’-GTG TCT AGA TCT GAC AAC CT-3’ |  |  |
| Hamster IL-6 | F: 5’-CTC CGC AAG AGA CTT CCA TC-3’ | 156 | 49 |
|  | R: 5’-ACC AAA CCT CCG ACT TGT TG-3’ |  |  |
| Hamster TNF-α | F: 5’-GAC GGG CTG TAC CTG GTT TA-3’ | 237 | 49 |
|  | R: 5’-GAG TCG GTC ACC TTT CTC CA-3’ |  |  |
| Hamster MCP-1 | F: 5’- TCCTGCAAGTCAATCCTGCC -3’ | 218 | 51 |
|  | R: 5’- GAAGTGATGGAGAGACGGGC -3’ |  |  |
| Human IFN-α | F: 5’-CTT GAA GGA CAG ACA TGA CTT TGG A-3’ | 107 | 52 |
|  | R: 5’-GGA TGG TTT CAG CCT TTT GGA-3’ |  |  |
| Human IFN-β | F: 5’-GCT TGG ATT CCT ACA AAG AAG CA-3’ | 166 | 52 |
|  | R: 5’-ATA GAT GGT CAA TGC GGC GTC-3’ |  |  |
| Human IL-6 | F: 5’-ACT CAC CTC TTC AGA ACG AAT TG-3’ | 149 | 53 |
|  | R: 5’-CCA TCT TTG GAA GGT TCA GGT TG-3’ |  |  |
| Human TNF-α | F: 5’-CCT CTC TCT AAT CAG CCC TCT G-3’ | 220 | 52 |
|  | R: 5’-GAG GAC CTG GGA GTA GAT GAG-3’ |  |  |
| Human MCP-1 | F: 5’-CAG CCA GAT GCA ATC AAT GCC-3’ | 190 | 52 |
|  | R: 5’-TGG AAT CCT GAA CCC ACT TCT-3’ |  |  |

*Abbreviations: IAV PB1, influenza A virus RNA-directed RNA polymerase catalytic subunit gene; SARS-CoV-2 N, severe acute respiratory syndrome coronavirus 2 nucleocapsid protein gene; RVA VP6, species A rotavirus VP6 gene; PEDV N, porcine epidemic diarrhea coronavirus nucleoprotein gene; PRRSV M, porcine reproductive and respiratory syndrome virus matrix protein gene; PSaV VPg, porcine sapovirus VPg; BCoV N, bovine coronavirus nucleocapsid protein gene; L32, 60S ribosomal protein gene; Human GAPDH, human glyceraldehyde 3-phosphate dehydrogenase gene; Mouse GAPDH, mouse glyceraldehyde 3-phosphate dehydrogenase gene; Porcine GAPDH, porcine glyceraldehyde 3-phosphate dehydrogenase gene; IFN-α, interferon alpha; IFN-β, IFN beta; IL-6, interleukin 6; TNF-α, tumor necrosis alpha, MCP-1, monocyte chemoattractant protein-1.

**References**

1. Xue, J., Chambers, B. S., Hensley, S. E. & López, C. B. Propagation and characterization of influenza virus stocks that lack high levels of defective viral genomes and hemagglutinin mutations. *Front. Microbiol.* **7**, 326 (2016).
2. Konings, F. et al. SARS-CoV-2 variants of interest and concern naming scheme conducive for global discourse. *Nat. Microbiol.* **6**, 821-823 (2021).
3. Ogando, N. S. et al. SARS-coronavirus-2 replication in Vero E6 cells: replication kinetics, rapid adaptation and cytopathology. *J. Gen. Virol.* **101**, 925-940 (2020).
4. Park, S. J. et al. Detection and characterization of bovine coronaviruses in fecal specimens of adult cattle with diarrhea during the warmer seasons. *J. Clin. Microbiol.* **44**, 3178-3188 (2006).
5. Kim, H. K. et al. Genetic analysis of ORF5 of recent Korean porcine reproductive and respiratory syndrome viruses (PRRSVs) in viremic sera collected from MLV-vaccinating or non-vaccinating farms. *J. Vet. Sci.* **10**, 121-130 (2009).
6. Lee, S., Kim, Y. & Lee, C. Isolation and characterization of a Korean porcine epidemic diarrhea virus strain KNU-141112. *Virus Res.* **208**, 215-224 (2015).
7. Kim, H. J. et al. Detection and genotyping of Korean porcine rotaviruses. *Vet. Microbiol.* **144**, 274-286 (2010).
8. Chang, K. O. et al. Bile acids are essential for porcine enteric calicivirus replication in association with down-regulation of signal transducer and activator of transcription 1. *Proc. Natl. Acad. Sci. U S A* **101**, 8733-8738 (2004).
9. Alfajaro, M. M. et al. Early porcine spovirus infection disrupts tight junctions and uses occludin as a coreceptor. *J. Virol.* **93**, e01773-18 (2019).
10. van Meerloo, J., Kaspers, G. J. & Cloos, J. Cell sensitivity assays: the MTT assay. *Methods Mol. Biol.* **731**, 237-245 (2011).
11. Zhang, B. et al. G protein alpha S subunit promotes cell proliferation of renal cell carcinoma with involvement of protein kinase A signaling. *DNA Cell Biol.* **36**, 237-242 (2017).
12. Sasser, M. Identification of bacteria by gas chromatography of cellular fatty acids. MIDI technical note 101 (1990). Newark, DE: MIDI inc.
13. Song, Y. et al. Qualitative and quantitative analysis of iridoid glycosides in the flower buds of Lonicera species by capillary high performance liquid chromatography coupled with mass spectrometric detector. *Anal. Chim. Acta* **564**, 211-218 (2006).
14. Zou, C., Wang, Y. & Shen, Z. 2-NBDG as a fluorescent indicator for direct glucose uptake measurement. *J. Biochem. Biophys. Methods* **64**, 207-215 (2005).
15. Halasz, P., Holloway, G., Turner, S. J. & Coulson, B. S. Rotavirus replication in intestinal cells differentially regulates integrin expression by a phosphatidylinositol 3-kinase-dependent pathway, resulting in increased cell adhesion and virus yield. *J. Virol.* **82**, 148-160 (2008).
16. Soliman, M. et al. Opposite effects of apoptotic and necroptotic cellular pathways on rotavirus replication. *J. Virol.* **96**, e0122221 (2022).
17. Trompette, A. et al. Gut-derived short-chain fatty acids modulate skin barrier integrity by promoting keratinocyte metabolism and differentiation. *Mucosal Immunol.* Epub ahead of print (2022).
18. Tu L. N. et al. Translocator protein (TSPO) affects mitochondrial fatty acid oxidation in steroidogenic cells. *Endocrinology* **157**, 1110-1121 (2016).
19. Golebski, K. et al., Induction of IL-10-producing type 2 innate lymphoid cells by allergen immunotherapy is associated with clinical response. *Immunity.* **54**, 291-307 (2021).
20. Mayer, N. et al. Development of small-molecule inhibitors targeting adipose triglyceride lipase. *Nat. Chem. Biol.* **9**, 785-787 (2013).
21. Brown, E. G. Increased virulence of a mouse-adapted variant of influenza A/FM/1/47 virus is controlled by mutations in genome segments 4, 5, 7, and 8. *J. Virol.* **64**, 4523-4533 (1990).
22. Ilyushina, N. A. et al. Adaptation of pandemic H1N1 influenza virus in mice. *J. Virol.* **84**, 8607-8616 (2010).
23. Mueller, S. et al. Live attenuated influenza virus vaccines by computer-aided rational design. *Nat. Biotechnol.* **28**, 723-726 (2010).
24. Ramakrishnan, M. A. Determination of 50% endpoint titer using a simple formula. *World J. Virol.* **5**, 85-86 (2016).
25. Tate, M. D., Brooks, A. G. & Reading, P. C. The role of neutrophils in the upper and lower respiratory tract during influenza virus infection of mice. *Respir. Res.* **9**, 1-13 (2008).
26. Reed, L. J. & Muench, H. A simple method of estimating fifty percent endpoints. *Am. J. Epidemiol.* **27**, 493-497 (1938).
27. Kim, H. J. et al. Detection and genotyping of Korean porcine rotaviruses. *Vet. Microbiol.* **144**, 274-286 (2010).
28. Monson, E. et al. Intracellular lipid droplet accumulation occurs early following viral infection and is required for an efficient interferon response. *Nat. Commun.* **12**, 1-17 (2021).
29. Parra-Vargas, M. et al. Delphinidin ameliorates hepatic triglyceride accumulation in human HepG2 cells, but not in diet-induced obese mice. *Nutrients* **10**, 1060 (2018).
30. Lai, S.-C., Phelps, C. A., Short, A. M., Dutta, S. M. & Mu, D. Thyroid transcription factor 1 enhances cellular statin sensitivity via perturbing cholesterol metabolism. *Oncogene* **37**, 3290-3300 (2018).
31. Lee, K. H. et al. Inhibitory effect of emodin on fatty acid synthase, colon cancer proliferation and apoptosis. *Mol. Med. Report.* **15**, 2163-2173 (2017).
32. Siderius, M., Van Wuytswinkel, O., Reijenga, K. A., Kelders, M. & Mager, W. H. The control of intracellular glycerol in Saccharomyces cerevisiae influences osmotic stress response and resistance to increased temperature. *Mol. Microbiol.* **36**, 1381-1390 (2000).
33. Morita, M. et al. The lipid mediator protectin D1 inhibits influenza virus replication and improves severe influenza. *Cell* **153**, 112-125 (2013).
34. Schmittgen, T. D. & Livak, K. J. Analyzing real-time PCR data by the comparative C(T) method. *Nat. Protoc.* **3**, 1101-1118 (2008).
35. Sia, S. F. et al. Pathogenesis and transmission of SARS-CoV-2 in golden hamsters. *Nature* **583**, 834-838 (2020).
36. Wang, J. et al. Influenza virus exploits an interferon-independent lncRNA to preserve viral RNA synthesis through stabilizing viral RNA polymerase PB1. *Cell Rep.* **27**, 3295-3304. e3294 (2019).
37. Schwarz, B. A. et al. Detection and quantitation of group A rotaviruses by competitive and real-time reverse transcription-polymerase chain reaction. *J. Virol. Methods* **105**, 277-285 (2002).
38. Kim, Y. et al. Trypsin-independent porcine epidemic diarrhea virus US strain with altered virus entry mechanism. *BMC Vet. Res.* **13**, 1-13 (2017).
39. Xiao, S. et al. Inhibition of highly pathogenic PRRSV replication in MARC-145 cells by artificial microRNAs. *Virol J.* **8**, 1-11 (2011).
40. Amer, H. M. & Almajhdi, F. N. Development of a SYBR Green I based real-time RT-PCR assay for detection and quantification of bovine coronavirus*. Mol. Cell. Probes* **25**, 101-107 (2011).
41. Paz, J. C. et al. Combinatorial regulation of a signal-dependent activator by phosphorylation and acetylation. *PNAS* **111**, 17116-17121 (2014).
42. Barr, F. G. et al. Examination of gene fusion status in archival samples of alveolar rhabdomyosarcoma entered on the Intergroup Rhabdomyosarcoma Study-III trial: a report from the Children's Oncology Group. *J. Mol. Diagn.* **8**, 202-208 (2006).
43. Kuefner, M. S. et al. Secretory phospholipase A2 group IIA modulates insulin sensitivity and metabolism. *J. Lipid Res.* **58**, 1822-1833 (2017).
44. Deng, X. et al. Development and utilization of an infectious clone for porcine deltacoronavirus strain USA/IL/2014/026. *Virology* **553**, 35-45 (2021).
45. Gorman, M. et al., An immunocompetent mouse model of Zika virus infection. *Cell Host Microbe.* **23**, 672-685 (2018).
46. Chiu, C. et al. Nonalcoholic fatty liver disease is exacerbated in high-fat diet-fed gnotobiotic mice by colonization with the gut microbiota from patients with nonalcoholic steatohepatitis. *Nutrients* **9**, 1220 (2017).
47. Yamakawa, I. et al. Inactivation of TNF-α ameliorates diabetic neuropathy in mice. *Am. J. Physiol. Endocrinol. Metab.* **301**, E844-52 (2011).
48. Qiu, T. et al. Obesity-induced elevated palmitic acid promotes inflammation and glucose metabolism disorders through GPRs/NF-κB/KLF7 pathway. *Nutr. Diabetes*. **12**, 23 (2022).
49. Zhang, Y. et al. A highly efficacious live attenuated mumps virus-based SARS-CoV-2 vaccine candidate expressing a six-proline stabilized prefusion spike. *Proc. Natl. Acad. Sci. U. S. A.* **119**, e2201616119 (2022).
50. Francis, M. et al. SARS-CoV-2 infection in the Syrian hamster model causes inflammation as well as type I interferon dysregulation in both respiratory and non-respiratory tissues including the heart and kidney. *PLoS Pathog.* **17**, e1009705 (2021).
51. Yang, S. et al. Characterization of virus replication, pathogenesis, and cytokine responses in Syrian hamsters inoculated with SARS-CoV-2. *J. Inflamm. Res.* **14**, 3781-3795 (2021).
52. Hamilton, J. et al. Cutting Edge: Intracellular IFN-β and distinct type I IFN expression patterns in circulating systemic lupus erythematosus B cells. *J. Immunol.* **201**, 2203-2208 (2022).
53. Miao, L. et al. Targeting the STING pathway in tumor-associated macrophages regulates innate immune sensing of gastric cancer cells. *Theranostics* **10**, 498-515 (2020).

**
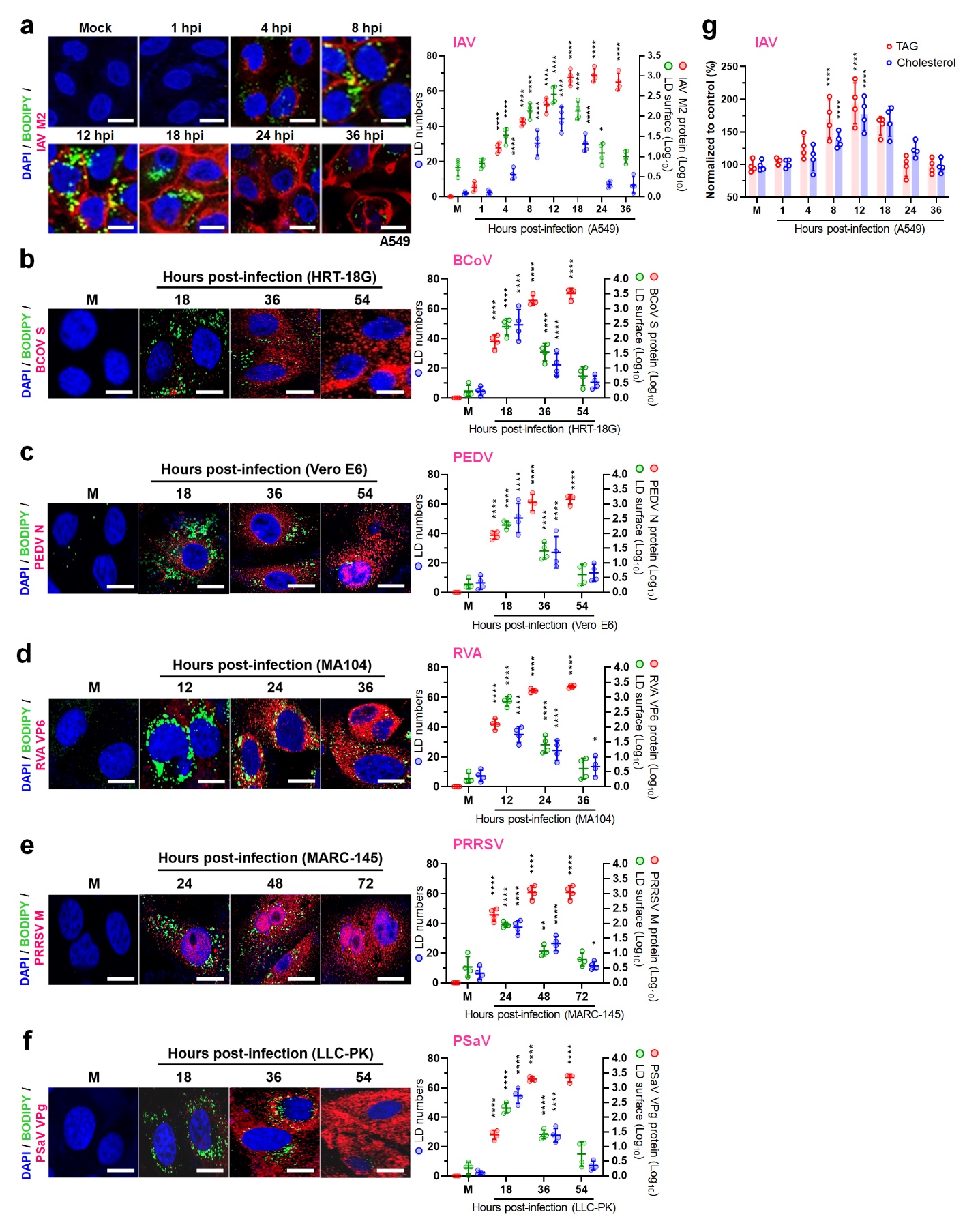
**

**Figure S1. *In vitro* dynamics of formation and breakdown of lipid droplets (LDs) during RNA viral replication.** (a-f) Representative images (left) and quantification (right) of sequential changes of BODIPY-stained intracellular LDs (green) and viral antigen (red) in A549 cells infected with influenza A virus PR8 strain at an MOI of 1 FFU (a), HRT-18G cells infected with bovine coronavirus (BCoV) KWD strain at an MOI of 0.1 FFU (b), Vero E6 cells infected with porcine epidemic diarrhea coronavirus (PEDV) QIAP1401 strain at an MOI of 0.1 FFU (c), MA104 cells infected with bovine species A rotavirus (RVA) NCDV strain at an MOI of 0.1 FFU (d), MARC-145 cells infected with porcine reproductive and respiratory syndrome virus (PRRSV) LMY strain at an MOI of 0.1 FFU (e), and LLC-PK cells infected with porcine sapovirus (PSaV) Cowden strain at an MOI of 0.1 FFU (f). (g) Quantification of sequential changes of intracellular triacylglycerol (TAG) and cholesterol in the IAV-infected A549 cells as noted above. All data in the graphs are presented as arithmetic means ± S.D. from four independent experiments. One-way analysis of variance was carried out with Tukey’s correction for multiple comparisons. **P* < 0.05, ***P* < 0.01, ****P* < 0.001, *****P* < 0.0001. Scale bars = 25 µm.

**
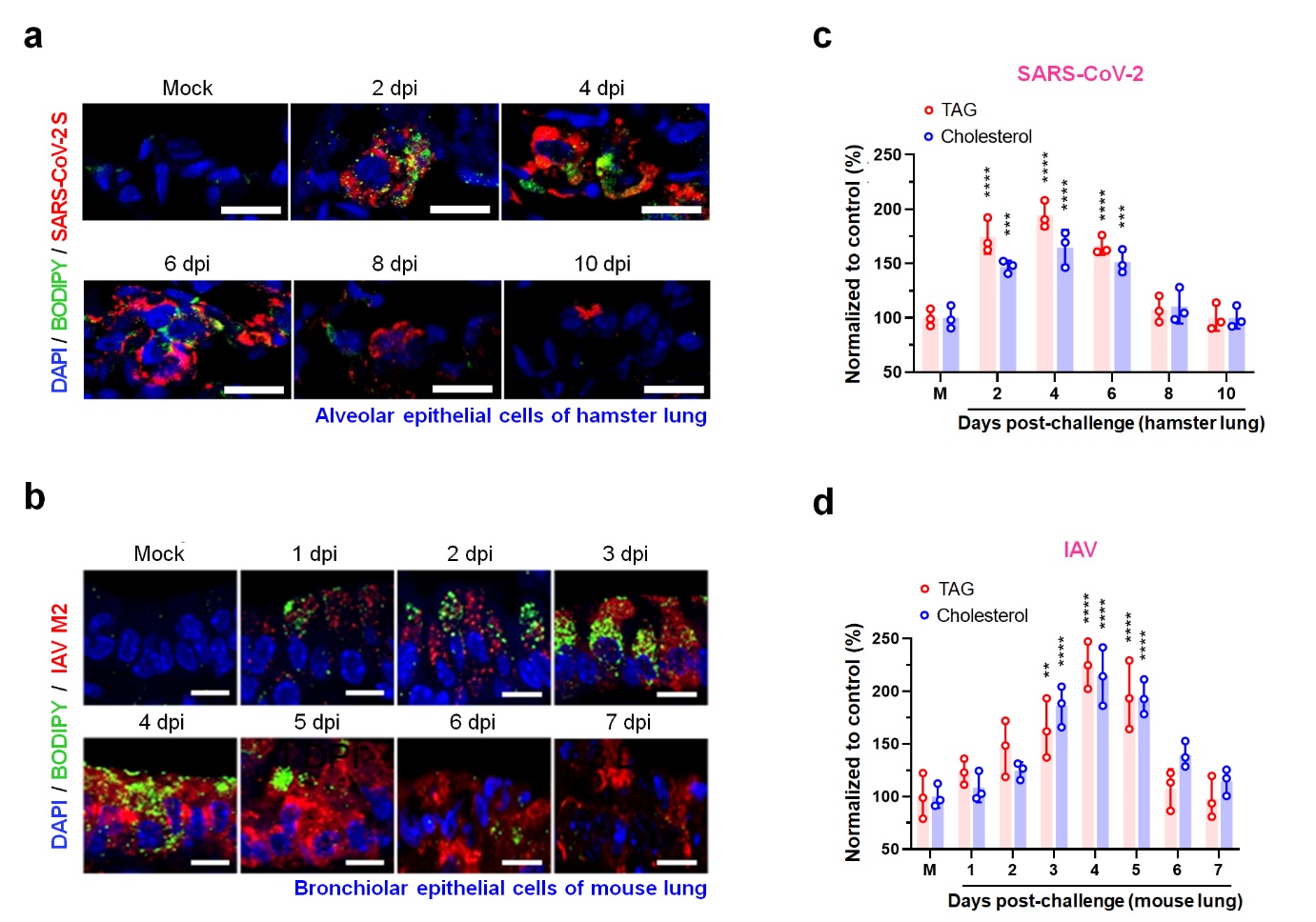
Figure S2. *In vivo* dynamics of formation and breakdown of LDs in SARS-CoV-2-challenged hamsters and IAV-challenged mice.** (a) Representative images of sequential changes of BODIPY-stained intracellular LDs (green) and SARS-CoV-2 S antigen (red) in the alveolar epithelial cells of lung tissues sampled sequentially from hamsters challenged with 10^5^ TCID_50_ of SARS-CoV-2 KCDC03 strain. (b) Representative images of sequential changes of BODIPY-stained intracellular LDs (green) and IAV M2 antigen (red) in the bronchiolar epithelial cells of lung tissues sampled sequentially from mice challenged with 10^3^ PFU of mouse-adapted IAV PR8 strain. (c, d) Quantification of sequential changes of triacylglycerol (TAG) and cholesterol in the lung tissues sampled from SARS-CoV-2-challenged hamsters (c) and IAV-challenged mice (d). All data in the graphs are presented as arithmetic means ± S.D. from three animals each. One-way analysis of variance was carried out with Tukey’s correction for multiple comparisons. **P* < 0.05, ***P* < 0.01, ****P* < 0.001, *****P* < 0.0001. Scale bars = 30 µm.

**
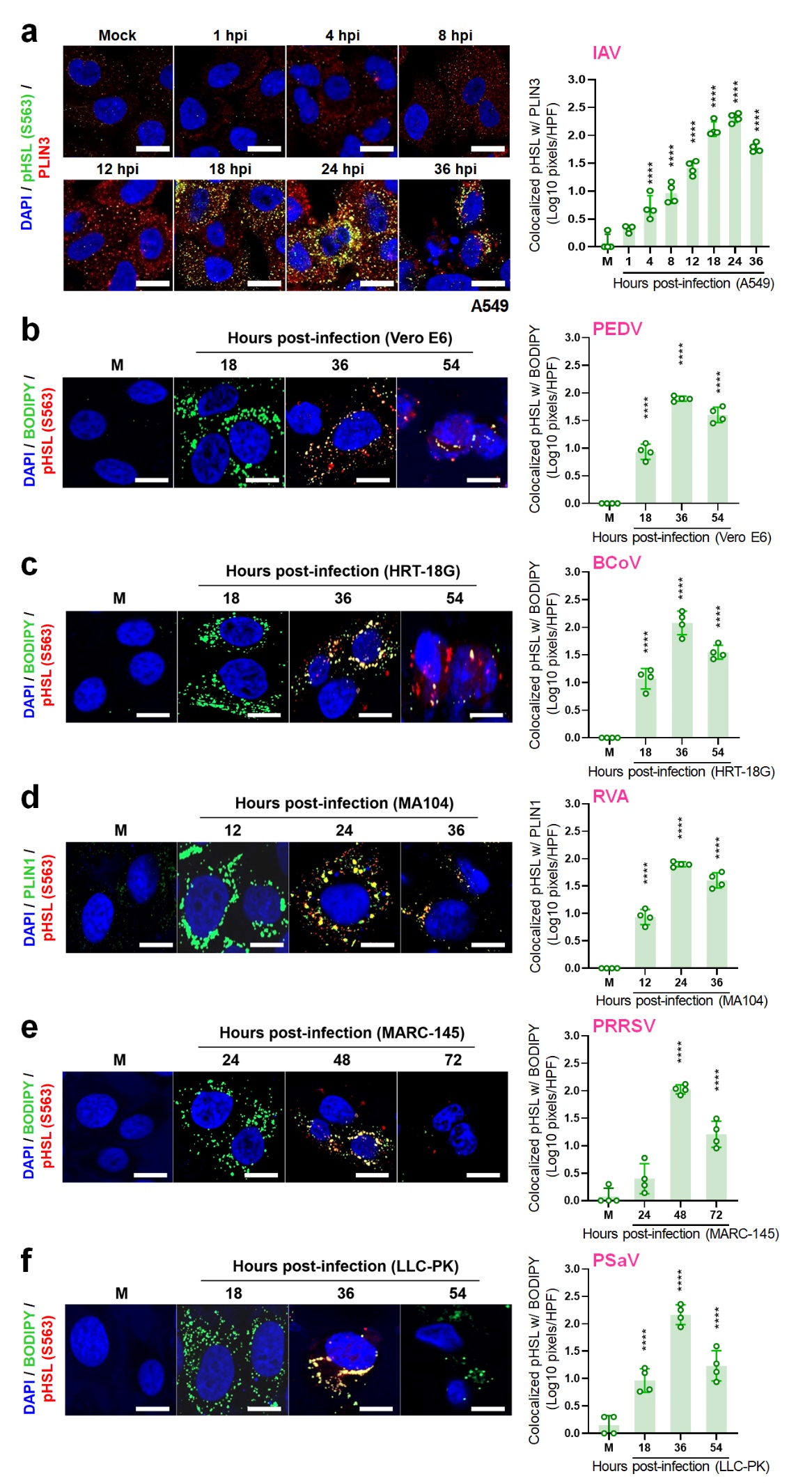
**

**Figure S3. *In vitro* activation of LD-associated hormone-sensitive lipase (HSL) during viral RNA replication.** (a-f) Representative images (left) and quantification (right) of lipolytic phosphorylated HSL (pHSL, green or red) colocalized with an LD coating protein PLIN1 (green) or PLIN3 (red) or BODIPY-stained intracellular LDs (green) in A549 cells infected with influenza A virus PR8 strain at an MOI of 1 FFU (a), HRT-18G cells infected with bovine coronavirus (BCoV) KWD strain at an MOI of 0.1 FFU (b), Vero E6 cells infected with porcine epidemic diarrhea coronavirus (PEDV) QIAP1401 strain at an MOI of 0.1 FFU (c), MA104 cells infected with bovine species A rotavirus (RVA) NCDV strain at an MOI of 0.1 FFU (d), MARC-145 cells infected with porcine reproductive and respiratory syndrome virus (PRRSV) LMY strain at an MOI of 0.1 FFU (e), and LLC-PK cells infected with porcine sapovirus (PSaV) Cowden strain at an MOI of 0.1 FFU (f). All data in the graphs are presented as arithmetic means ± S.D. from four independent experiments. One-way analysis of variance was carried out with Tukey’s correction for multiple comparisons. **P* < 0.05, ***P* < 0.01, ****P* < 0.001, *****P* < 0.0001. Scale bars = 25 µm.

**
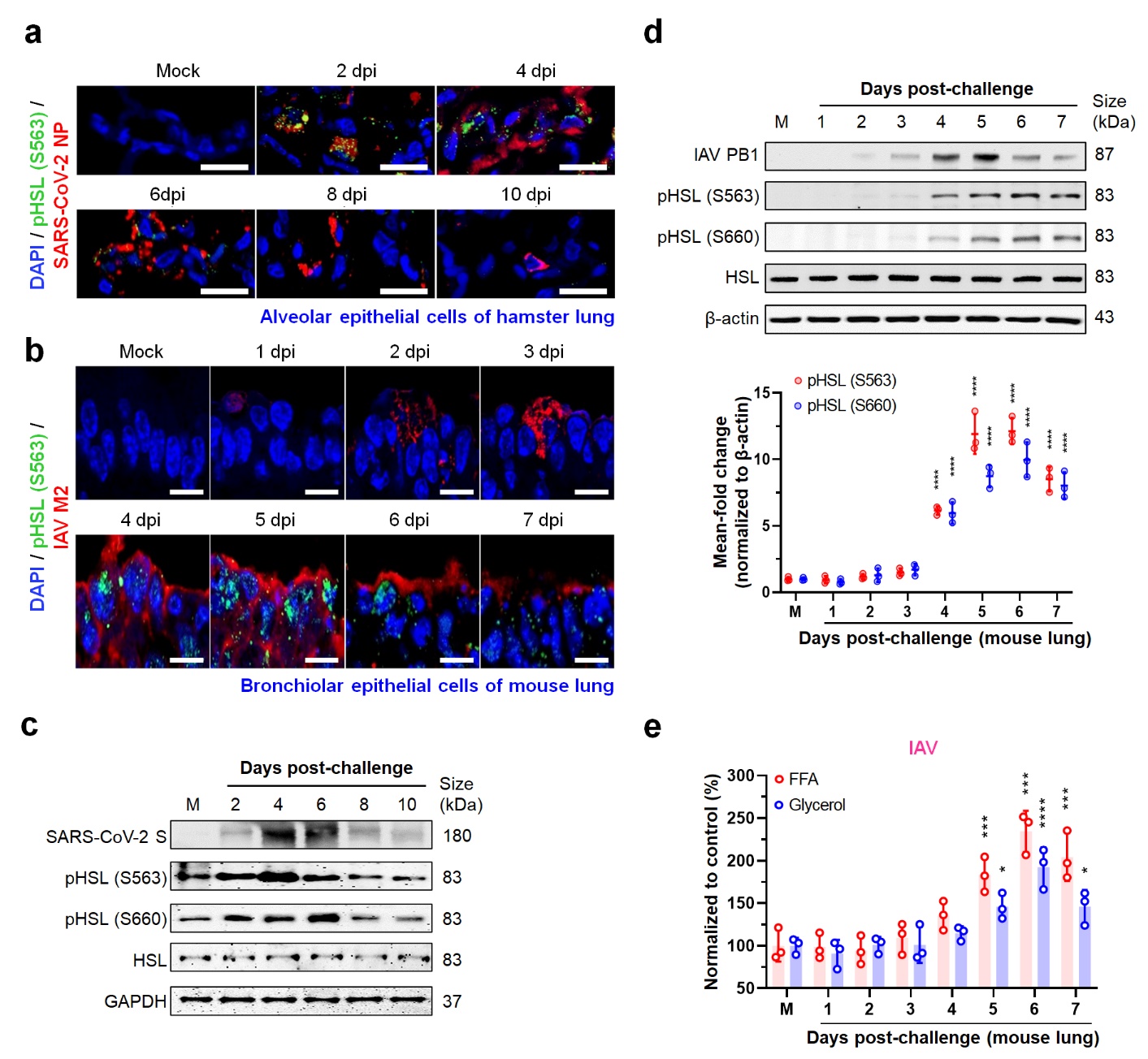
Figure S4. *In vivo* activation of LD-associated HSL in SARS-CoV-2-challenged hamsters and IAV-challenged mice.** (a) Representative images of pHSL (S563, green) and SARS-CoV-2 NP antigen (red) levels in the alveolar epithelial cells of lung tissues obtained sequentially from Syrian hamsters challenged with 10^5^ TCID_50_ of SARS-CoV-2 KCDC03 strain. (b) Representative images of pHSL (S563, green) and IAV M2 antigen (red) levels in the bronchial epithelial cells of lung tissues obtained sequentially from mice challenged with 10^3^ PFU of mouse-adapted IAV PR8 strain. (c) Representative western blot. Dynamic of lipolytic phosphorylated HSLs (pHSLs, S563, and S660) in lung tissues sampled sequentially from Syrian hamsters challenged with 10^5^ TCID_50_ of SARS-CoV-2 KCDC03 strain. (d) Representative western blot and quantification. Dynamic of lipolytic phosphorylated HSLs (pHSLs, S563, and S660) in lung tissues sampled sequentially from mice challenged with 10^3^ PFU of mouse-adapted IAV PR8 strain. (e) Dynamic of intracellular free fatty acids and glycerol in lung tissues obtained sequentially from mice challenged with 10^3^ PFU of IAV PR8 strain. All data in the graphs are derived from three animals. One-way analysis of variance was carried out with Tukey’s correction for multiple comparisons. **P* < 0.05, ***P* < 0.01, ****P* < 0.001, *****P* < 0.0001. Scale bars = 50 µm.

**
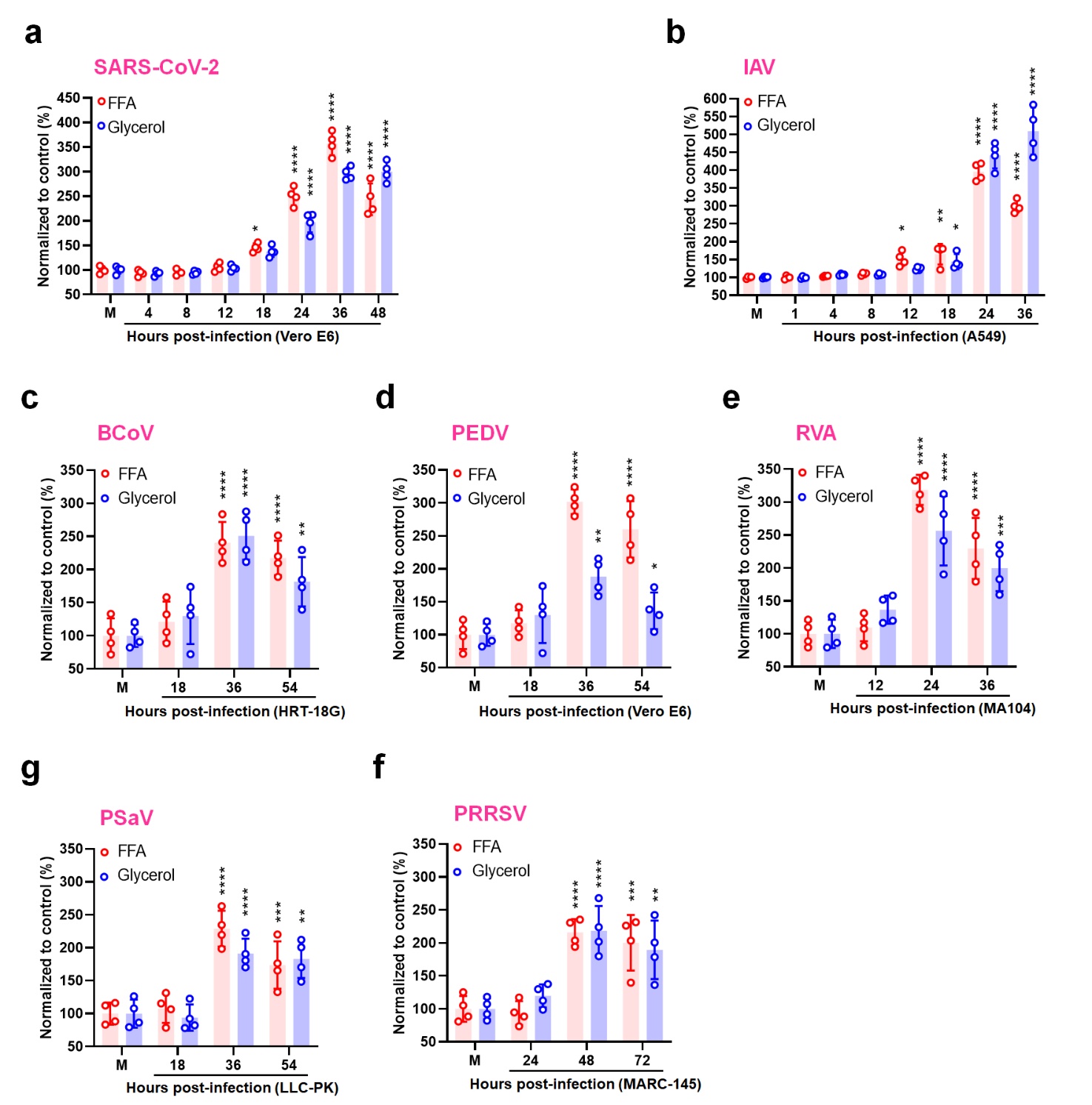
**

**Figure S5. The *in vitro* dynamics of intracellular FFAs and glycerol during RNA viral replication.** (a-g) Quantification of intracellular FFA and glycerol levels in Vero E6 cells infected with SARS-CoV-2 KCDC03 strain at an MOI of 0.1 (a), A549 cells infected with influenza A virus (IAV) PR8 strain at an MOI of 1 FFU (b), HRT-18G cells infected with bovine coronavirus (BCoV) KWD strain at an MOI of 0.1 FFU (c), Vero E6 cells infected with porcine epidemic diarrhea coronavirus (PEDV) QIAP1401 strain at an MOI of 0.1 FFU (d), MA104 cells infected with bovine species A rotavirus (RVA) NCDV strain at an MOI of 0.1 FFU (e), MARC-145 cells infected with porcine reproductive and respiratory syndrome virus (PRRSV) LMY strain at an MOI of 0.1 FFU (f), and LLC-PK cells infected with porcine sapovirus (PSaV) Cowden strain at an MOI of 0.1 FFU (g). All data in the graphs are presented as arithmetic means ± S.D. from four independent experiments. One-way analysis of variance was carried out with Tukey’s correction for multiple comparisons. **P* < 0.05, ***P* < 0.01, ****P* < 0.001, *****P* < 0.0001.


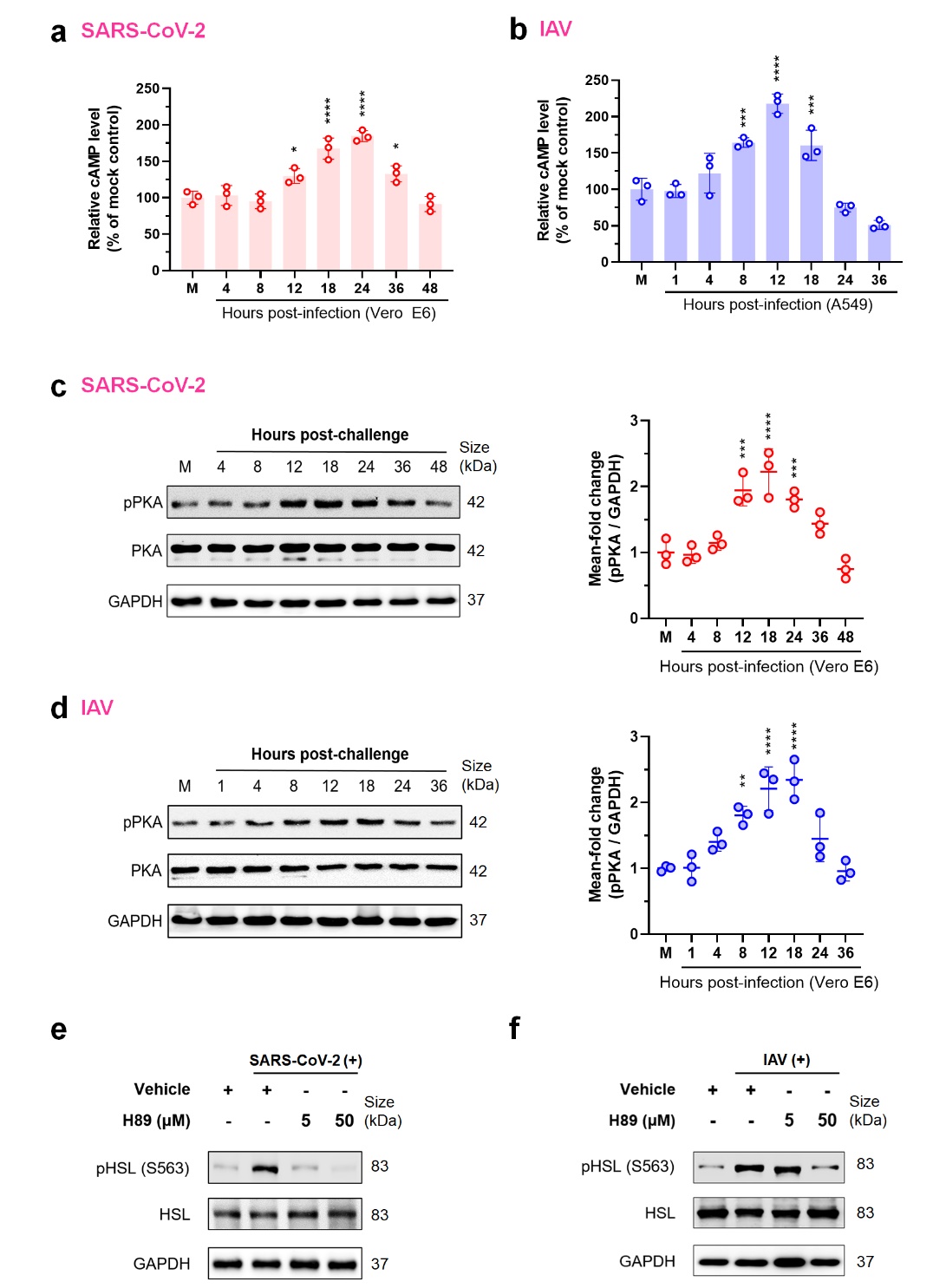


**Figure S6. cAMP-PKA pathway-mediated activation of HSL.** (a, b) Sequential changes of intracellular cAMP level in Vero E6 cells infected with SARS-CoV-2 at an MOI of 0.1 FFU (a) and A549 cells infected with IAV at an MOI of 1 FFU (b). (c, d) Representative western blot images and graphical representations. Dynamic of phosphorylated PKA-C subunit α (PKA-C-α) in the cells infected with either SARS-CoV-2 (c) or IAV (d) in the above condition (a, b). (e, f) Representative western blot images of pHSL levels in the SARS-CoV-2-infected Vero E6 cells (MOI = 0.1 FFU) at 18 hpi and IAV-infected A549 cells (MOI = 1 FFU) at 12 hpi in the absence or presence of PKA inhibitor H89 (5 μM or 50 μM). All data in the graphs are presented as arithmetic means ± S.D. from three independent experiments. For statistical analysis, a one-way analysis of variance was carried out with Tukey’s correction for multiple comparisons. **P* < 0.05, ***P* < 0.01, ****P* < 0.001, *****P* < 0.0001.

**
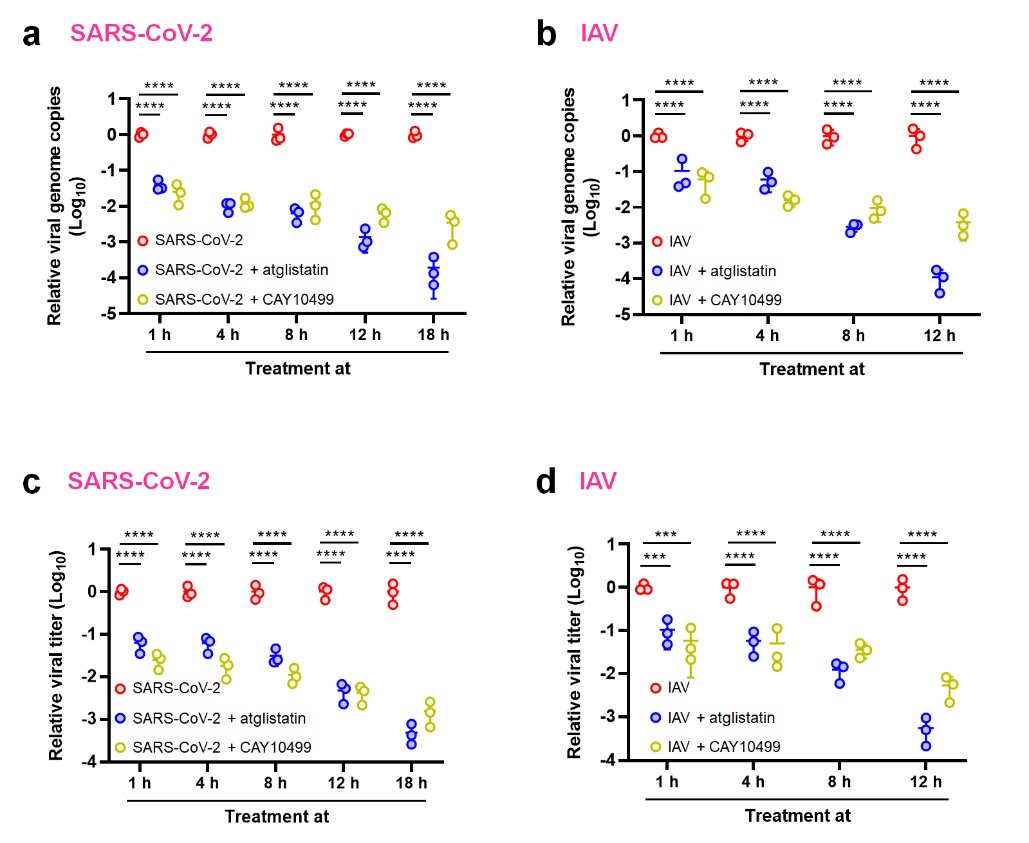
**

**Figure S7. *In vitro* antiviral activities of lipase inhibitors at different time points after infection with SARS-CoV-2 or IAV.** SARS-CoV-2-infected Vero E6 cells (MOI of 0.01 FFU) and IAV-infected A549 cells (MOI of 0.01 FFU) were treated with atglistatin or CAY10499 at 20 μM concentration after virus infection at different time points as indicated. (a-d) Different antiviral effects of atglistatin and CAY10499 on reduction in viral genome copy numbers (a, b) and viral titers (c, d) in the SARS-CoV-2-infected Vero E6 cells and IAV-infected A549 cells, respectively. All data in the graphs are presented as arithmetic means ± S.D. from three independent experiments. For statistical analysis, a one-way analysis of variance was carried out with Tukey’s correction for multiple comparisons. **P* < 0.05, ***P* < 0.01, ****P* < 0.001, *****P* < 0.0001.

**
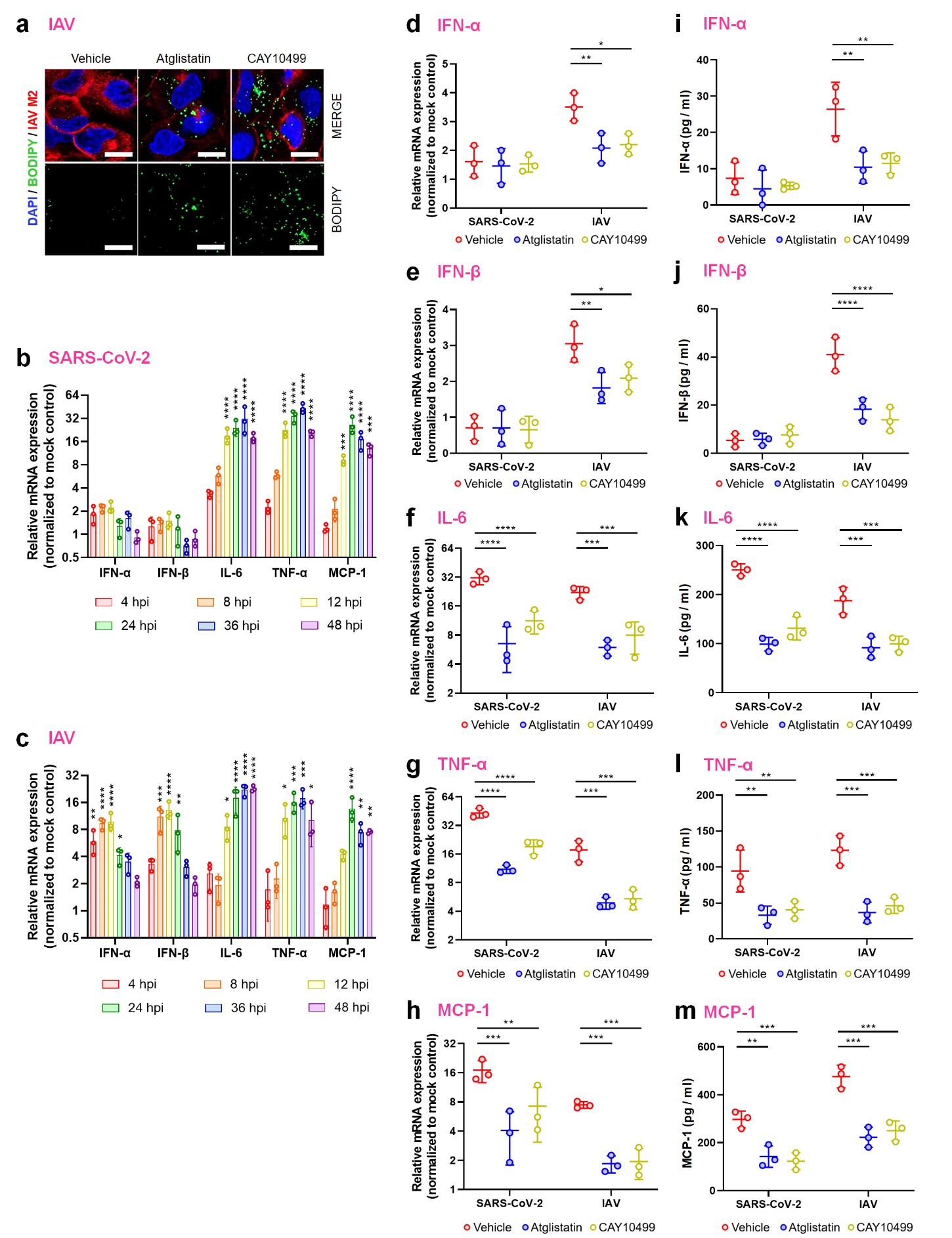
**

**Figure S8. Inhibitory effects of lipase inhibitors on LD lipolysis and proinflammatory cytokines in SARS-CoV-2- and IAV-infected cells.** (a) Retention of LDs (green) with corresponding inhibition of IAV replication (red) by the treatment of virus-infected cells (MOI = 1 FFU) with atglistatin or CAY10499. Scale bar = 50 µm. (b, c) Sequential changes of proinflammatory cytokine gene expressions (IFN-α, IFN-β, TNF-α, IL-6, and MCP-1) in SARS-CoV-2-infected Vero E6 cells (MOI = 0.1 FFU) and IAV-infected A549 cells (MOI = 1 FFU). The cell lysate from each group was used for RT-qPCR. The qPCR results were normalized by the housekeeping gene GAPDH. (d-h) Inhibitory effects of atglistatin and CAY10499 at 20 μM concentration on the expression level of proinflammatory cytokine genes (IFN-α, IFN-β, TNF-α, IL-6, and MCP-1) in SARS-CoV-2-infected Vero E6 cells (MOI = 0.1 FFU) and IAV-infected A549 cells (MOI = 1 FFU) compared to mock-treated virus-infected control. SARS-CoV-2- and IAV-infected cells were treated with both chemicals at 18 hpi and at 12 hpi, respectively, and incubated further for 18 h and 12 h (Supplementary Table 4). The cell lysate from each group was used for RT-qPCR. The RT-qPCR results were normalized by the housekeeping gene GAPDH, and relative changes of chemical-treated groups for each cytokine gene were compared to the vehicle-treated controls. (i-m) The levels of proinflammatory cytokines (IFN-α, IFN-β, TNF-α, IL-6, and MCP-1) in the supernatant harvested as described in the above condition (d-h) were determined by ELISA. Relative reduction of chemical-treated groups for each cytokine was compared to the vehicle-treated controls. All data in the graphs are presented as arithmetic means ± S.D. from three independent experiments. For statistical analysis, a one-way analysis of variance was carried out with Tukey’s correction for multiple comparisons. **P* < 0.05, ***P* < 0.01, ****P* < 0.001, *****P* < 0.0001.

**
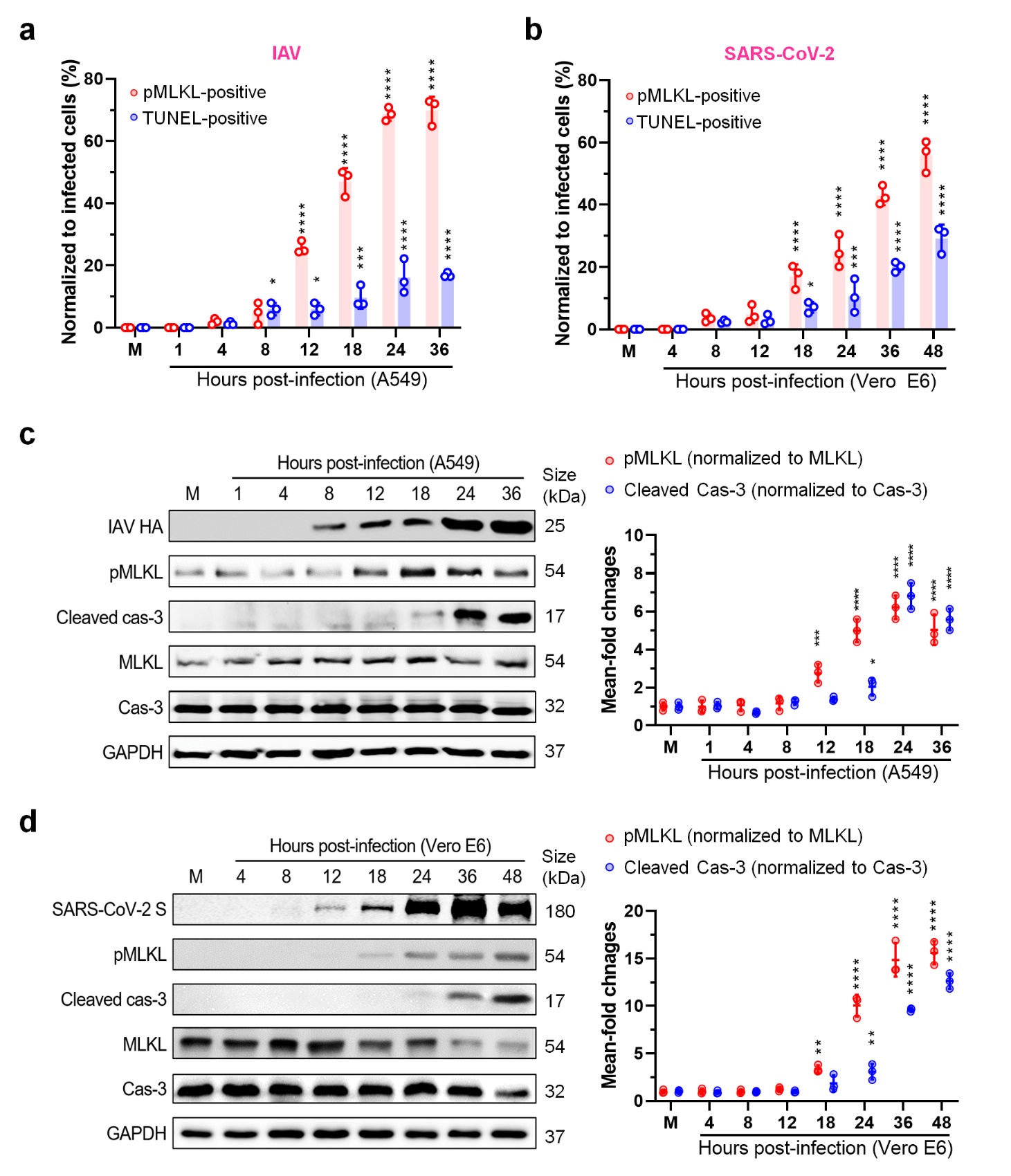
**

**Figure S9. Sequential changes of necroptosis marker- and apoptosis marker-positive cells in either IAV or SARS-CoV-2-infected cells.** (a, b) Sequential changes of virus-induced death-signal positive cells. Cells infected with either IAV PR8 strain (MOI = 1) (a) or SARS-CoV-2 KCDC03 strain (MOI = 0.1) (b) were dual stained to detect necroptosis marker pMLKL (using the antibody against pMLKL)- and apoptosis marker TUNEL (using TUNEL assay)-positive cells which were all positive for either IAV M2 protein or SARS-CoV-2 S protein. The quantification of necroptosis marker-positive (dual positive for viral antigen and pMLKL), apoptosis marker-positive (dual positive for viral antigen and TUNEL), and viable cells were assessed by flow cytometry analysis and expressed as a percentage in the total cells. (c, d) Representative western blot images and graphical representation. The cell lysates in the above condition were subjected to western blot analysis to check the expression levels of the indicated proteins. GAPDH was used as a loading control. The band intensities of pMLKL and cleaved caspase-3 relative to the total proteins were determined by densitometric analysis and shown as graphs. All data in the graphs are presented as arithmetic means ± S.D. from four independent experiments. For statistical analysis, a one-way analysis of variance was carried out with Tukey’s correction for multiple comparisons. **P* < 0.05, ***P* < 0.01, ****P* < 0.001, *****P* < 0.0001.

**
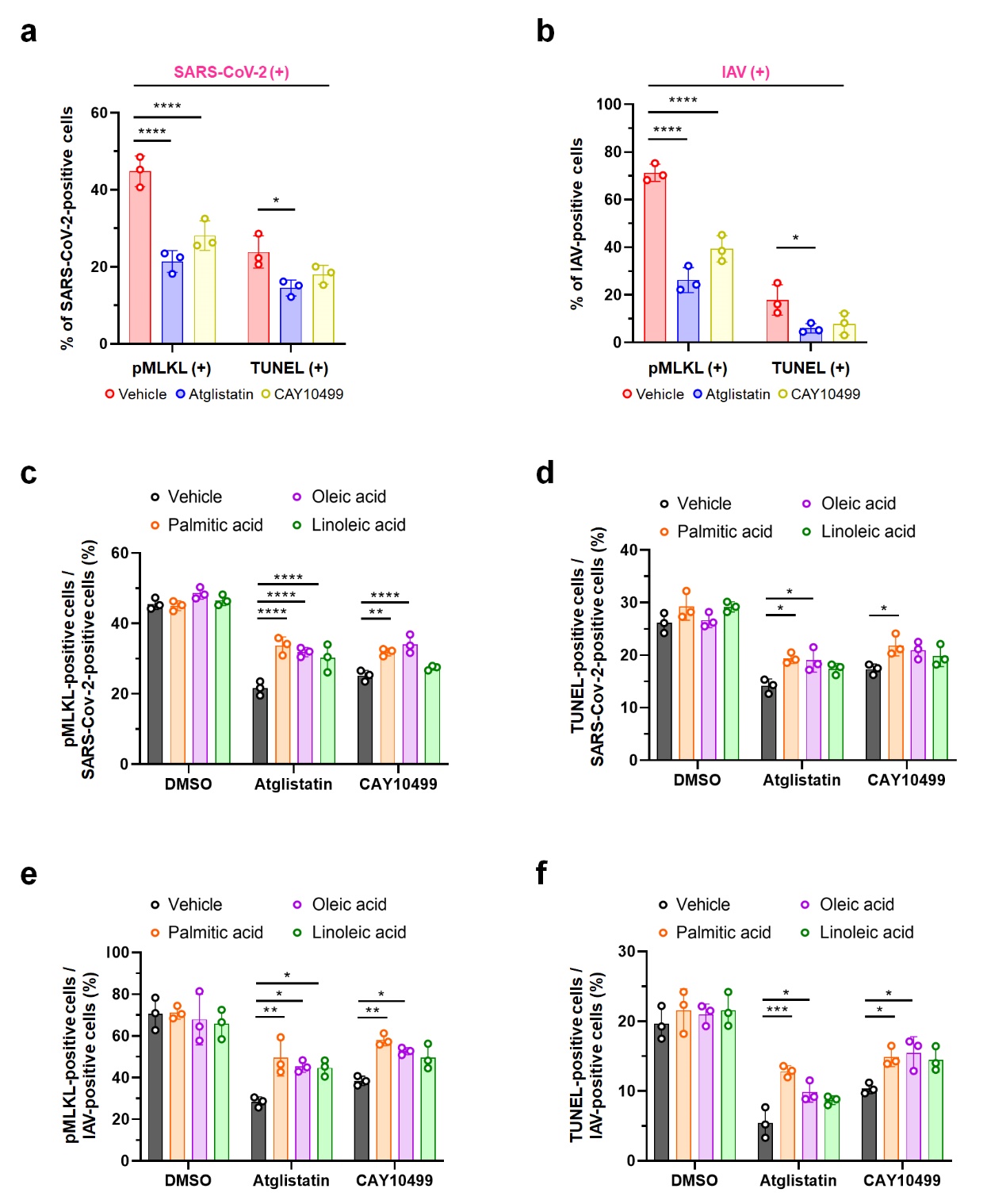
**

**Figure S10. Increase in the number of cell death marker-positive virus-infected cells through supplementation of exogenous FFAs in the FFA deprived condition.** (a, b) Graphical representation of the inhibitory effect of atglistatin and CAY10499 at 20 μM concentration on the rates of necroptosis marker pMLKL- and apoptosis marker TUNEL-positive cells that were infected with SARS-CoV-2 (MOI = 0.1) or IAV (MOI = 1) and incubated for 24 hpi and 36 hpi, respectively. (c-f) Graphical representation of supplementary effects of FFAs on cell death. The SARS-CoV-2-infected Vero E6 cells (MOI = 0.1 FFU) and IAV-infected A549 cells (MOI = 1 FFU) were treated with vehicle, atglistatin, or CAY10499 (20 μM) at 12 hpi and 18 hpi, respectively, and then mock-supplemented or supplemented with palmitic acid, oleic acid, or linoleic acid at 100 μM concentration. The cells were further incubated for 18 h for SARS-CoV-2 or 12 h for IAV. The quantification of necroptosis marker-positive (dual positive for viral antigen and pMLKL), apoptosis marker-positive (dual positive for viral antigen and TUNEL), and viable cells were assessed by flow cytometry analysis and expressed as a percentage in the total cells. All data in the graphs are presented as arithmetic means ± S.D. from three independent experiments. For statistical analysis, a one-way analysis of variance was carried out with Tukey’s correction for multiple comparisons. **P* < 0.05, ***P* < 0.01, ****P* < 0.001, *****P* < 0.0001.

**
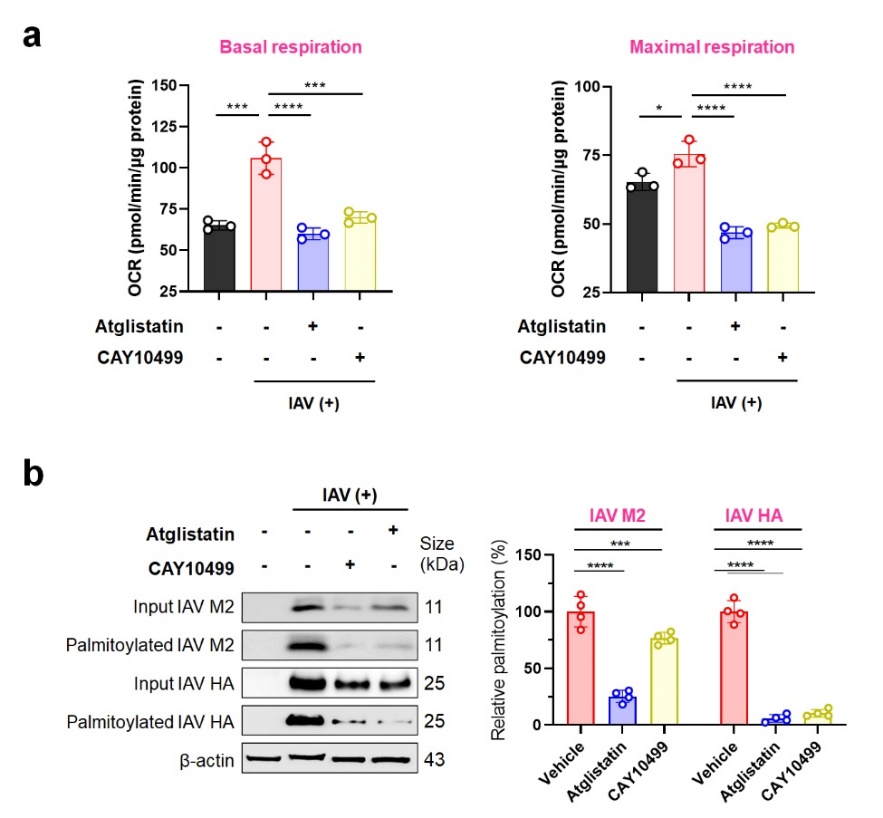
**

**Figure S11. Effect of lipase inhibitors on oxidation consumption rate (OCR) and virus protein palmitoylation in either SARS-CoV-2- or IAV-infected cells.** (a) Graphical representation of OCRs in IAV-infected A549 cells (MOI = 1 FFU) in the absence or presence of atglistatin and CAY10499 (20 μM) at 24 h post-infection. Increased endogenous basal and maximal OCRs during IAV infection was significantly suppressed by treatment of virus-infected cells with lipase inhibitors. (b) Representative western blot (left) and quantification (right) of inhibitory effects of atglistatin and CAY10499 on palmitoylation of IAV HA and M2 proteins determined in virus-infected A549 cells (MOI = 1 FFU) at 24 h post-infection. All data in the graphs are presented as arithmetic means ± S.D. from three independent experiments. For statistical analysis, a one-way analysis of variance was carried out with Tukey’s correction for multiple comparisons. **P* < 0.05, ***P* < 0.01, ****P* < 0.001, *****P* < 0.0001.

**
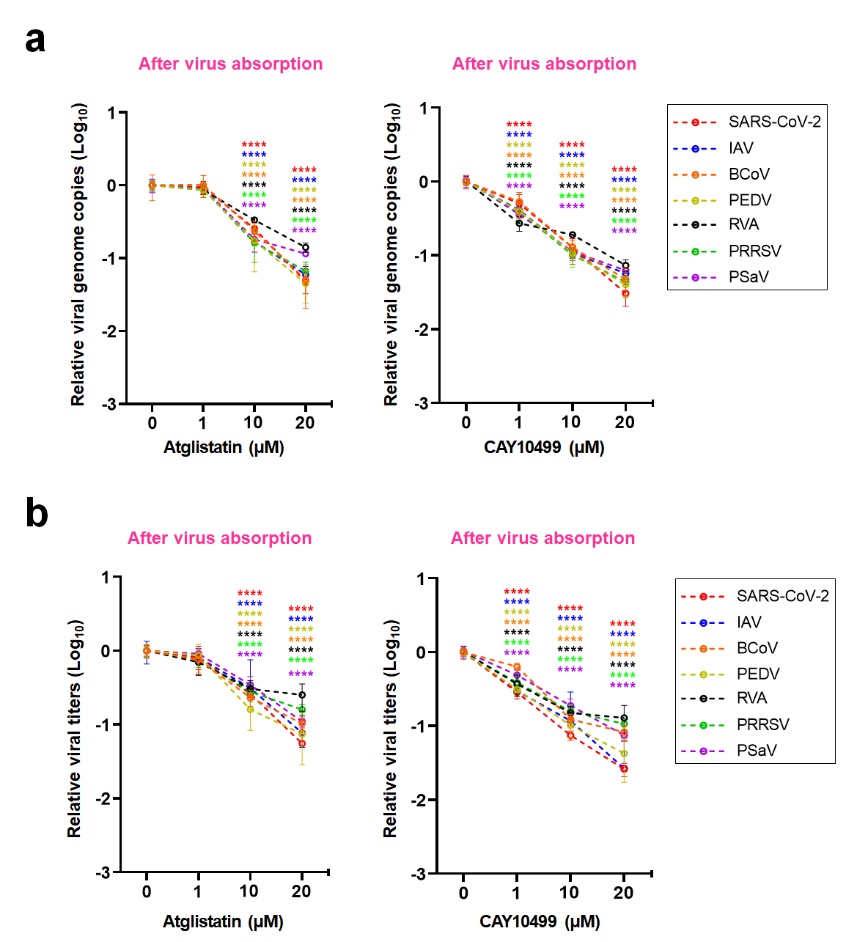
**

**Figure S12. *In vitro* antiviral effect of lipase inhibitors.** (a, b) Effect of atglistatin and CAY10499 on reduction in viral genome copy numbers and viral infectivity titers for seven different RNA viruses in infected cells when treated immediately after virus absorption. Treatment times are shown in Supplementary Table 4. All data in the graphs are presented as arithmetic means ± S.D. from four independent experiments. One-way analysis of variance with Tukey’s correction for multiple comparisons. **P* < 0.05, ***P* < 0.01, ****P* < 0.001, *****P* < 0.0001.

**
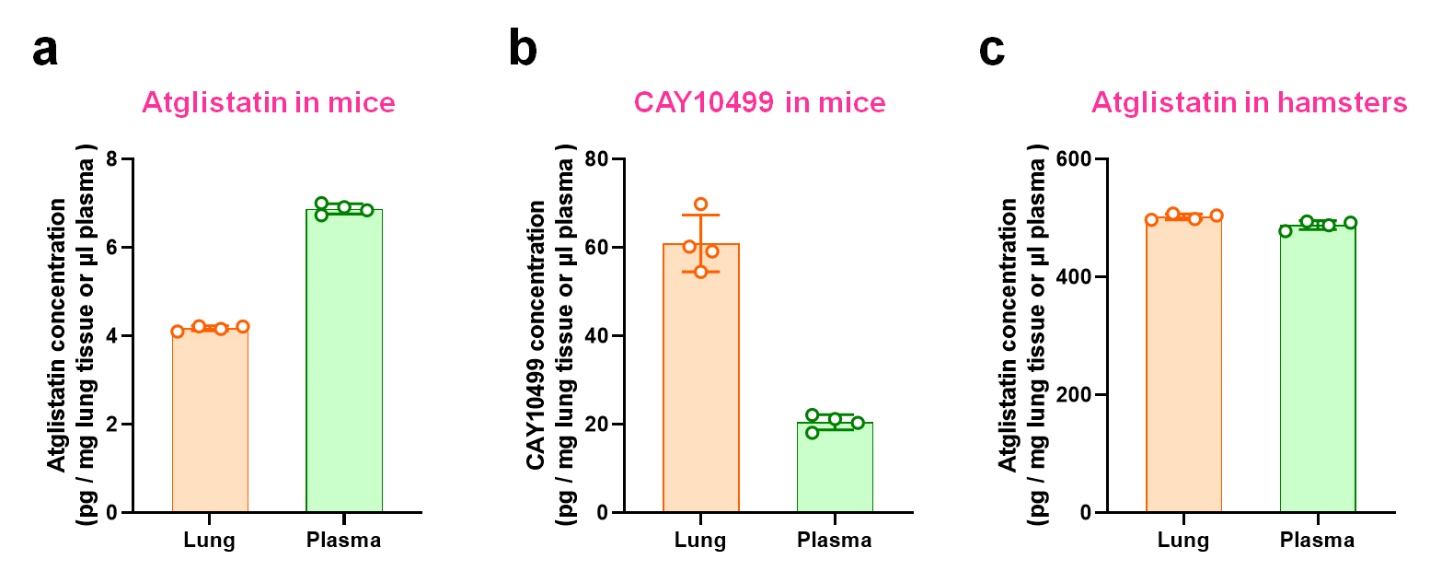
**

**Figure S13. Lung and plasma distribution of atglistatin and CAY10499 in mice and hamsters.** (a, b) Graphical representation of the amount of atglistatin and CAY10499 in the lung and plasma of mice. For the detection of atglistatin (a) and CAY10499 (b) in wild-type C57Bl/6J mice, 2 groups of 4 anesthetized mice were treated intraperitoneally with either atglistatin or CAY10499 in a 50 μL vehicle [10% PEG400 in water (vol/vol)] containing 10 mg kg^-1^ d^-1^ twice daily (Bid) with a 6 h interval for one day. (c) Graphical representation of the amount of atglistatin in the lung and plasma of hamsters. For the detection of atglistatin in Golden Syrian Hamster, one group of 4 anesthetized hamsters were treated intraperitoneally with atglistatin in a 100 μL vehicle [50% PEG400 in water (vol/vol)] containing 80 mg kg^-1^ d^-1^ Bid with a 6 h interval for one day. Blood samples from the abdominal vena cava and lung samples of the experimental animals were collected 6 h after the last chemical treatment, and the concentration of atglistatin and CAY10499 in the samples were determined by LC-MS as described in Supplementary Information. Data are presented as the mean of 5 animals ± S.D.

**
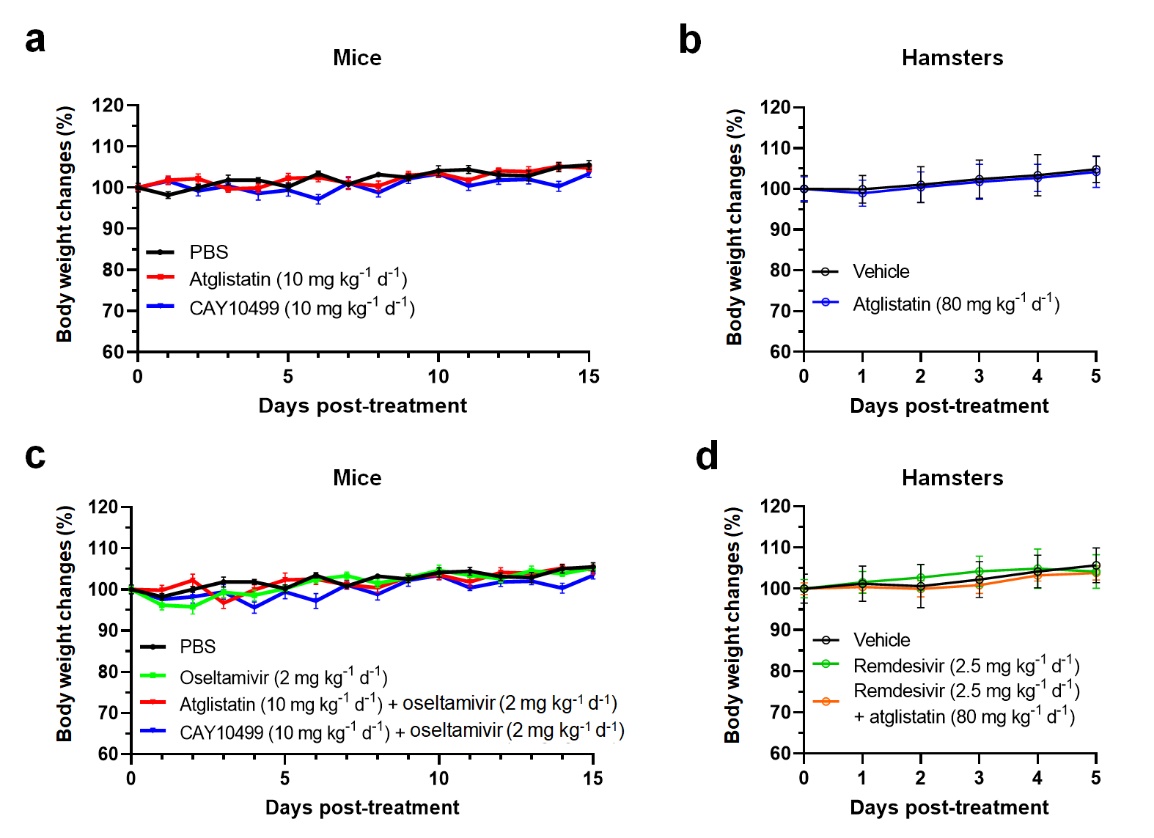
**

**Figure S14. *In vivo* toxicity of atglistatin and CAY10499 in mice and atglistatin in hamsters.** (a) Sixteen mice in each group were intraperitoneally injected with either atglistatin, CAY10499, or vehicle [10% PEG400 in water (vol/vol)] twice daily for four consecutive days; body weight was checked daily for 15 days. (b) Five hamsters in each group were intraperitoneally injected with either atglistatin or vehicle [50% PEG400 in water (vol/vol)] twice daily (Bid) for four and a half consecutive days and daily body weight was checked for five days. Shown is the body weight change of each group recorded for 15 days. The results are shown as the arithmetic means of the mean daily body weight ± S.D.


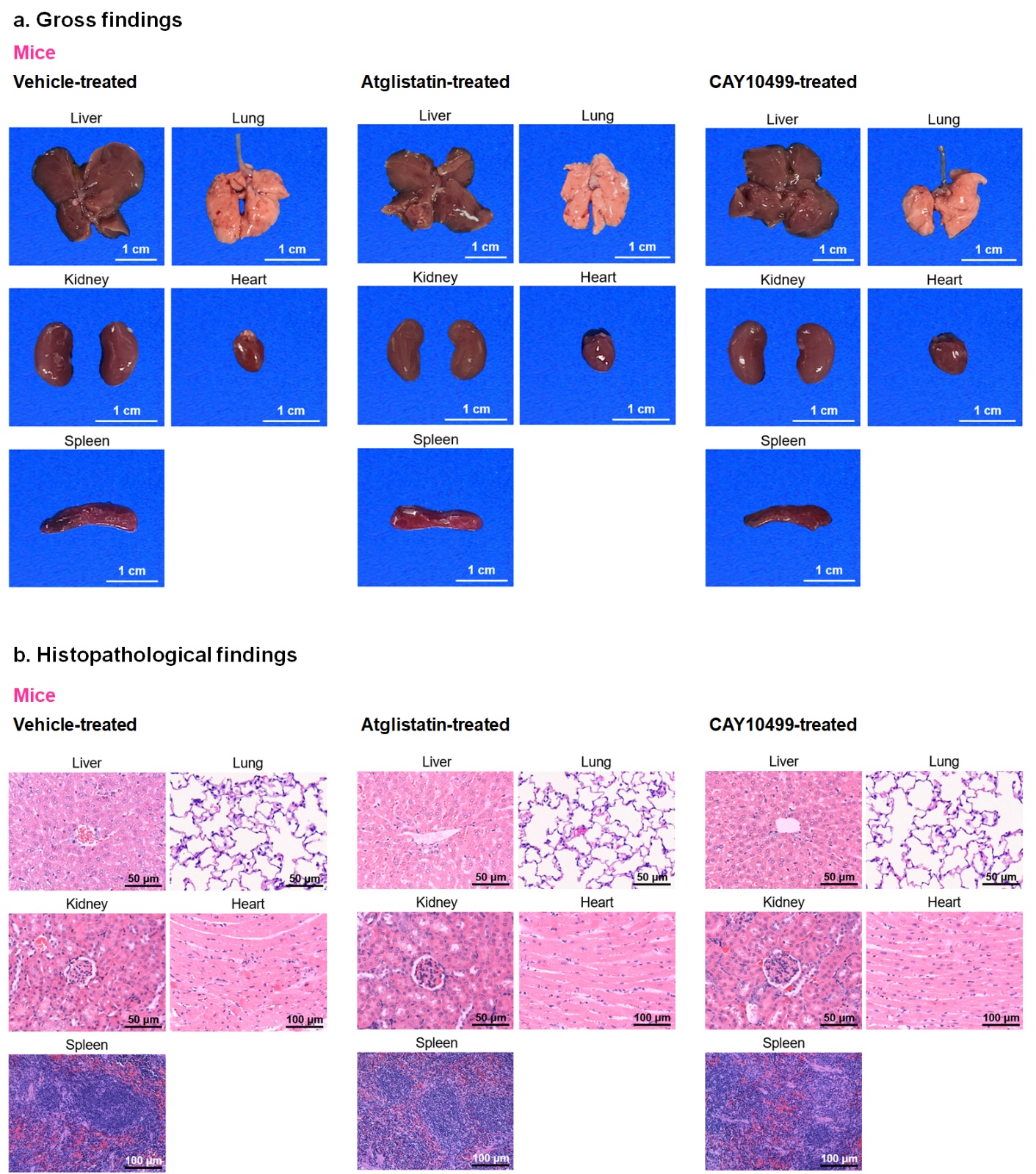


**Figure S15. *In vivo* organ-specific toxicity of atglistatin and CAY10499 in mice.** (a, b) Representative images of gross (a) and histopathological findings (b) of liver, lung, kidney, heart, and spleen in mice treated with 10 mg kg^-1^ d^-1^ atglistatin or CAY10499 or vehicle. For toxicological investigation of atglistatin and CAY10499 in wild-type C57Bl/6J mice, six groups of 3 anesthetized mice were treated intraperitoneally with either atglistatin or CAY10499 in a 50 μL vehicle [10% PEG400 in water (vol/vol)] containing 0.1, 1, or 10 mg kg^-1^ d^-1^ twice daily (Bid) with a 12 h interval for four consecutive days, respectively. One group of 3 control anesthetized mice was administered intraperitoneally with 100 μL vehicle Bid with a 12 h interval for four consecutive days. Each organ section was stained with hematoxylin and eosin (b). Scale bar = 1 cm (a), scale bar = 50 µm in liver, lung, and kidney in (b), and scale bar = 100 µm in heart and spleen in (b).


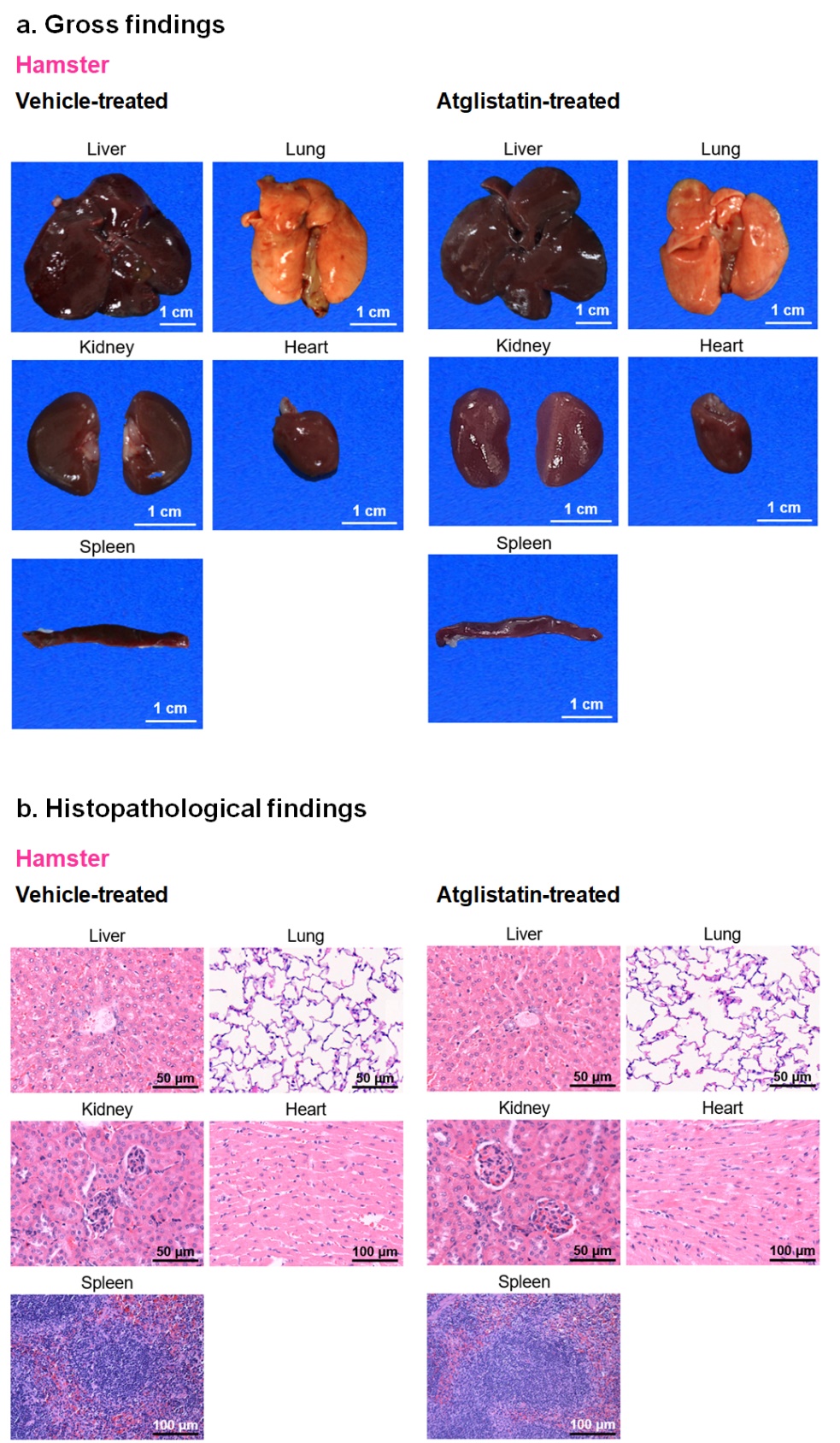


**Figure S16. *In vivo* organ-specific toxicity of atglistatin in hamsters.** (a, b) Representative images of gross (a) and histopathological findings (b) of liver, lung, kidney, heart, and spleen in hamsters treated with 80 mg kg^-1^ d^-1^ atglistatin or vehicle. For toxicological investigation of atglistatin in wild-type Golden Syrian Hamsters, three groups of 3 hamsters were treated intraperitoneally with 20, 40, and 80 mg kg^-1^ d^-1^ of atglistatin prepared in a 100 μL vehicle [50% PEG400 in water (vol/vol)] Bid with a 12 h interval for four and a half consecutive days, respectively. One group of 3 control anesthetized hamsters was administered intraperitoneally with 100 μL vehicle Bid with a 12 h interval for four and a half consecutive days. Each organ section was stained with hematoxylin and eosin (b). Scale bar = 1 cm (a), scale bar = 50 µm in liver, lung, and kidney in (b), and scale bar = 100 µm in heart and spleen in (b).

**
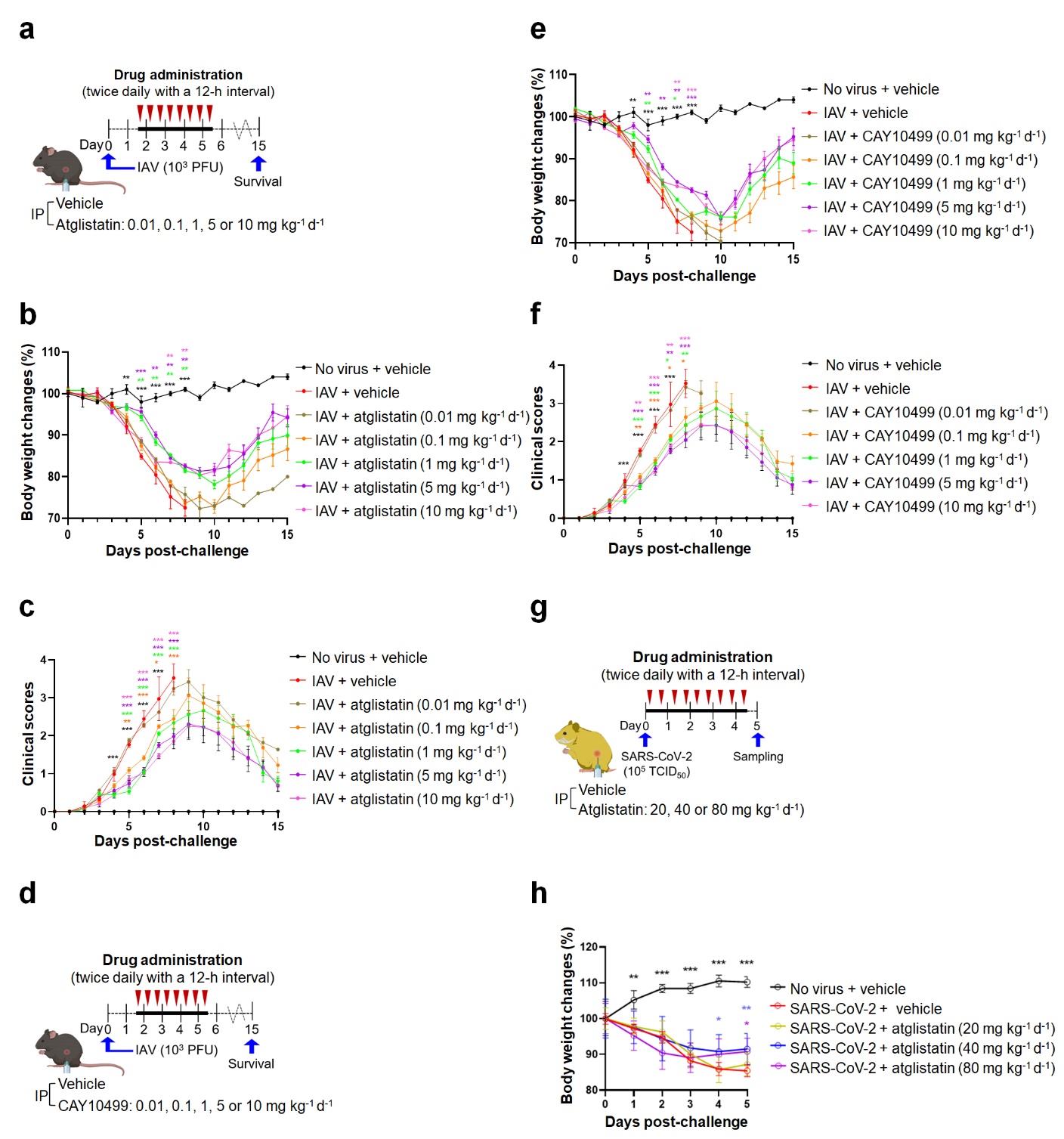
**

**Figure S17. Improvements in body weight gain and clinical scores in response to treatment with lipase inhibitors.** (a) Experimental scheme for determining the antiviral activity of atglistatin by intraperitoneal administration after challenge with 10^3^ PFU of mouse-adapted PR8 strain in mice. Each group (n = 16) was administered with different doses of atglistatin dissolved in vesicle [10% PEG400 in water (vol/vol)] or vehicle alone twice daily (Bid) for four consecutive days. (b) Daily body weight of IAV-challenged surviving mice treated with atglistatin at different doses. (c) Daily clinical sign score of IAV-challenged surviving mice treated with atglistatin at different doses. (d) Experimental scheme for determining the antiviral activity of CAY10499 by intraperitoneal administration after challenge with 10^3^ PFU of mouse-adapted PR8 strain in mice (n = 16). Each group was administered with different doses of CAY10499 dissolved in vesicle [10% PEG400 in water (vol/vol)] or vehicle alone Bid for four consecutive days. (e) Daily body weight of IAV-challenged surviving mice treated with CAY10488 at different doses. (f) Daily clinical sign score of IAV-challenged surviving mice treated with CAY10499 at different dosages. (g) Experimental scheme for determining the antiviral activity of atglistatin by intraperitoneal administration after challenge with 10^5^ TCID_50_ of SARS-CoV-2 KCDC03 strain in hamsters (n = 5). Each group was administered with different doses of atglistatin dissolved in vesicle [50% PEG400 in water (vol/vol)] or vehicle alone Bid for four and a half consecutive days. (h) Daily body weight of SARS-CoV-2-challenged surviving hamsters treated with atglistatin at different doses. Student’s t-test was used to compare different groups on each day post-infection. **P* < 0.05, ***P* < 0.01, ****P* < 0.001, *****P* < 0.0001.

**
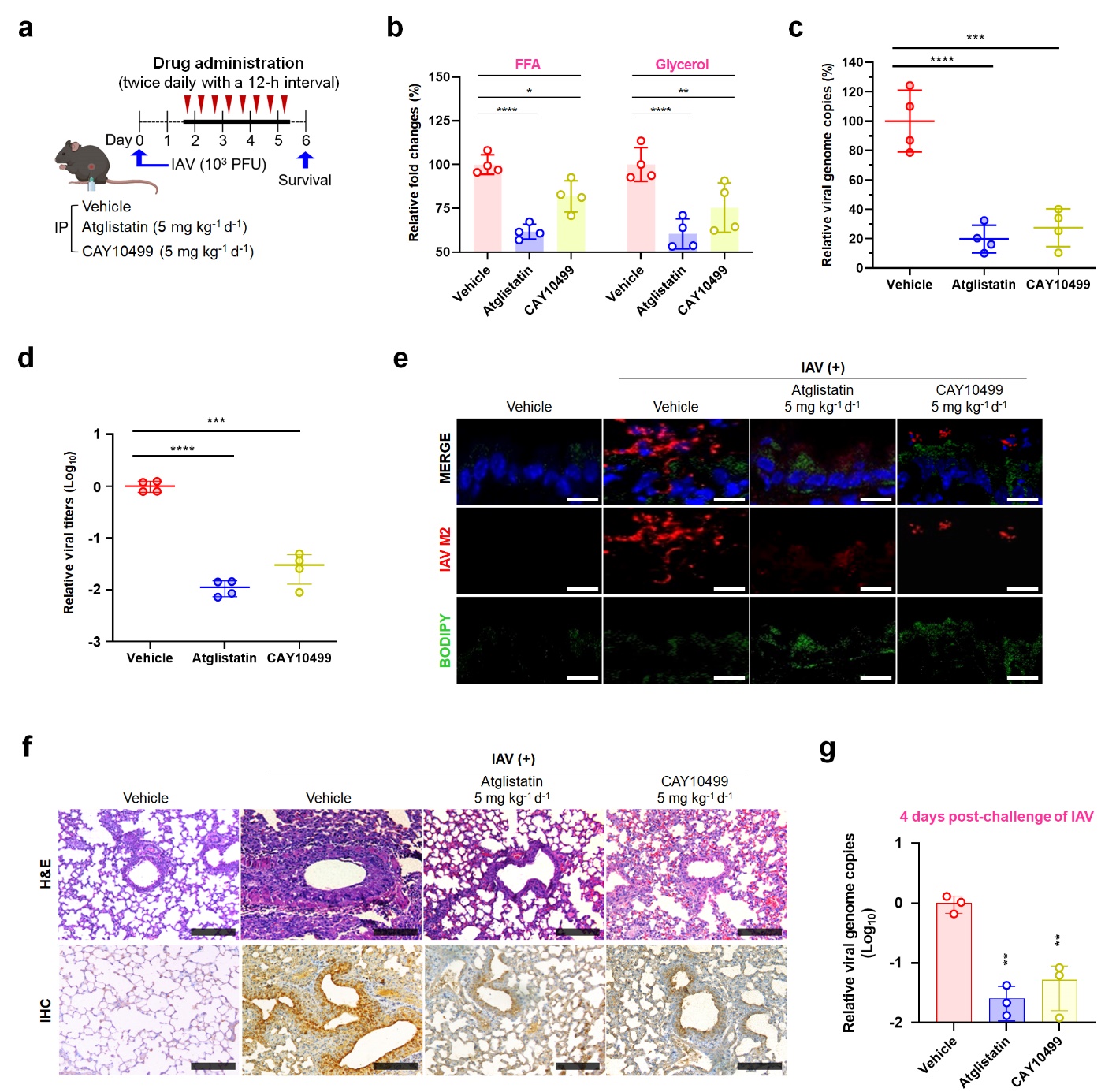
**

**Figure S18. *In vivo* antiviral and anti-viremia effects of lipase inhibitors against influenza A virus (IAV).** (a) Experimental scheme for determining the antiviral activity of atglistatin and CAY10499 by intraperitoneal administration after challenge with 10^3^ PFU of mouse-adapted PR8 strain to mice. Each group (n = 4) was administered with 5 mg^-1^ kg^-1^ day^-1^ of atglistatin or CAY10499 dissolved in vesicle [10% PEG400 in water (vol/vol)] or vehicle alone twice daily (Bid) for four consecutive days. (b) *In vivo* inhibition of FFA and glycerol releases from LDs in lung tissues sampled from IAV-challenged mice (n = 4) by treatment with atglistatin or CAY10499 twice daily for four consecutive days. (c, d) Reduction in IAV genome copy number and infectious progeny production in lung tissues sampled from IAV-challenged mice (n = 4) by treatment with atglistatin or CAY10499 Bid for four consecutive days. (e) Effect of atglistatin and CAY10499 on retention of LDs (green) and inhibition of IAV replication (red) in bronchiolar epithelial cells of lung tissues sampled from mice challenged with IAV. (f) Dose-dependent amelioration of histological lung lesions (upper panels) and inhibition of IAV replication (lower panels) by treatment with atglistatin or CAY10499. (g) Graphical representation of inhibitory effects of atglistatin and CAY10499 on the blood IAV load. Three groups of mice (n = 3) were challenged with intranasal inoculation of 10^3^ PFU of mouse-adapted IAV PR8 strain, and one group of mice (n = 3) were inoculated with 50 μL PBS as a negative control. Among the virus-challenged groups, two groups were administered with 5 mg kg^-1^ d^-1^ of atglistatin or CAY10499 Bid for four consecutive days via intraperitoneal injection. Mice in either mock-challenged, mock-administered or IAV-challenged, mock-administered groups were treated with 100 μL of vehicle Bid for four consecutive days via intraperitoneal injection. Blood samples were collected from the abdominal vena cava of experimental mice from each group at 4 dpi. Viral load in the blood was determined by RT-qPCR as described in Supplementary Information. Results are presented as arithmetic means ± S.D. *P < 0.05, **P < 0.01, ***P < 0.001, **** P < 0.0001, one-way analysis of variance with Tukey’s correction for multiple comparisons. Scale bars = 30 µm for panel i and 200 µm for panel j.

**
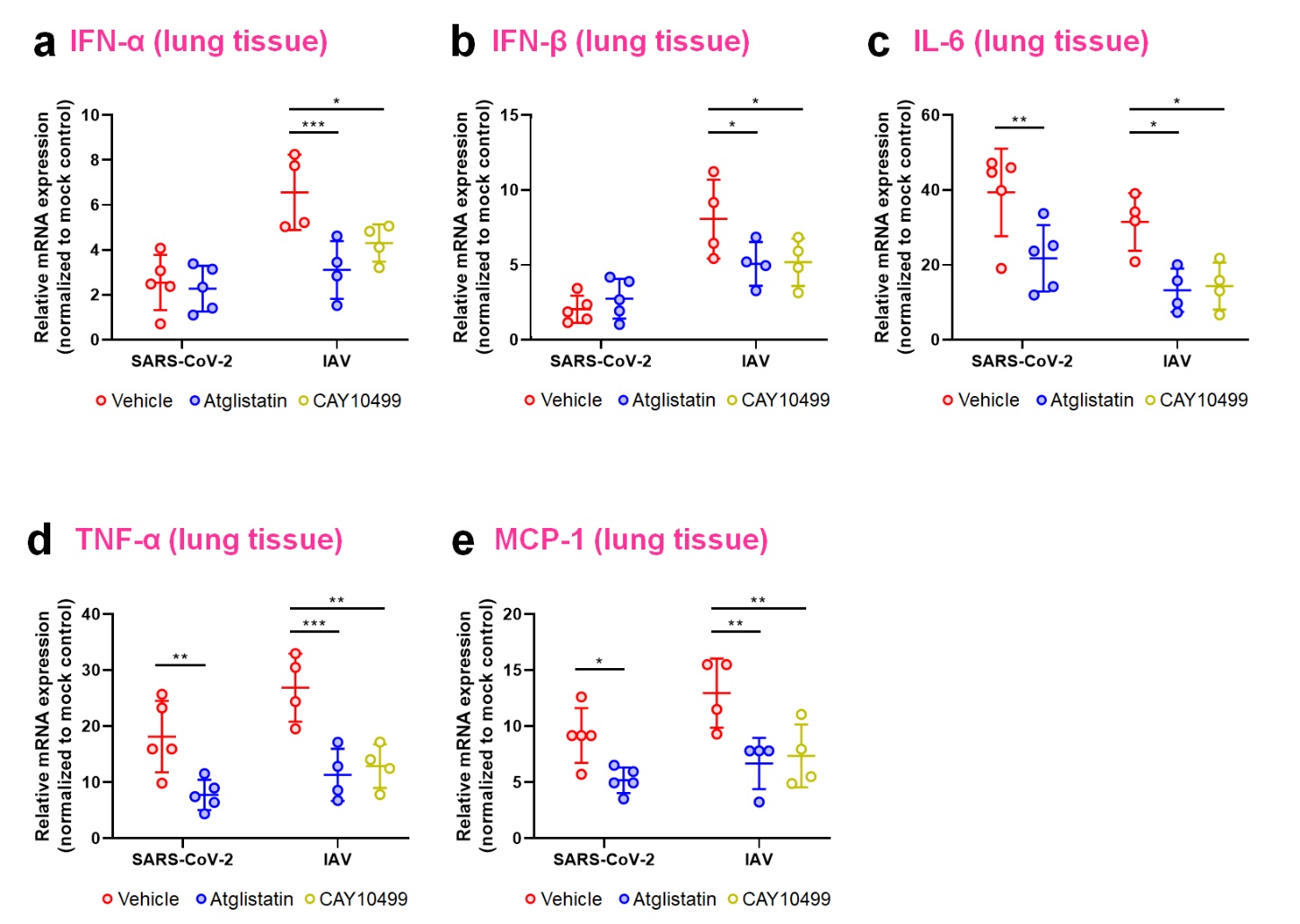
**

**Figure S19. Lipase inhibitors-induced alleviation of proinflammatory cytokine levels in SARS-CoV-2- or IAV-induced lung lesions.** (a-e) The graphical representation of the expression levels of IFN-α, IFN-β, IL-6, TNF-α, and MCP-1 genes in lung tissues harvested from SARS-CoV-2-challenged hamsters or IAV-challenged mice vehicle-treated or treated with atglistatin or CAY10499. The expression of each cytokine gene in lung tissues was determined by RT-qPCR as described in Supplementary Information. Results are presented as arithmetic means ± S.D. *P < 0.05, **P < 0.01, ***P < 0.001, **** P < 0.0001, one-way analysis of variance with Tukey’s correction for multiple comparisons.

**
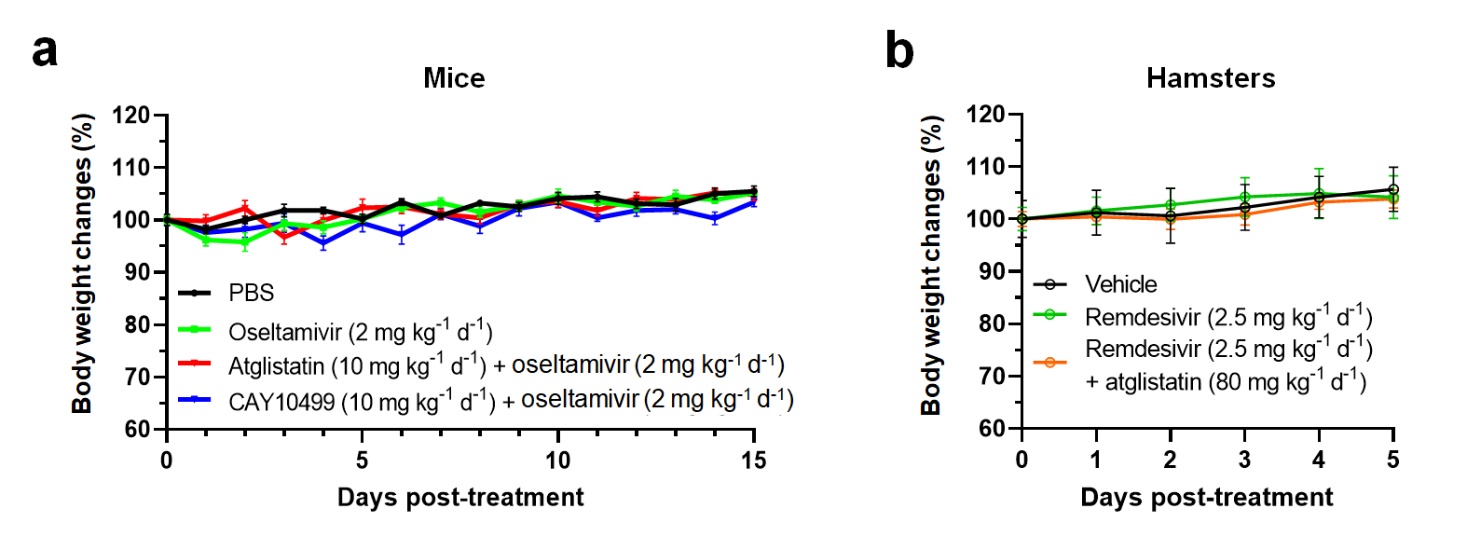
**

**Figure S20. *In vivo* toxicity of atglistatin and CAY10499 in mice and atglistatin in hamsters in combination therapy.** (a) Sixteen mice in each group were intraperitoneally injected with either atglistatin, oseltamivir, a mixture of atglistatin and oseltamivir, or vehicle [10% PEG400 in water (vol/vol)] for 15 days. (b) Five hamsters in each group were intraperitoneally injected with either atglistatin, oseltamivir, a mixture of atglistatin and oseltamivir, or vehicle [50% PEG400 in water (vol/vol)] Bid for four and a half consecutive days and body weight was checked daily for five days. Shown is the body weight change of each group recorded for 15 days. The results are shown as the arithmetic means of the mean daily body weight ± S.D.

**Original Films of Western Blots**

**Fig. 1d**

**
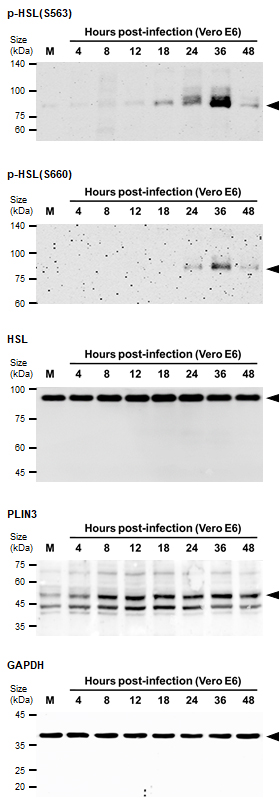
**

**Fig. 2m**

**
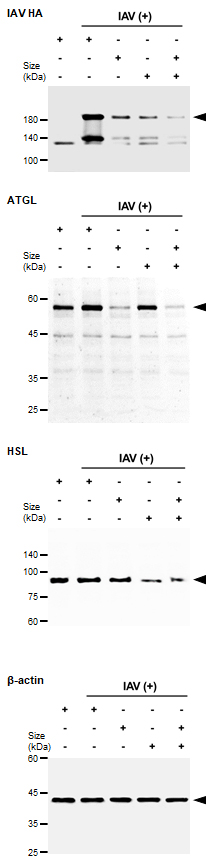
**

**Fig. 3h**

**
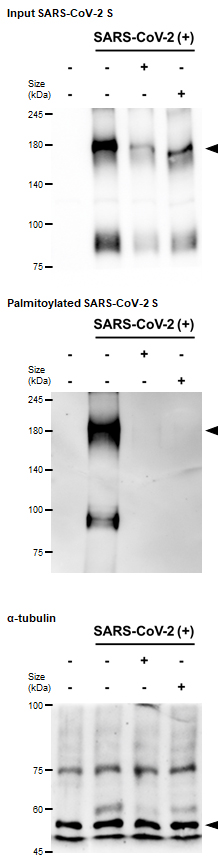
**

**Fig. S4c**

**
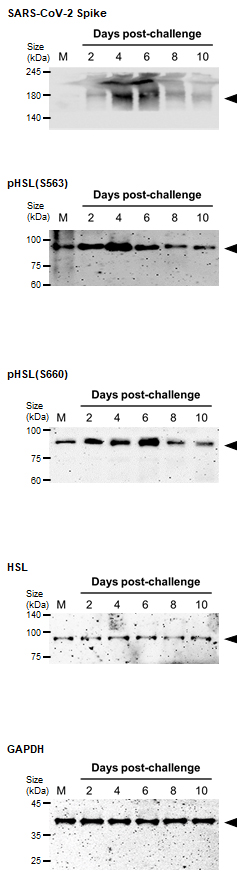
**

**Fig. S4d**

**
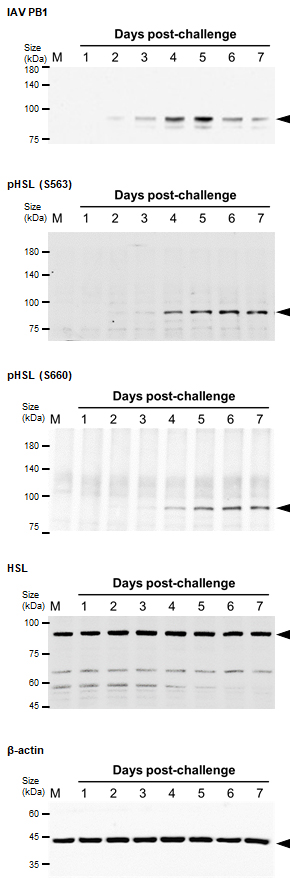
**

**Fig. S6c**

**
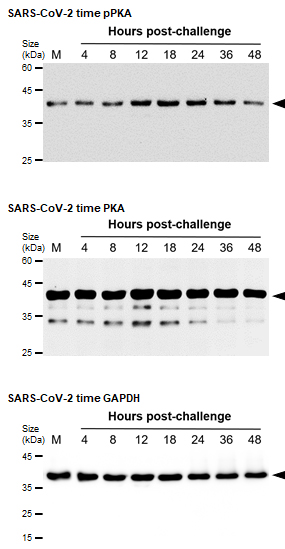
**

**Fig. S6d**

**
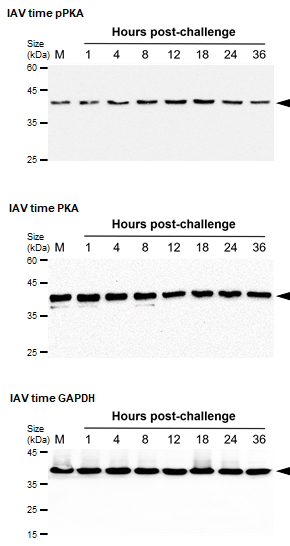
**

**Fig. S6e**

**
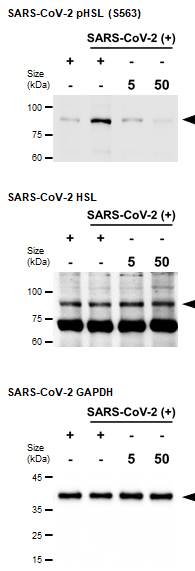
**

**Fig. S6f**

**
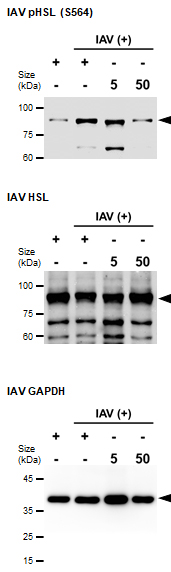
**

**Fig. S9c**

**
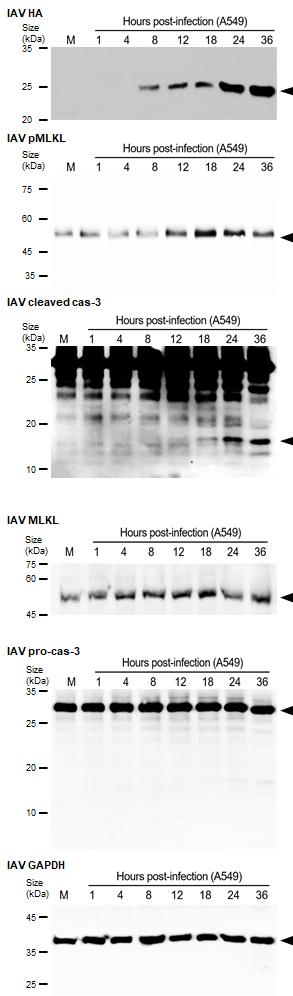
**

**Fig. S9d**

**
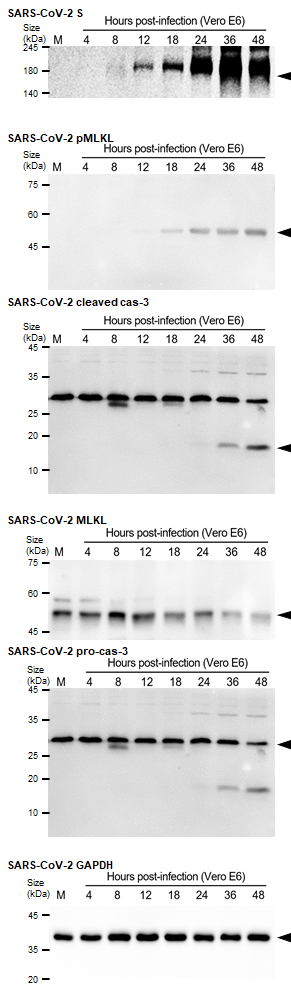
**

**Fig. S11b**

**
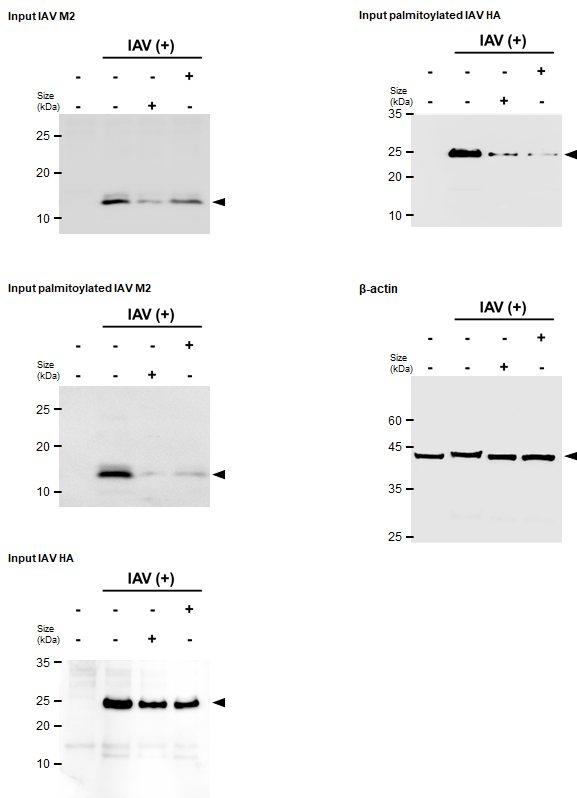
**
